# Healthy and productive workers: using intervention mapping to design a workplace health promotion and wellness program to improve presenteeism

**DOI:** 10.1186/s12889-016-3843-x

**Published:** 2016-11-25

**Authors:** Carlo Ammendolia, Pierre Côté, Carol Cancelliere, J. David Cassidy, Jan Hartvigsen, Eleanor Boyle, Sophie Soklaridis, Paula Stern, Benjamin Amick

**Affiliations:** 1 Institute of Health Policy, Management and Evaluation, University of Toronto, Toronto, Canada; 2 Institute for Work & Health, Toronto, Canada; 3 Mount Sinai Hospital, Toronto, Canada; 4 Dalla Lana School of Public Health, University of Toronto, Toronto, Canada; 5 Department of Sports Science and Clinical Biomechanics, University of Southern Denmark, Odense, Denmark; 6 Nordic Institute of Chiropractic and Clinical Biomechanics, Odense, Denmark; 7 Canadian Memorial Chiropractic College, Toronto, Canada; 8 Centre for Addiction and Mental Health, Toronto, Canada; 9 University of Ontario Institute of Technology, Toronto, ON Canada; 10 Robert Stempel College of Public Health and Social Work, Miami, FL USA

**Keywords:** Presenteeism, Health promotion, Workplace wellness, Intervention mapping, Workplace health, Qualitative study, Work productivity

## Abstract

**Background:**

Presenteeism is a growing problem in developed countries mostly due to an aging workforce. The economic costs related to presenteeism exceed those of absenteeism and employer health costs. Employers are implementing workplace health promotion and wellness programs to improve health among workers and reduce presenteeism. How best to design, integrate and deliver these programs are unknown. The main purpose of this study was to use an intervention mapping approach to develop a workplace health promotion and wellness program aimed at reducing presenteeism.

**Methods:**

We partnered with a large international financial services company and used a qualitative synthesis based on an intervention mapping methodology. Evidence from systematic reviews and key articles on reducing presenteeism and implementing health promotion programs was combined with theoretical models for changing behavior and stakeholder experience. This was then systematically operationalized into a program using discussion groups and consensus among experts and stakeholders.

**Results:**

The top health problem impacting our workplace partner was mental health. Depression and stress were the first and second highest cause of productivity loss respectively. A multi-pronged program with detailed action steps was developed and directed at key stakeholders and health conditions. For mental health, regular sharing focus groups, social networking, monthly personal stories from leadership using webinars and multi-media communications, expert-led workshops, lunch and learn sessions and manager and employee training were part of a comprehensive program. Comprehensive, specific and multi-pronged strategies were developed and aimed at encouraging healthy behaviours that impact presenteeism such as regular exercise, proper nutrition, adequate sleep, smoking cessation, socialization and work-life balance. Limitations of the intervention mapping process included high resource and time requirements, the lack of external input and viewpoints skewed towards middle and upper management, and using secondary workplace data of unknown validity and reliability.

**Conclusions:**

In general, intervention mapping was a useful method to develop a workplace health promotion and wellness program aimed at reducing presenteeism. The methodology provided a step-by-step process to unravel a complex problem. The process compelled participants to think critically, collaboratively and in nontraditional ways.

**Electronic supplementary material:**

The online version of this article (doi:10.1186/s12889-016-3843-x) contains supplementary material, which is available to authorized users.

## Background

Presenteeism refers to the loss of work productivity among workers who are present at work, but limited in some aspect of job performance by a health problem [1]. Developed countries around the world face a major challenge in maintaining a healthy and productive workforce. A main reason for this challenge is a combination of declining birth rates and increasing longevity, which have resulted in an aging workforce around the world. In Canada, it is estimated that by the year 2026, one in five Canadians will be 65 years of age or older, up from one in eight in 2001 [2]. In the US, the median age of the civilian labour force was 35 in 1984 and is expected to reach 42 in 2014, with 21% of the workforce 55 years and older [3].

Recent literature on aging and the workforce revealed that older workers experience physical changes that may negatively affect their work. Such physical changes include: loss of muscular strength and range of joint movement, decreased ability to maintain good posture and balance, reduced ability to regulate sleep, and reduced vision and auditory capabilities [4]. In addition, aging workers experience an increased prevalence of ill health including diabetes, cardiovascular disease, depression, arthritis, and back pain. Many will have multiple health problems that will impact their quality of life and ability to perform on the job [1, 5, 6].

Presenteeism is often a hidden cost, as workers are physically present but unable to perform at peak levels due to a health condition. A study of ten common health conditions found that presenteeism-related costs were greater than direct health costs in most cases, and they accounted for 18–60% of all costs for each of the ten conditions [7]. In the US, presenteeism costs are estimated to be in excess of $180 billion per year, compared to the $118 billion costs related to absenteeism [7]. As organizations and employers become more aware of this particular type of productivity loss and its significant economic implications, they are looking to workplace health promotion and wellness programs aimed specifically at presenteeism [8].

Workplace health promotion and wellness programs vary considerably in size and composition. Comprehensive programs provide health education, links to related employee services, supportive physical and social environments for health improvement, integration of health promotion into the organization’s culture, and employee screening with adequate treatment and follow up [8]. However details on how best to design, integrate, tailor and deliver programs to reduce presenteeism are unknown.

Intervention mapping is a method for developing and designing complex interventions or programs [9]. Although traditionally used to develop community health promotion and disease prevention programs such as AIDS prevention [10] and smoking cessation programs [11], intervention mapping may be well suited for designing workplace interventions or programs [12–14]. This is because workplace programs are also complex, necessitating a tailored and multifaceted approach directed at various stakeholders and settings [15].

New integrated and tailored approaches are urgently needed to curb the increasing prevalence, economic cost and personal burden of presenteeism. The main purpose of this paper was to describe the application of the intervention mapping approach to the development of a health promotion and wellness program aimed at reducing presenteeism in an international financial services company with over 8000 employees in Canada. The study objectives included; i) establishing the need to reduce presenteeism with our workplace partner and prioritize their specific high risk health conditions, ii) detailing the intervention mapping steps and outcomes and iii) outlining the strengths and limitations of the intervention mapping approach.

## Methods

### Intervention mapping

An intervention mapping methodology was used for this study. Intervention mapping is a systematic and comprehensive approach to evidence and theory based program development with the aim of using stakeholder involvement to tailor interventions/programs to suit the needs of a specific population [9, 16].

We approached a large international financial services company with over 8000 employees in Canada. This workplace was in the process of re-designing their health promotion and wellness program and interested in maximizing employee health and reducing presenteeism. The workplace agreed to partner with our research team and participate in the intervention mapping process to re-design their program. The setting for this study was the Canadian head and corporate offices located in Toronto and Southwestern Ontario, Canada. The participating workplace was non-unionized and all participants in this study were adults and provided written informed consent.

**Fig. 1:**
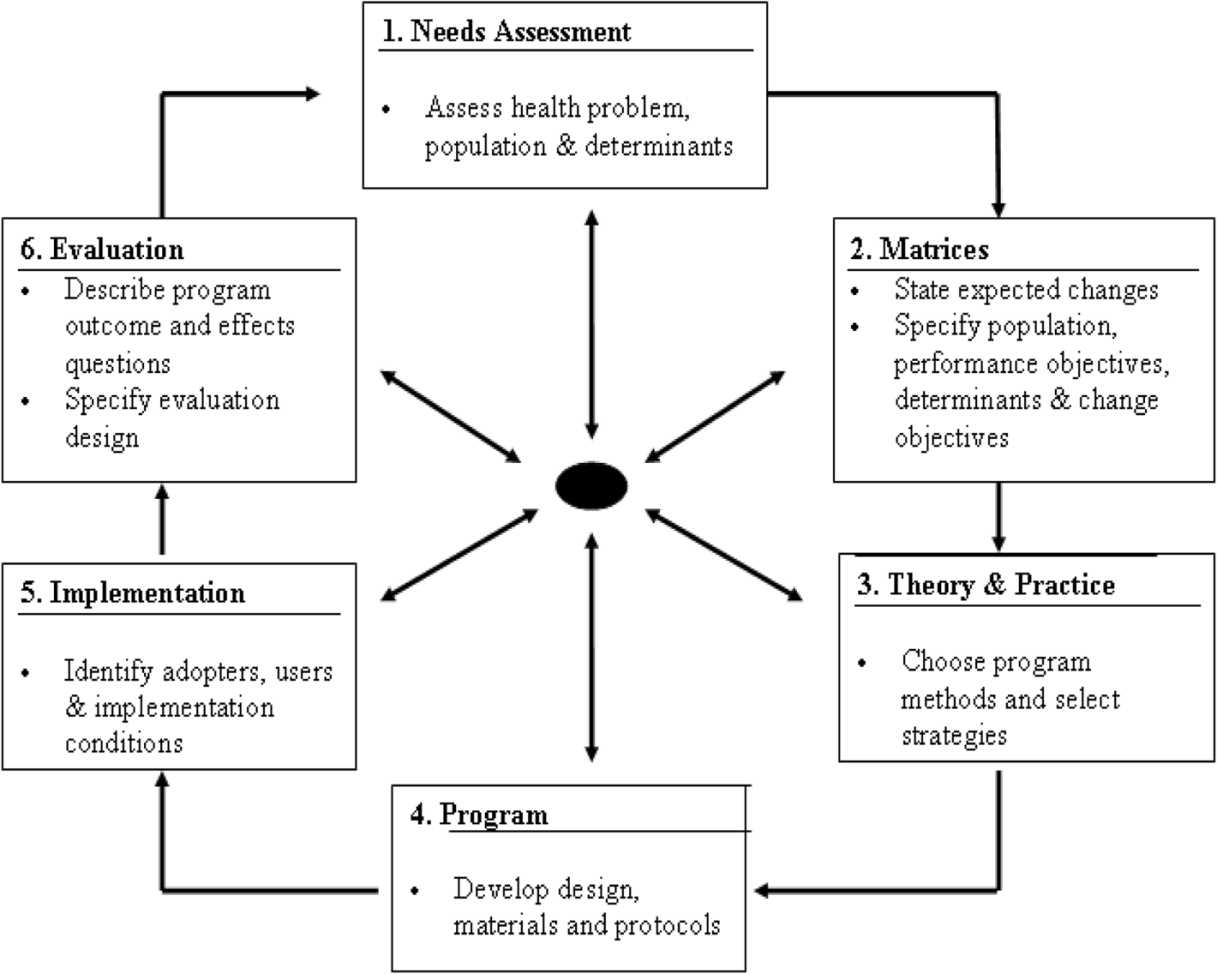
Intervention Mapping Framework, adapted from Bartholomew et al. [9]

There are six steps in intervention mapping. Step 1 consists of a needs assessment; steps 2, 3 and 4 involve the initial development of the program; step 5 consists of planning for implementation; and step 6 involves evaluation and refinement of the program. In this study we performed steps 1 to 4. Figure 1 depicts the intervention-mapping framework.

Within each step of intervention mapping, specific tasks were performed and questions answered, which guided the decision making process. These tasks were accomplished systematically using core processes [9]. Core processes involved brain-storming among a selected group of individuals (known as the intervention mapping team made up of researchers, content experts and work-related stakeholders), who came up with provisional solutions to the specific tasks and questions. Provisional solutions were achieved by group consensus and based on the best available evidence, theories on presenteeism, workplace health promotion, wellness programs and practical experiences of participating stakeholders. Below is an outline of each of the intervention mapping steps.

### Step1. Conduct a needs assessment and select intervention team

The objectives of the workplace needs assessment were to i) establish the rationale to reduce presenteeism, ii) define the population of interest, and iii) list and prioritize the most important health conditions impacting presenteeism and their underlying individual and environmental risk factors.

Data used for the needs assessment included the following:

1. Administrative and claims data (2010) provided by the workplace partner. This included demographic information, reasons and duration of absenteeism, reasons and duration of short-term and long-term disability claims, and type of drug and indication for prescription drug claims. The validity of the data provided by the workplace was not examined.
2. Summary findings from the online employee wellness surveys (2009 and 2010) provided by the workplace partner. This was an in-house voluntary survey sent to all employees. The survey summary data included an inventory of risk factors for ill health, the Work Productivity and Activity Impairment Questionnaire (WPAIQ) [19] and the Business Health Culture Index (BHCI) [20]. The WPAIQ is a validated measure of health related productivity loss in the workplace. Respondents are asked questions about work impairment due to health problems including depression, stress, cardiovascular disease, diabetes and musculoskeletal pain [19]. The BHCI is used to calculate stress versus satisfaction levels present among an employee population. It is a valid reliable indicator of work culture and has been shown to correlate to other organizational factors such as engagement, absenteeism, presenteeism, retention and disability [20]. The accuracy of the WPAIQ and BHCI summary scores provided by the workplace was not assessed.
3. Four one-on-one 60 min interviews and four 90-min discussion group sessions with workplace participants conducted by the research team. Open-ended questions were used during the interviews and discussion group sessions. Examples of open-ended questions included the following:
  What are the challenges of the existing health promotion program?
  What are the important health issues impacting presenteeism?
  What jobs/individuals are at risk for presenteeism?
  What are important individual and environment risks factors impacting presenteeism?

Interview and discussion group participants were selected in consultation with the workplace partner with an attempt to include a mix of representation (of views and status) of employees across the organization.

The interviews and discussion groups were audio recorded and transcribed verbatim. Two researchers independently reviewed, coded the transcripts, and extracted themes using ground theory approach [17]. A consensus needs assessment summary was produced.

Following the needs assessment, an intervention mapping team was assembled consisting of researchers, workplace directors, managers, and consultants involved in benefits and health and wellness, supervisors and front line client representatives. The team members were selected based on their experience in health promotion, the potential to provide varied perspectives and the ability to commit to the time obligations of the study. A note taker at each session displayed all responses to questions/tasks electronically in real time. Consensus was achieved during each discussion group meeting or electronically through email exchange following the distribution of discussion topic materials.

### Step 2. Develop program objectives

The first task in Step 2 was to list all-important stakeholders that can impact presenteeism. This was followed by listing performance objectives and the expected outcome for each identified stakeholder and prioritized health conditions impacting presenteeism. Performance objectives are necessary activities that each stakeholder should perform to reduce presenteeism. Each performance objective was then matched with modifiable individual and external (environmental) determinants. Determinants act as barriers or facilitators for achieving performance objectives. Individual determinants were classified into four groups; knowledge, capability or skills, cognitive-behavioral (attitudes, beliefs and emotions), expectations and self-efficacy. External determinants were classified into five groups; norms and policies, social support, reinforcement, resources and organizational climate. Using the list generated for performance objectives and matching list of determinants, a matrix (performance objectives vs. determinants) was constructed for each stakeholder and priority health condition. In the body of the matrix, who and what needs to change and/or be learned (known as learn/change objectives) to achieve the objectives were outlined. The goal of step 2 was to identify for each important stakeholder and specific health condition all potential barriers and facilitators and their corresponding change and/or learned objectives.

### Step 3. Develop theoretical methods and practical strategies

The purpose of step 3 is to use core processes and list potential (practical) strategies for each change and/or learned objective for each stakeholder and priority health condition listed in step 2. Listed strategies or interventions would be based on evidence derived from the published literature, theories on how to change human behavior and the experiences of intervention mapping team members.

### Step 4. Design a workplace health promotion and wellness program

In step 4 the goal was to operationalize the practical interventions and strategies compiled in step 3 into a workplace health promotion and wellness program with discrete components, mechanisms of delivery and timelines. The intervention mapping team achieved this using core processes. During Step 4 a gap analysis [18] was performed comparing recommended interventions and practical strategies to current practice.

## Results

### Step 1. Needs assessment

The workplace provided aggregate demographic and claims data (2010) and summary scores for the Wellness Assessment Survey (2009 and 2010).

Our workplace partner had over 8000 employees of which about 75% were female. About 45% of the workforce was between 40 and 50 years of age. The top four health problems that impacted our workplace partner, based on disability and prescription medication claims included mental health (45% of all long-term claims and 33% of all short-term claims), musculoskeletal (17 and 12% for long and short term disability), diabetes and cardiovascular disease.

Participation rates for the online wellness surveys were 31 and 12% for the years 2009 and 2010 respectively. The workplace partner did not provide reasons for the drop in participation in 2010. In 2009 and 2010, the top five reported risk factors for ill health (moderate to severe risk) included nutrition, sleep, stress, physical exercise, and weight. Over 60% of participants had 3 or more risk factors. The WPAIQ [19] scores suggested that depression and stress were the first and second highest cause of productivity loss with 41 and 54% of participants at moderate or high risk respectively. In 2009 and 2010 the BHCI [20] scores suggested employees were more stressed than satisfied and perceived to be unfairly treated by the employer.

#### Summary of discussion group and interview sessions

A description of the job titles of employees participating in the interviews and discussion groups is outlined in Table 1. Each discussion group session consisted of mix representation of 8–10 employees. Table 2 summarizes responses to the questions asked during the discussion group sessions and interviews as part of the needs assessment.

**Table 1:** Job titles of interviews and discussion group participants

Discussion Groups Participants (*N* = 37)	
Top executives Human Resources Director - Benefits Assistant Vice President – Total Benefits Assistant Vice President – Group Benefits Human Resources Director – Business Middle management Manager Disability Benefits Manager Human Resources (*n* = 3) Contract Writer - Benefits Account Executive - Business Account Executive (*n* = 2) Research Specialists (*n* = 2) Employment Practices Consultant Coordinator for Strategic Projects Senior Medical Benefits Analyst Auditor Benefits Payments Manager of Strategic Projects Frontline consultants Health Management Consultant Group Disability Consultants (*n* = 3) Claims Consultants (*n* = 3) Group Retirement Benefits Consultants (*n* = 2) Disability Consultants – Benefits (*n* = 3) Disability Consultants (*n* = 3) Medical and Dental Claims Consultants (*n* = 3)	
Interviews *N* = 4	
Human Resources Director - Benefits Supervisor, Claims benefits Benefits Manager Claims Consultant

**Table 2:** Needs Assessment: Summary of interviews and discussion groups

What are the challenges of the existing health promotion program? Call Centre and Front-line Consultants • High work demand and lack of time and support from managers significantly impacted participation in health and wellness initiatives • Participation in wellness programs would be perceived negatively by co-workers and managers, and would impact their workload • Very strong focus on production with no time for anything else • Sense that wellness is not valued by organization as much as production Executives and Managers • Lack of employee engagement and potential reasons lack of awareness and/or inadequate communication • Need for a “culture shift” from the current reactive approach to a more “proactive wellness approach,” • Wellness was low on the priority list among senior leadership • Performance reviews that were focused on production, with wellness receiving little to no attention • Data on “Return on Investment” on workplace health promotion and wellness programs were lacking and were essential to get additional resources from senior management • Current program was event-driven; there needs to be a consistent and sustainable level of awareness and participation, and a need to better integrate the various wellness initiatives throughout the organization • Lack of adequate training of managers in health promotion, the inability to identify and manage high-risk employees, and the need for more role models/mentors among management and respected peers Enhance the current incentive strategies for employees and the need to measure their effectiveness
What are the important health issues impacting presenteeism? • Mental health issues including stress, anxiety, and depression were the most dominant health problems impacting presenteeism • Musculoskeletal conditions, diabetes, cardiovascular disorders, cancer, upper respiratory infections, and headaches were also important
What jobs/individuals are at risk for presenteeism? • Every employee was at risk for presenteeism and that health promotion programs should be directed at all employees • Jobs with high work demand and low decision latitude were the most vulnerable • Individuals with high work demand and high decision latitude were also at risk because their decisions impact the livelihood of many employees
What are important individual risk factors impacting presenteeism? • High work/life stress, physical inactivity, poor nutrition habits, obesity, lack of sleep, smoking and social isolation were all important individual risk factors
What are important environmental risk factors impacting presenteeism? • Workplace stress/demand, workplace culture, sitting posture (on phone/computer, poor ergonomics), long commuting time, shift work, cafeteria options and the added stress due to poor economy which impact company profits

### Step 2. Develop program objectives

**Fig. 2:**
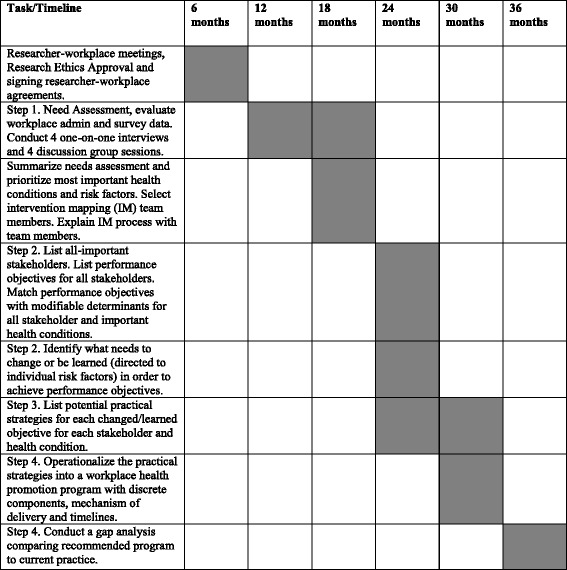
Study Timeline

The intervention mapping team met approximately 14 times over an 18-month period (2012–2013) and worked through Steps 2–4. A timeline of the intervention mapping process is outlined in Fig. 2.

#### Who are the important stakeholders impacting presenteeism?

The intervention mapping team identified the following important stakeholders that can impact presenteeism: 1) the employee, 2) co-workers, 3) managers and supervisors 4) senior management/organization, 5) spouse/partner/other family members and 6) family doctor and other health care providers. Important health conditions impacting presenteeism were prioritized and categorized as mental health, musculoskeletal, cardiovascular and diabetes, cancer, and the flu.

#### What are the performance objectives (measure) for each stakeholder?

Performance objectives are listed for each stakeholder and health condition in Appendix A. An example of performance objectives for the employee and mental health are outlined in Table 3. For mental health, de-stigmatization and open communication were important performance objectives for all stakeholders. Seeking positive relationships, avoiding isolation and, engaging in stress management and work/life balance were performance objectives listed for the employee/co-workers for mental health. Training in mental health was a key measure for managers and supervisors. Engaging in healthy behaviors such as regular exercise, adequate sleep, proper nutrition and the avoidance of smoking and excessive alcohol were listed across most priority health conditions.

**Table 3:** An example of performance objectives (measures) for the employee (stakeholder) to reduce presenteeism for mental health (health condition)

1.	The employee participates in the de-stigmatizing of mental health disorders
2.	The employee learns to openly communicate issues around mental health
3.	The employee knows when to seek help for mental health issues
4.	The employee seeks out positive relationships with peers and leadership persons
5.	The employee knows where to seek out available resources about mental health
6.	The employee participates in social networks and minimizes isolation
7.	The employee avoids gossip and other negative behaviours
8.	The employee is compliant with medication/counselling/support
9.	The employee discusses with his/her manager any limitations because of mental health issues
10.	The employee engages in healthy behaviours such as, 30 min of exercise per day, adequate sleep, follows a healthy diet, avoids tobacco and minimizes alcohol
11.	The employee engages in stress/time management and work/life balance

Awareness of available resources was another key performance objective for the employee, co-workers and manager/supervisor. For the organization, benchmarking was a common performance objective (measure) listed across health conditions, as well as the need to establish a mission statement, philosophy and culture around the importance of employee health. Investing in social capital was also an important performance objective for the organization.

#### What are the learn and change objectives?

Individual and environmental determinants of the listed performance objectives (measures) for each stakeholder and priority health condition, and the required learn and change objectives (actions directed to individual and environmental determinants in order to achieve the performance objectives) are outlined in Matrices A to E located in Additional file 1: Appendix B. An example of learn and change objectives for the employee and mental health is outlined in Table 4. Learn and change objectives for de-stigmatizing mental health included increasing knowledge on the importance of open communication, the need to change attitudes, beliefs and de-mystifying stigma around mental health. Providing knowledge on available resources for the employee and managers and changing attitudes around willingness to seek social support were other key learn/change objectives for mental health. Change/learn objectives aimed at external determinants for mental health included having a workplace culture that encourages open communication, compassion, positive relationships and social interaction and an organization that provides necessary resources for training, awareness and employee mental health initiatives. This supportive workplace culture that makes employee health a priority was an important change objective for all priority health conditions.

**Table 4:** An example of a matrix of the learned and change objectives for mental health

Mental Health – Employee		Individual Determinants		
Performance Objectives	Knowledge, capability or skill	Attitudes, beliefs and values	Expectations	Self efficacy
Destigmatize MH Look at MH differently Openly communicate	Describe that it is okay to talk about depression/anxiety/mental health issues Inform all employees about mental health issues	Explain/de-mystify the stigma around mental health	Demonstrate that by talking about my MH problems, I will get empathy or understanding from my organization/peers/etc.	Build confidence in the ability to discuss MH problems
Know when to seek help	Describe or explain what help is available to them Explain there are resources available that will tell them how to seek help (who, where to go)	Explain that seeking help will be confidential Explain or describe that MH can be effectively treated	Demonstrate that people who seek help can be helped	Provide resources to foster self-confidence in people to know when and where to seek help
Seek out positive relationships among peers and leadership	Ensure the leaders know what is expected from them Inform employees about the role leaders should play Encourage employees to discuss MH issues Explain the role of positive relationships in MH Inform that leadership has been trained in MH		Demonstrate that the leadership will be supportive of MH issues	Improve confidence in their willingness to seek leadership advice

*MH* mental health

### Step 3. Develop Theoretical Methods and Practical Strategies

#### What are the practical strategies needed?

Translating learn and change objectives into practical strategies is summarized in Additional file 2: Appendix C. An example of translating learn and change objectives into practical strategies using the employee and mental health is outlined in Table 5. The discussion group participants decided to operationalize this task by listing both practical strategies and big picture “pie in the sky” strategies without concern for structural, organizational or financial limitations. The purpose of this approach was to stimulate creative “outside the box” thinking, which could result in potentially unique approaches to translating and/or implementing learn and change objectives. Practical strategies and big ideas were then contextualized in terms of how they could be implemented into current practice. The participants also decided to group important health conditions into Mental Health and General since mental health was identified as a main priority.

**Table 5:** An example of learned and change objectives that have been translated into practical strategies for mental health

Objectives What needs to be done/what needs to be changed?	Methods How can these objectives be accomplished?		Practical Applications
	Best practices and initial ideas	Big ideas There are no wrong answers – think creatively! What would be the ideal activities to implement if there were no limitations (e.g. structural, organizational, financial, etc.)?	How can we implement this in our workplace and when could we implement? If it’s already being done today, how can we improve it?
Employee			
Inform all employees about mental health issues Explain/de-mystify stigma around mental health Build confidence to to discuss mental health problems Demonstrate that leadership will be supportive of mental health issues Educate employees on when to seek help and find resources	Use structured multi-pronged educational interventions Use The Source Include applicable information in the new employee manual Have lunch and learns with content expert guest speakers such as psychologists and behavioural therapists Add MH destigmatization training for employees Have leadership communicate with employees regularly (scheduled and anticipated) using multi-media and personal stories-clear simple caring messages Identify and train respected peers respected peers to act as health ambassadors Use Health Coaching/nurse or lay health worker, have onsite health centre Use The Source website to provide interactive cognitively based self help programs, chat room and access to consult with professional/personal coach available for tailoring program	Create forums where employees can talk about mental health and receive input from other employees around de-stigmatizing mental health Have on-demand mental health resources available such as an online depression centre geared towards low to moderate risk employees.	Leverage the mental health portal that we are building for clients (communication, education, blogging) – in final draft currently (ensure alignment and integration); social networking may be part of this portal as a later component Focus groups – provide opportunities to incorporate as advanced/progressive element of training program. Include managers and employees. Pilot as an add-on Disability expert could facilitate using their Mental Health First Aid however; may eliminate some of the sensitivities by having outside body facilitate Identify leader with a personal story (could be internal leader, or could be an external leader/athlete/person in the public eye) Part of program: extend manager training to employees. Use source to tell peoples’ stories; use to change behaviours/attitudes; leverage the Quick Polls to include questions around mental health to increase awareness

*MH* mental health

Practical strategies for de-stigmatizing mental health would be part of a companywide mental health communication strategy. This included structured, multi-pronged, multi-media educational interventions aimed at improving knowledge and self-confidence, and changing attitudes and beliefs. Components of this strategy included the use of the company intranet to dispel misinformation on mental health, to serve as a medium for testimonials and personal stories of employees dealing with mental health, and to provide a potential source for online discussion groups. Focus groups and lunch and learn events facilitated by experts for employees to discuss mental health issues could be implemented into current practice. A corporate interactive website for open communication and dialogue about mental health and other priority health conditions is also possible. Training health/wellness ambassadors and workplace opinion leaders to act as role models to change attitudes and beliefs around mental health was also suggested.

For managers and supervisors, role-playing as part of their training may help re-enforce and improve skills in the identification and implementation of strategies for mental health and other priority health conditions. This peer-to-peer interaction could facilitate team building and social support. Mandatory training for managers on all priority health conditions and regular and effective communication between senior management and managers through webinar or video conferencing were also suggested. A “Big Idea” strategy included having senior management share personal stories related to a mental health, or other health conditions. Having a highly notable and respected person in the organization champion a workplace health initiative could increase engagement, add credibility, and promote a positive workplace health culture. Another “Big Idea” would be to incorporate health objectives into annual performance plans. This could facilitate a philosophy that aligns “health with wealth.” Designing and implementing Health Score Cards and incorporating Health Audits to provide feedback and benchmarking on health status were other listed strategies.

A key strategy for the organization was to collect valid data and to use this data to support health initiatives and demonstrate how these initiatives can improve the bottom line and provide a business case for additional resources.

### Step 4. Designing a workplace health promotion and wellness program (improving current program)

The next step in the intervention mapping process was to operationalize the practical strategies outlined in Additional file 2: Appendix C into a step-by-step program with discrete components, mechanisms of delivery and timelines. Since the workplace partner already had an existing program, the intervention mapping team decided to conduct an internal gap analysis between suggested strategies and the current program. This gap analysis [18] was done internally because specific aspects of their current program are proprietary and therefore details were not provided. An example of the gap analysis using the employee and mental health is detailed in Table 6. Table 6 outlines what the workplace partner is currently doing and an action plan that describes the actions (strategies) the company contemplates to implement as a result of the intervention mapping process with potential timelines.

**Table 6:** Example of gap analysis and integration of practical strategies into an action plan for employee and mental health

Opportunity/Objectives	What Company is doing today	Action Plan due to Study	Discussion (yes/no/maybe) and timing
Inform employees at all levels about mental health issues Explain/de-mystify stigma around mental health Build confidence to be able to discuss mental health problems Demonstrate that leadership will be supportive of mental health issues Educate employees on when to seek help and find resources	Have communications about mental health and EAAP on the Source. Provide EAAP orientation sessions to managers that includes information about mental health Have an employee mental health presentation on the Source with a focus on destigmatizing mental health, an overview of mental health definitions and resources available to help them. Provide manager training sessions on “Managing Absence & Mental Health in the Workplace” to de-stigmatize mental health. Provide EAAP webinars on managing stress, dealing with anxiety, etc. throughout the year.	Incorporate focus groups as progressive element of “Managing Absence & Mental Health in the Workplace” workshops Monthly 30–60 min webinar discussion on absence and mental health facilitated by Disability expert with EPC support Forum would provide participants with opportunity to share ideas, challenges and successes. Objective is to create a network where people feel comfortable talking.	YES: Company Human Resources will be working with a Disability expert to implement one English and one French webinar per month Pilot will be implemented after March; in-person sessions with pilot webinars taking place in April/May 2013
	Provide EAAP magnets to employees through a desk drop so they will know who to seek help from when they need it.	Work with the Marketing and Communications team to incorporate themes around mental health into overall communications strategy Increase awareness, change behaviours and attitudes on mental health by using the Source to tell stories,	YES: Q4 2013: Will incorporate in October Healthy Workplace Month and December Mental Health (EAAP refresher) month YES: Q1 2013 (ongoing)
		Work with Marketing and Communications team to leverage existing national campaigns, events and themes, and distribute monthly themed articles to get people to have conversations about mental health issues (i.e., National non-Smoking Week in January, Eating Disorders Awareness Week in February)	

*EAAP* employee and advisor assistance program, *EPC* employment practice consultant

The intervention mapping process highlighted the need for improved communication and awareness in mental health across the organization. A main action item was to develop a comprehensive organization-wide communication strategy for mental health that includes regular employee focus groups and monthly webinar educational sessions. The goal of this communication strategy was to develop a network where employees feel comfortable sharing personal stories, challenges and successes. The action plan outlined many other specific strategies that would be part of the overall communication/awareness strategy and one that engages the entire organization. Another important action item was to integrate and harmonize the various mental health initiatives throughout the organization. The establishment of a Director of Mental Health and a Mental Health Website could facilitate this action plan. For managers, the action goals focus on training and communication. Currently manager training for mental health is voluntary and the plan is to make it mandatory for all managers.

There was a plan to include health and wellness objectives into the performance management process, highlighting the need for a cultural shift in the organization. Action plans for senior management also focused on improved communication using multi-media and multi-pronged approaches and incorporating strategies that demonstrate the “walk the talk” philosophy. From an organizational perspective there were plans to link business objectives with health objectives that would include specific outcome-reward incentive strategies to encourage participation and engagement. A specific example would be the development of a Wellness Ambassador Program where employees would be rewarded (with personal spending account dollars) and recognized for their participation.

Similar action plans directed at improving awareness, communication and participation/engagement were outlined for cardiovascular/diabetes/musculoskeletal/flu health conditions. These action plans can be implemented alongside the various health campaigns that are scheduled throughout the year, such as Back Health Week/National Spine Week (in June). Providing education and educational tools via the employee’s Group Benefit Plan Member website were also planned. There were action plans aimed at increasing participation in the annual comprehensive Health Assessment and using data from the Health Assessments to build awareness and provide feedback aimed at improving health behaviors. From an organizational perspective, there was a plan to use health metrics more effectively to benchmark the company’s performance in comparison to other similar organizations.

## Discussion

Intervention mapping was used to design a workplace health promotion and wellness program to improve presenteeism. The process highlighted strengths and weaknesses and gaps between strategies and interventions currently used by the workplace partner and those recommended by the intervention mapping team. This process provided a framework for our workplace partner to assess and to improve their current program. Main recommendations included strategies to improve employee engagement, awareness, communication, and sustainability of current initiatives. Improvement in these areas is essential for facilitating positive change in individual health behaviors. Strategies to realign the “message with the action” were also suggested. Although the organization says they value a healthy workplace, the current practices, policies and the actions of supervisor and senior management suggests otherwise. The BHCI results from the workplace survey suggested a perceived negative work culture was present, which can be associated with higher risk for presenteeism [20].

Mandatory training for supervisors and senior managers was recommended not just for mental health, but also for all-important health conditions identified in the needs assessment. High priority recommendations focused on strategies to shift the workplace culture towards one that places employee health and health promotion at par with company profits. This will require data on how this shift can positively impact the bottom line.

The impact of mental health conditions and especially depression in the workplace was highlighted as a main challenge and the highest priority for our workplace partner. Depression is one of the most debilitating diseases that can have significant effects on employees, co-workers, family members, and society [21–23]. Major depression is currently a leading cause of disability worldwide [23]. Musculoskeletal pain and disability, particularly repetitive strain and back and neck pain were seen by our workplace partner as a distant second in importance.

The key findings and recommendations of this study appear to be consistent with a systematic review evaluating the effectiveness of workplace health promotion programs at improving presenteeism [26]. This review concluded that successful programs appear to be those that offer organizational leadership, health risk screening, individually tailored programs and a supportive workplace culture. This study also found that potential risk factors contributing to presenteeism include being overweight, a poor diet, a lack of exercise, high stress, and poor relations with co-workers and management. Although Cancelliere et al. found preliminary evidence that some workplace health promotion programs can positively affect presenteeism, they caution that presenteeism literature is young and heterogeneous, with few high-quality studies and many uncertainties surrounding the measurement of presenteeism. There is currently no universally accepted method for measuring presenteeism [27, 28]

This study involved considerable effort in investigating the determinants of presenteeism. The main determinants of presenteeism investigated in previous studies resonated with those found in this study. These included stress-related factors at work, one’s personal health, and other individual factors [29]. Stress-related factors at work are due to the demands of the work environment, such as high work demands, work control and poor social climate [30–32]. These factors are modifiable and present an opportunity for change and were addressed by the intervention mapping process in this study. State of health not only leads to presenteeism but is also considered a mediator between stress-related factors at work and presenteeism [31, 32]. Individual factors, such as personality traits that impact work-life balance and interpersonal relationships have been significantly associated with stress related factors and presenteeism in the aging working population [29]. These factors may be more challenging to change.

It will be increasingly challenging for workplaces to maintain a healthy and productive workplace due to the increasing number of people affected with mental health and other chronic diseases, and an aging workforce that is more likely affected by these conditions [5]. The increasing awareness and rising costs associated with presenteeism have resulted in a significant increase in the demand for workplace health promotion programs [33]. There appears to be a growing realization that while containing health care costs and absenteeism have been important strategies for companies, greater gains may be realized by improving on-the-job productivity and investing in preventive and early intervention strategies [34–39]. Although workplaces have a strong stake in reducing presenteeism, the roles and responsibilities of stakeholders outside the workplace such as health care providers, governments and insurers in reducing presenteeism is less clear. Paid sick leave policies that provide employees with protected time off work with pay if they are sick is an example how government policy can reduce presenteeism. Access to paid sick leave is related to better medical treatment compliance, quicker recovery from illness and overall better health and well being for employees and their families [24]. Without paid sick leave employees do not seek necessary health care and are compelled to come to work sick and under preform [25]. Having an integrative approach with initiatives inside and outside the workplace may provide even greater gains.

### Appraisal of the intervention mapping approach and lessons learned

This is the first study that we are aware of that used intervention mapping to develop a workplace health promotion and wellness program aimed at reducing presenteeism. Intervention mapping has been traditionally used for designing community based programs or interventions. The workplace can be considered a community with various interactions and links with numerous important stakeholders and the local and broader environment. Consequently this methodology was highly applicable and was generally successful in meeting the study objectives. Unlike traditional community based programs that focus on a single condition like AIDS prevention or weight loss in children, reducing presenteeism may be more complex since it must consider a multitude of health conditions and their associated determinants. The main strength of the intervention mapping method was its comprehensive and systematic approach that provided a framework to address the complex and multifaceted aspects related to workplace health and presenteeism. In addition to using the best available evidence, the approach was participatory with input from important workplace stakeholders who provided practical insight on reducing presenteeism. Health promotion programs developed by employees are likely to get more buy-in and might be more successfully implemented [26]. A key strength was the focus on identifying modifiable individual behaviours and environmental factors, and collectively identifying practical solutions to overcome these barriers to reduce presenteeism. Mitigating high-risk individual behaviors and improving the workplace culture and environment is essential for sustainable reduction in presenteeism [38].

A central component in the intervention mapping approach was the core processes that compelled participants to think critically often in non-conventional ways. This fostered innovative solutions to tackle previously unsuccessful health improvement initiatives.

A significant drawback for using intervention mapping is that it is very time intensive. All intervention mapping steps including the needs assessment took three years to complete (see Timeline Fig. 2). This was primarily due to challenges in scheduling and accommodating the extensive number of study discussion groups and interview sessions required with the already high workplace demands experienced by participants.

Selecting a balanced representation of participants was challenging. There was low representation from lower levels jobs during Step 2–4 of the intervention mapping process. There was a bias towards more managers, supervisors, directors, and senior management participants. This potentially meant that the views, experiences, comments, and recommendations of the most vulnerable were not heard or addressed. Another weakness of the study was not engaging other workplaces in the intervention mapping process with the opportunity to learn and share practices. However, this would require the sharing of proprietary information and given the competitive nature of the industry, this was not possible. A potential limitation to the needs assessment was the use of the in-house survey data that had low participation rates and had only summary scores and not the raw data. The representativeness of this data to the overall work population was unknown. Another potential weakness of the study was the use of a single workplace that may limit the generalizibility of the results. Moreover, this study provided a Canadian workplace perspective that was embedded within a universal government-funded health care system and therefore barriers and solutions to implementing workplace health promotion and wellness programs may differ based on setting and health care policies and practices. However, it is likely that many workplaces around the world face similar challenges to those expressed by our workplace partner; therefore the recommendations outlined in this intervention mapping process may be useful, although it is likely they need to be tailored to the individual workplace. Limited evidence from the scientific literature on effective interventions for reducing presenteeism resulted in recommendations based heavily on the experiences and opinions of participants and therefore lacked scientific rigor. The ability for these recommendations to improve outcomes and reduce presenteeism is uncertain.

Future attempts at using intervention mapping to design a workplace health and wellness program to reduce presenteeism should consider the following: an independent needs assessment that collects primary data or at the very least access to all raw data from in-house health assessments and administrative data; Separate Step 2–4 discussion group sessions with similar job titles in order for participants to share information more freely; Strong representation among high-risk employees and top decision makers throughout the process; tighter timelines for improved efficiency and continuity of the process.; An external facilitator such as an independent researcher who can develop trust and impartiality among all stakeholders and employees.; finally, an evaluation of the designed workplace health promotion program using predetermined validated metrics.

## Conclusions

We used intervention mapping and collaborated with a workplace partner with the goal to reduce presenteeism by improving their current health promotion and wellness program. The intervention mapping process explicitly outlined strengths and weakness of the current program, delineated all-important stakeholders and prioritized important health conditions that impact presenteeism. A detailed description of the necessary change and learned objectives that are required for each stakeholder for each priority health condition provided the framework to develop new and improved current strategies to accomplish these objectives. The final product was a document that outlined specific action plans to be incorporated into the current program. However, the main benefit of this study was the intervention mapping process that brought together a broad spectrum of stakeholders who worked together to critically appraise (self assessment) the current program and to develop potential solutions to improve the program. The process compelled participants to think critically and collaboratively and often-in non traditional ways. It is this process that leads to innovation, and it is innovation that will lead to excellence in this field.

## Appendix A

**Table 7:** Step 2 Performance objectives for each stakeholder and health condition

Mental Health	Cardiovascular/Diabetes	MSK	Cancer	Flu
a) employee - de-stigmatize MH - look at it differently - open communication - know when to seek help - seek out positive relationships with peers/leadership - use available resources - minimize isolation participate in social networks - avoid gossip or other negative behaviours - compliance re: medication/counselling/support - exercise minimum 30 min per day - get adequate sleep - proper diet/nutrition - avoid tobacco - minimize alcohol - engage in stress/time management work/life balance - discuss with manager/supervisor any limitations	a) employee - do regular exercise (at least 20 min of walking 5 out 7 days per week) - test for high cholesterol or high lipids in the blood - check blood pressure (identify if they are at risk) - eat properly - reduce fat intake, increase fibre, increase greens, - stress management - avoid tobacco use - reduce refined sugars/alcohol - be aware of the signs and symptoms and when they do occur do something about it - monitor diet, reduced processed foods and eating out - check BMI - be aware of the drugs that you are taking (drug interactions can impact cardiovascular health) - weight management - understanding family history - sleep hygiene - work-life balance - seeing family doctor for regular check-up - medication compliance/appropriate use of medication - discuss with manager/supervisor any limitations - monitor BP and blood glucose regularly	a) employee - regular exercise (30 min/day) - seek advice on appropriate exercise/self management - maintain adequate weight - proper diet - lifestyle, walking - sit more ergonomically-appropriate work posture (reduce physical strains of sitting) - if at risk for arthritis - need to have it checked out by health care person - aware of links between musculoskeletal health and depression - if they have physical limitations, need to communicate with their manager - knowing when to seek help if they have physical pain - take the appropriate medication - reduce physical work hazards - reduce stress-learn stress management skills - yoga - develop positive relationships with co-workers/manager/supervisors - adapt/accommodate worksite - take regular breaks	a) employee - do regular exercise (at least 20 min of walking 5 out 7 days per week) - learn self breast exam - learn about prevention (colon/breast/skin/prostate/lung) - know about family history and risk - eat properly - reduce fat intake, increase fibre, increase greens, reduce alcohol use - stress management - avoid tobacco use - sun protection - be aware of the signs and symptoms and when they do occur do something about it - monitor diet, reduced processed foods and eating out - weight management - adequate/sleep hygiene - work-life balance - seeing family doctor for regular check-up/screening - take recommended medications and seek appropriate care compliance/appropriate use of medication - discuss with manager/supervisor any limitations - seek support from manager/co-workers/home/community - learn to build positive relationships - learn/avoid known carcinogens - awareness of available resources	a) employee - get the flu shot; understand the risks and benefits of the flu shot -decide whether the flu shot is right for them - eat properly - exercise - get enough sleep - wash hands - know how to adequately wash hands work/home - good sanitary practices at work and at home - limit exposure to others who are sick - if sick, limit exposure (stay home if they are sick) - cough/sneeze into elbow not hands - use hand sanitizers regularly
b) manager/supervisor - adequate training/training to develop positive relationships with employees - avoid stigma/better understanding - discuss issues with employee/foster open communication - know what resources are available - employ emotional intelligence - know when to seek help - follow processes - learn to identify behavioural issues - demonstrate flexibility and compassion - help enable employee to get better - mentorship for prevention - encourage employees to participate in wellness - recognize each employees' 'normal' - Provide positive recognition of employees - minimize ambiguity in employee roles	b) manager/supervisor - role model (eating properly, exercising, going out for a walk at lunch time, work-life balance, adequate weight) - accountability - awareness of signs and symptoms of employees (e.g. arm pain) - education/training re signs of cardiovascular disease/diabetes - develop positive relationships with employees/subordinates - can lead to open communication, reduced stress - accommodation/flexibility on the part of managers and supervisors - better understanding and being active about it - providing modifications, resources - awareness of resources available to employees (e.g. wellness programs, fitness programs) - show compassion and concern	b) manager/supervisor - be educated about musculo-skeletal disorders - observe people sitting at their desk - identifying and suggesting an ergonomic assessment - positive relationships - role models around exercise/diet/positive attitudes/ - build and maintain positive supportive relationship - monitor physical/stress demands - awareness of signs and symptoms of MSK - suggest/accommodate to workers capabilities/tolerance	b) manager/supervisor - modify work to suit limitations - provide support/empathy - develop positive relationships - aware of available resource to employees - role model for healthy living - monitor work stress demands - positive communication skills - encourage employees to engage in wellness programs - recognize early signs…weight loss, change in behaviours, malaise, excessive time off work etc. - show compassion and concern	b) manager/supervisor - support staff if they need to stay home if they are sick - manage work, clients - understand the HR absence policies and processes and share with the team - make sure everyone is aware of the steps they need to take if they are sick (who to call, how often to check in) - be a role model in terms of doing the necessary healthy behaviours (eating well, exercising, washing hands) - instructing employees for where they can find resources that explain the benefits and risks flu shot (education)
c) co-workers - de-stigmatize mental health - be supportive - Work together common goals and objectives - be observant - look after each other - respect confidentiality - help create a positive environment - don't turn a blind eye - reach out to manager when needed - learn about mental illness - show compassion and concern	c) co-workers - awareness of early signs, identify amongst co-workers (fostering knowledge) - support, positive relationships - show compassion and concern - Work together common goals and objectives - positive role model (engage in healthy behaviours and wellness programs)	c) co-workers - going for walks - adequate weight - support, positive relationships - show compassion and concern - Work together common goals and objectives - positive healthy role models(eating, exercise, weight, attitudes, relationships, use of proper ergonomic, work-life balance) - awareness of signs and symptoms of MSK	c) co-workers - positive healthy role models (eating, exercise, weight, attitudes, relationships, work-life balance) - show compassion, support and concern	c) co-workers - be a positive role model - get the flu shot; understand the risks and benefits of the flu shot - decide whether the flu shot is right for them - eat properly - exercise - get enough sleep - wash hands - know how to adequately wash hands work/home - good sanitary practices at work and at home - limit exposure to others who are sick - if sick, limit exposure (stay home if they are sick) - cough/sneeze into elbow not hands - use hand sanitizers regularly
d) directors/senior management - leadership to prioritize mental health - invest time and budget - walk the talk - lead by example - participate in training - learn to identify behavioural issues - demonstrate flexibility and compassion - enable employee to get better - mentorship for prevention - encourage employees to participate in wellness - training to develop positive relationships with employees - foster open communication - recognize each employees' 'normal' - Provide positive recognition of employees	d) directors/senior management - role models, walk the talk (diet, exercise, lifestyle, work-life balance) lead by example - have training with regards to Cardiovascular Disease - demonstrating accommodation, flexibility - expressing to managers - compassion, understanding, proactive nature of direction - positive relationships - allow for budgeting for programs related to cardiovascular/diabetes disease (providing time and resources to educate)	d) directors/senior management - positive role model - resources around ergonomics - training in musculo-skeletal health - money, resources, time - walk the walk - make sure they are sitting properly with the correct equipment	d) directors/senior management - role models, walk the talk (diet, exercise, lifestyle, work-life balance) lead by example - expressing to managers - compassion, understanding, be proactive - allow for budgeting for programs related to cancer (providing time and resources to educate) - training to develop positive relationships with employees - foster open communication - encourage flexibility among mangers/supervisors	d) directors/senior management - education and awareness about prevention - providing information - e.g. biometric screening clinics, flu prevention - make sure everyone knows the plan- business continuity (e.g. flu pandemic) - having a plan in place for what the organization should do - awareness of daycare programs
e) organization - benchmarking - comparing organization to others re mental health - establish mission and philosophy around health of employees - communicate expectations and procedures to managers and employees - set the tone for the culture - enforce policies and procedures - align policies and procedures to philosophy - know the standards from regulatory bodies (e.g. mental health commission of Canada) - provide resources for training managers/supervisors/employees - invest in social capital	e) organization - benchmarking - how well are we doing in comparison with other companies with regards to cardiovascular/diabetic claims? - mission statement, main message around health in general - communication around expectations re: health/wellness-make health/wellness the norm - culture - how important is the well-being of our employees - policies and procedures related to wellness and prevention - instill/facilitate a health/safety culture - invest in social capital	e) organization - benchmarking- how well are we doing in comparison with other companies with regards to MSK claims? - instill/facilitate a health/safety culture - policies and procedures related to wellness and prevention - communication around expectations re: health/safety/wellness-make health/wellness the norm - provide resources for training managers/supervisors/ employees - invest in social capital	e) organization - benchmarking - how well are we doing in comparison with other companies with regards to cancer related claims? - mission statement, main message around health in general - instill/facilitate a health/safety culture - policies and procedures related to wellness and prevention - communication around expectations re: health/wellness-make health/wellness the norm - invest in social capital	e) organization - having the right practices and policies in place to support short-term absences due to illness (e.g. offering personal illness days to employees, ensuring this benefit aligns with employees' needs) - resources - provide the flu shot clinic for those who are interested in getting the shot - business continuity (e.g. flu pandemic) - having a plan in place for what the organization should do - invest in social capital
f) family/partner/spouse/community - supportive environment - understanding of mental health and reduced stigmatization - understanding of the impact of mental health on the individual - discuss openly and honestly with the person having mental health problems - recommendation to seek help, not be afraid to seek help - positive influence - foster awareness about available support and programs (community perspective) - taking ownership for investigating what programs and support are available (partner, family perspective) - have resources available for people who need help when they need it (community) - targeting at a young age- bringing awareness and providing resources to all demographics (identification of the signs)	f) family/partner/spouse community - being aware of early signs of Cardiovascular Disease - encourage family members to go to the doctor for check-ups - encourage healthy eating, diet, nutrition at home - encourage exercise, more walking - proper use of medication - being aware of and providing support for proper use of medication - education on signs and symptoms/prevention	f) family/partner/spouse community - encourage physical activity, healthy weight - education around how to lift properly - model for healthy living - education on proper ergonomic at home - understanding role of self-management in chronic MSK disorders - understand the relationship between chronic MSK and depression/anxiety - make regular exercise proper ergonomics and healthy eating the norm - provide encouragement and support for ongoing treatment - awareness of the role and potential negative consequences of narcotic use for pain	f) family/partner/spouse community - positive healthy role models (eating, exercise, weight, attitudes, relationships, work-life balance) - make healthy lifestyle the norm - provide emotional support and encouragement - understand the impact cancer can have on the individual - support/assistance in treatment…chemotherapy/radiation/medication treatment and be aware of side-effects - be aware of community/work resources/support groups to assist in management - awareness of the role and potential consequences of narcotic use for pain - aware of Canadian Cancer Society resources	f) family/partner/spouse community same as above (employee and co-worker) - providing support to people that want to take the shot - provide information, education - provide support for the people that need to stay home - provide opportunity to citizens to have the shot - hygiene education in terms of prevention
g) health care practitioners - ability to screen for individuals at high risk and provide appropriate interventions (e.g. referral, medication, counselling, etc.) - stay up-to-date and current with regards to mental health trends in Canada (number of Canadians impacted - stimulate dialogue about prevalence, reducing stigma/treatment, resources) - explaining to employees why information is provided to different stakeholders so that appropriate case management and adjudication happens - having more dialogue with the employee, providing teaching and information about why it is important to provide information up front - holistic approach (combination of medications, therapy, etc.) commitment to keep working at a treatment plan - follow-up - is the treatment plan working? what adjustments are needed? ongoing case management at the doctor-patient level	g) health care practitioners - provide screening for high risk individuals - provide appropriate interventions - provide educational resources for people to look for in terms of Cardiovascular disease and risks - counseling in terms of exercise and diet, provide appropriate resources - regular follow-ups to see if risk factors are under control - balancing pharmacological with non-pharmacological interventions	g) health care practitioners - identification, follow-up, assessment - aware of ergonomic issues, educate patients on proper ergonomics/prevention - recommend/refer for appropriate treatment/management - encourage to stay active - communicate with workplace for work modification - positive role model	g) health care practitioners - appropriate screening and management - communication with workplace on work modification - provide emotional support and educate family members - provide patient education on prevention and self monitoring - educate on community resources - positive role model - educate on self monitoring	g) health care practitioners - education, especially for high-risk people - providing the flu shot for those that want it - education in the area of prevention - dispensing medication that is needed - be clear to the employee on restrictions and limitations (e.g. when they can return to the office) - communication via employee to the workplace - between health care provider's recommendations to the workplace, via the employee - provide people with information regarding alternatives to medicine - EAP - provide education, daycare issues, family care (consequences, what to do)
h) high risk employees - same as employees - read emails and information about health promotion - make people more aware and find ways for them to become more aware about available programs, become more proactive, rather than reactive - take accountability for themselves - accept that they are responsible for their own behaviour (other stakeholders are not responsible to ensure that people take appropriate action) - leaders also need to create capacity for people to participate	h) high risk employees - same as employees - responsibility and accountability - education (same as mental health)	h) high risk employees - same as employees - ask for ergonomic assessment - use proper postures at work	h) high risk employees - same as employees	h) high risk employees - same as employee

*EAP* employee assistance program, *MH* mental health, *BMI* body mass index, *BP* blood pressure, *MSK* musculoskeletal, *HR*+ human resources

## Additional files

Additional file 1: Appendix B. Step 2 Matrix A-E. Individual and environmental determinants of listed performance objectives for each stakeholder and priority health conditions and the required learn and change objectives. (DOCX 236 kb)
Additional file 2: Appendix C.Step 3. Translate learn and change objectives into to practical strategies. Mental Health and General. (DOCX 78 kb)

## Abbreviations

BHCI: Business health culture index
WPAIQ: Work productivity and activity impairment questionnaire
