# Supplementary material for: Healthy and productive workers: using intervention mapping to design a workplace health promotion and wellness program to improve presenteeism
Source: BMC Public Health. 2016 Nov 25;16:1190. doi: 10.1186/s12889-016-3843-x (PMC5123329; doi:10.1186/s12889-016-3843-x)
Supplement: Additional file 1: — Appendix B. Step 2 Matrix A-E. Individual and environmental determinants of listed performance objectives for each stakeholder and priority health conditions and the required learn and change objectives. (DOCX 236 kb) [file 12889_2016_3843_MOESM1_ESM.docx]

**Additional file 1: Appendix B. Step2 Matrix A-E.** Individual and environmental determinants of listed performance objectives for each stakeholder and priority health conditions and the required learn and change objectives

| **Mental Health – Employee Individual Determinants** | | | | |
| --- | --- | --- | --- | --- |
| **Performance**  **Objectives** | **Knowledge, capability or skill** | **Attitudes, beliefs and values** | **Expectations** | **Self efficacy** |
| De-stigmatize mental health  look at it differently  open communication | Describe that it’s okay to talk about depression/anxiety/ mental health issues  Inform employees at all levels about mental health issues | Explaining/de-mystifying stigma around mental health | To demonstrate that by talking about my mental health problems, that I will get empathy or understanding from my organization/peers/etc. | To build confidence to be able to discuss mental health problems |
| Know when to seek help | Describe or explain what help is available  Explain that there are resources available that will tell you how to seek help (who, where to go) | Explain that by seeking help it will be dealt with in a confidential way  Explain or describe that mental health can be effectively treated | Demonstrate that people that seek help can be helped | Provide the resources to foster the self-confidence for people to know when and where to seek help when they need it |
| seek out positive relationships among peers and leadership | Ensure leaders know what the organization expects from them  Inform employees about the role leaders play  Encourage employees to discuss mental health issues  Explain the role of positive relationships in mental health  Inform that leadership has been trained in mental health |  | Demonstrate that leadership will be supportive of mental health issues | Improve confidence in their willingness to seek leadership advice |

**Matrix A**

**Mental Health -Employee**

| **Mental Health – Employee Individual Determinants** | | | | |
| --- | --- | --- | --- | --- |
| **Performance**  **Objectives** | **Knowledge, capability or skill** | **Attitudes, beliefs and values** | **Expectations** | **Self efficacy** |
| use available resources | Describe where they can find available resources when they are having a mental health issue | Explain that by using available resources it will be dealt with in a confidential way |  | Improve confidence in seeking out available resources |
| minimize isolation  participate in social networks | Explain why isolation can impact mental health / why participating in social activities reduces risk of mental health, consequences of mental health issues  Explain how to participate in social networks | Explain that social support is important in addressing a mental health issue |  | Improve confidence in participating in social networks |
| avoid gossip or other negative behaviours | Explain how negative behaviours or gossip impact mental health | Change attitudes around the impact of gossip at the workplace | Create an atmosphere where negative behaviour is not condoned | Build confidence around how to stop gossip or other negative behaviours |
| Compliance re medication / counselling/ support | Describe the benefits of seeking support, medication compliance (how to, consequences of not following) | Explain that taking as recommended will impact success  Change belief around the stigma of taking medication | Describe that it is okay to seek support (also applies to attitudes) | Build confidence to seek support  Build confidence to manage your own compliance |

| **Mental Health – Employee Individual Determinants** | | | | |
| --- | --- | --- | --- | --- |
| **Performance**  **Objectives** | **Knowledge, capability or skill** | **Attitudes, beliefs and values** | **Expectations** | **Self efficacy** |
| Exercise minimum 30 minutes per day (150 min/week) | Explain how exercise is beneficial to mental health – links on source to articles for example  Demonstrate how you can find 30 minutes per day to exercise  Explain how exercise can improve your work performance, increase efficiency | Explain that it is possible to find 30 min per day to exercise – provide tips and examples | Build positive expectations around the ability to exercise and the benefits that can be realized. | Build confidence to find 30 minutes during the day to exercise and that it will not impact work performance |
| Get adequate sleep | Explain how one can achieve adequate sleep (7 hours) and where to find the resources to assist them in getting better sleep  Explain the difference between quantity and quality of sleep and how needs might differ between people | Explain that it is possible to obtain adequate sleep | Build positive expectations around the ability to get good sleep | Build confidence in ability to utilize strategies to get better sleep |
| Proper diet/nutrition | Explain how one can achieve adequate nutrition (low fat, high fibre, low sugar, calories) and where to find the resources to assist them in getting better nutrition | Explain that it is possible to eat properly (provide tips and links)  Explain how proper diet/nutrition can positively impact mental health | Build positive expectations around the ability to have nutritious meals | Build confidence to seek/make nutritious meals  Provide tips on how to eat healthy |

| **Mental Health – Employee Individual Determinants** | | | | |
| --- | --- | --- | --- | --- |
| **Performance**  **Objectives** | **Knowledge, capability or skill** | **Attitudes, beliefs and values** | **Expectations** | **Self efficacy** |
| Avoid tobacco  Minimize alcohol | Describe how these can impact mental health  Describe resources to be able to stop smoking, where you need to go seek help | Demonstrate that it is okay to seek help | Persuade that it is possible stop smoking or reduce alcohol | Build confidence that you can make changes to these issues |
| Engage in stress/time management  work/life balance | Learn how to better manage time and stress  Describe the available resources to help work/life balance | Show that it is possible to have work/life balance  Demonstrate that it is OK to seek help for stress management | Persuade that it is possible to reduce stress  Demonstrate that it is okay to create work/life balance in a way that works for you | Build confidence to seek help when work/life stress is totally out of balance |

| **Mental Health – Employee External Determinants** | | | | | |
| --- | --- | --- | --- | --- | --- |
| Performance  Objectives | **Norms**  **and policies** | **Social support** | **Reinforcement** | **Resources** | **Organizational climate** |
| De-stigmatize mental health  look at it differently  open communication | Explain the anti-harassment and accommodation policies and standards  Senior leaders share their experience in working with mental health issues; senior leaders talk openly | De-stigmatization training for managers  Add de-stigmatization training for employees | Demonstrate and praise individuals who discuss their mental health issues | De-stigmatization training for managers  Add de-stigmatization training for employees | Demonstrate that it’s okay for employee to discuss openly the issues around mental health as it impacts their function at work;  Demonstrate to employees that the organization cares and will be compassionate towards the issues and managers will be understanding  Senior leaders share their experience in working with mental health issues; |
| know when to seek help | Promote/reinforce the importance of manager training | Emphasize that if employees seek help they will be supported by their team/manager | Incorporate mental health training into new manager training  Include applicable information in new employee manual/training | Enhance visibility of resources available to employees | Promote an organizational culture where it is okay to seek help |

| **Mental Health – Employee External Determinants** | | | | | |
| --- | --- | --- | --- | --- | --- |
| Performance  Objectives | **Norms**  **and policies** | **Social support** | **Reinforcement** | **Resources** | **Organizational climate** |
| Seek out positive relationships among peers and leadership | Demonstrate how teams who have positive relationships impact the work environment (e.g. socializing with teams; creating opportunities to know each other better) | Demonstrate within the teams that positive relationships are encouraged and well accepted while negative talk/gossip is discouraged within the teams | Recognize/praise positive relationships that are developed by employees |  | Demonstrate how senior leaders and their relationships impact the organization positively |

| use available resources | Demonstrate how team leaders can provide information/promote resources to team members (e.g. in team meetings) |  | Demonstrate using data or graphs the utilization of the resources by employees  Host a fair where vendors can provide education and information on services available | Demonstrate that resources are readily accessible; enhance communication and visibility around the resources  Enhance visibility through communicating in common areas (e.g. cafeterias) | Encourage the use of the available resources when needed  Attach the company brand to philanthropy to support the area of mental health  Demonstrate to employees what we are doing in the area of philanthropy and mental health (e.g. Adolescent Mental Health – Dalhousie) |
| --- | --- | --- | --- | --- | --- |

| **Mental Health – Employee External Determinants** | | | | | |
| --- | --- | --- | --- | --- | --- |
| **Performance**  **Objectives** | **Norms**  **and policies** | **Social support** | **Reinforcement** | **Resources** | **Organizational climate** |
| minimize isolation participate in social networks | encourage face to face communication ; being in the moment during meetings | demonstrate by team leaders the norms-expectation | team leaders demonstrate the norms and encourage others to participate | train managers how to engage employees and avoid isolation | Describe a culture that encourages employees to get involved; feel safe about participating |
| avoid gossip or other negative behaviours | managers as an example - encourage managers to be role models with respect to avoiding gossip and neg. behaviours | Within teams and working groups support and promote positive behaviours | managers reinforce positive behaviours | Include in HR documents/ new employee documentation  Include in value proposition | managers expected to take action on negative behaviours/gossip  culture describes  low tolerance for employees to engage in this behaviour  explicitly talk about these behaviours within our employee value proposition |
| Compliance re medication / counselling/ support | describe that the norm for mental health issues is to receive counseling | provide through benefits program to reduce stigmatization around counseling | educate employees on the importance of compliance; reinforce that the organization is here to support employees  Provide feedback using HRA | Use flex benefits  and microsite to educate  Encourage use of EAP communications  Include info on Source  Provide information on the importance of medication adherence |  |
| Exercise minimum 30 minutes per day 5 days per week | mentorship by management; role models for exercise and get away from their desk on lunch | Provide a forum for individuals looking for social support to promote physical activity (i.e. discussions for people to look for group fitness activities) | Use verbal reinforcement and reward positive behaviour  sharing of best practices (e.g. collaboration tool or forum to share what employees/managers are doing) | Use videos  (e.g. 23 1/2 hours)  Wellness programs such as health challenges  Reinforce programs such as Weight Watchers at Work, fitness reimbursements, Wellness Account, on-site gyms | senior leaders demonstrate participating in national wellness day and other wellness programs offered |

| **Mental Health – Employee External Determinants** | | | | | |
| --- | --- | --- | --- | --- | --- |
| Performance  Objectives | **Norms**  **and policies** | **Social support** | **Reinforcement** | **Resources** | **Organizational climate** |
| Adequate sleep | Provide guidelines on avoid sending emails late at night too often, respectful of getting sleep  Describe the recommended sleep requirement (guidelines) |  | Provide feedback using HRA assessment | education around the impact of sleep deprivation and how to ensure adequate sleep  EAP program; sleep webinars as part of wellness program; HRA sleep data for analysis to see if it's going in the right direction over time | emphasize CEO messaging around being a high performance culture and the type of climate we're striving for and how we're going to get there; ensure employees know that a positive culture is important |
| Proper diet/nutrition | show role modeling; walking the walk; healthy snacks and meals for meetings; where cafeterias exist, ensuring healthy options  discuss about the community norms shifting toward healthy food/drink choices  Provide more option in drinks like juices rather than coffee or pop | Demonstrate peer to peer support around positive eating habits;  collaboration spaces or forums to share success stories; profile leaders favorite recipes | Demonstrate positive reinforcement by managers on healthy choices by employees | healthy cafeteria food; information on the wellness -part of the Source website e.g. healthy recipes | Senior management demonstrates the importance of proper nutrition and healthy lifestyle and how this is important for a high performance culture |
| Avoid tobacco  Minimize alcohol | Describe that the norm (trends) is to avoid excessive Alcohol and avoid tobacco  Norms around alcohol during socials/limiting use/  Describe policy around allowable expenses for alcohol | Role modeling among peers and provide to help/support for those wanting to quit smoking | Reinforcing smoking policies  Enforce policy smoking around building (second hand smoke) | Demonstrate how EAP can be used to assist employees  Leverage using the Source with other community programs/initiate | An organizational climate that values a positive healthy lifestyle and demonstrates this through the actions of senior management |

| **Mental Health – Employee External Determinants** | | | | | |
| --- | --- | --- | --- | --- | --- |
| Performance  Objectives | **Norms**  **and policies** | **Social support** | **Reinforcement** | **Resources** | **Organizational climate** |
| Engage in stress/time management  work/life balance | Describe flex time schedule to enable employees to accommodate home situation and have time flexibility  Encourage telecommuting and use of flex hours  Encourage discussions between management and employees to regularly check in on stress/time management and work/life balance | Describe how peers encourage and support others who attempt to achieve work-life balance | Directors/ leaders demonstrate work life balance  On The Source have stories/testimonials that may have an effect to model other to achieve the same | Health and wellness programs that focus on stress mgmt., work life balance, time mgmt.  Provide education and opportunities to learn relaxation and stress management eg, lunch and learn  Promote the EAP as resource to get help | Describe a climate by which employee attempt to achieve work-life balance |

**Mental Health – Manager/Supervisor**

| **Mental Health – Manager/Supervisor Individual Determinants** | | | | |
| --- | --- | --- | --- | --- |
| **Performance**  **Objectives** | **Knowledge, capability**  **or skill** | **Attitudes, beliefs and values** | **Expectations** | **Self efficacy** |
| Get adequate training - have better understanding of MH issues  -learn to identify behavioural issues | Acquire the knowledge and skills  Participate in manager training on identifying mental health issues and how to deal with it  Encourage discussions around what is mental health vs. mental illness | Eliminate negative attitudes toward mental health  Understand that it is OK to talk about Mental illness and seek help | Expectations that managers will participate in training and deal appropriate with employees with mental health issues | Build confidence for managers to identify and take appropriate steps when presented with mental health issues |
| Avoid stigma  employ emotional intelligence  discuss issues with employee | Acquire knowledge and training  Demonstrate compassion and concern | Building awareness and demystify mental health issues | Expectations that managers will act in a manner that does not stigmatize  Expected to have skill and knowledge to appropriately handle mental health issues of employees | Confidence to speak to the issues and talk to employees within their boundaries  Demonstrates confidence to appropriately discuss/identify/act on issues related to mental health |
| -know what resources are available  -follow processes  -know when to seek help  - minimize ambiguity in employee roles | Be aware of available resources and what procedures to follow  Understand role and role of employee in mental health | Encourage attitude that manager and supervisors have an important role in mental health | Awareness of processes, role and resources | Demonstrate the confidence to follow processes, when to ask for help and knowledge of available resources |

| **Mental Health – Manager/Supervisor Individual Determinants** | | | | |
| --- | --- | --- | --- | --- |
| **Performance**  **Objectives** | **Knowledge, capability or skill** | **Attitudes, beliefs and values** | **Expectations** | **Self efficacy** |
| - demonstrate flexibility and compassion  - foster open communication  - recognize each employees' 'normal' | Participate in training and increase knowledge and skill development | Demonstrate caring and compassion and openness | Encourage participation in the wellness programs | Demonstrates confidence to provide praise and recognition |
| -mentorship for prevention  -encourage employees to participate in wellness |  | Express belief that participation is important in prevention | Encourage participation in the wellness programs |  |
| - help enable employee to get better | Acquire skills and knowledge to enable employees to get help they need | Develop the attitude that you can make a difference | Demonstrate willingness  To help | Demonstrate confidence to provide resources to employees to get better (knowing what to provide, when to provide it, etc.) |
| Provide positive recognition of employees | Communicate ways to recognize employees  Obtain training on how to praise and recognise  Profile on intranet recognition | Understand the importance of recognition | Provide positive recognition | Demonstrates confidence to provide praise and recognition |

| **Mental Health – Manager/Supervisor External Determinants** | | | | | |
| --- | --- | --- | --- | --- | --- |
| Performance  Objectives | **Norms**  **and policies** | **Social support** | **Reinforcement** | **Resources** | **Organizational climate** |
| -Get adequate training  -have better understanding of MH issues  -learn to identify behavioural issues | Inform that it is the norm (policy) that all managers/supervisors obtain training on best practices  Comparison with other organizations (feedback) | Other manager provide support on how to manage MH issues  Encourage support among managers | Senior management recognizes those who have helped others with MH. The Source highlights role  Opportunity to share experiences with other managers | Management provides sufficient resources for education and training  Have refresher training sessions | Demonstrated climate of understanding and compassion and open dialogue among managers/supervisors |
| -avoid stigma  -employ emotional intelligence  -discuss issues with employee | Inform that it is the norm (policy) that all managers/supervisors obtain training | Encourage open communication and sharing among managers | Senior management recognizes those who have helped others with MH. The Source highlights role | Management provides sufficient resources for education and training | Senior leaders share their experience in working with mental health issues |
| -know what resources are available  -follow processes  -know when to seek help  - minimize ambiguity in employee roles | It is the norm that managers are aware of the resources/processes | Shows high support among managers for importance of awareness of resource/processes | Senior management/ company publications/ wellness website/training manuals, The Source all  Provide information on available resources for MH for managers | Enhance visibility of resources available to managers/supervisors  Have a go to person for advise/guidance |  |
| demonstrate flexibility and compassion  - foster open communication  - recognize each employees' 'normal' | Inform that it is the norm (policy) that all managers/supervisors receive training in MH and understand role | Shows high support among managers for importance of open communication/ compassion and flexibility | Develop policies to recognize managers who excel in role  Senior management provides positive feedback on their performance |  | Demonstrated climate of understanding and compassion and open dialogue among managers/supervisors and employees |

| **Mental Health – Manager/Supervisor External Determinants** | | | | | |
| --- | --- | --- | --- | --- | --- |
| Performance  Objectives | **Norms**  **and policies** | **Social support** | **Reinforcement** | **Resources** | **Organizational climate** |
| -mentorship for prevention  --encourage employees to participate in wellness | It is the norm that managers act as role models in MH and participate in wellness initiatives and encourage employees to participate | Manager encourage other managers to be role models and participate in wellness initiatives | Senior management recognized managers who participate in wellness programs and act as role models in MH prevention |  | Construct a climate where everyone wants to participate in MH wellness initiatives |
| - help enable employee to get better | Define the norm/policy that managers be trained how to enable employees to get better | Managers assist each other on how to enable employees |  | Managers receive the necessary training on how to enable employees | Construct a climate where managers want to help enable employees to get better |
| Provide positive recognition of employees | Describe it is the norm to provide positive recognition to employees |  | Reinforcement by senior leadership of managers/supervisors who provide positive recognition of employees | Provide necessary training on how to provide positive recognition of employees | Senior management demonstrate positive recognition of managers |

**Mental Health – Co-worker**

| Mental Health – Co-worker Individual Determinants | | | | |
| --- | --- | --- | --- | --- |
| Performance  Objectives | **Knowledge, capability**  **or skill** | **Attitudes, beliefs and values** | **Expectations** | **Self efficacy** |
| de-stigmatize mental health | Describe that it’s okay to talk about depression/anxiety/ mental health issues  Inform employees at all levels about mental health issues | Explaining/de-mystifying stigma around mental health | demonstrate that by talking about mental health problems, co-workers will provide sympathy and understanding | build confidence to be able to discuss mental health problems |
| be supportive - Work together common goals and objectives-show compassion and concern | Inform co-workers on importance of being supportive and showing compassion | Develop a supportive and caring attitude for MH | All co-workers work together and show concern and compassion | Demonstrates confidence in showing compassion and concern |
| help create a positive environment | Develop skills to develop and maintain a positive environment | Show how to act into a new way of thinking. Demonstrate that a positive environment is a fun place to work |  | Demonstrate that employees have the ability to provide suggestions in creating a positive environment |
| learn about mental illness | Provide accessible information about MH to all employees | Demonstrate the belief that knowing about MH is important | It is expected that all co-worker learn about MH | Demonstrate confidence in learning about MH |

| Mental Health – Co-worker Individual Determinants | | | | |
| --- | --- | --- | --- | --- |
| Performance  Objectives | **Knowledge, capability or skill** | **Attitudes, beliefs and values** | **Expectations** | **Self efficacy** |
| reach out to manager when needed | Understand the importance to reach out to managers when you suspect MH issues among co-workers | Describe the attitude that we all have a role in MH identification and prevention | It is the norm to reach out to managers when suspect MH issues in co-worker | Demonstrate the confidence to reach out to manager when suspect MH issue among co-worker |
| respect confidentiality | Obtain the knowledge around importance of maintaining confidentiality | Demonstrate high level of respect for confidentiality around MH issues of co-workers | It is the norm not to discuss the MH issues of co-workers with other persons |  |

| Mental Health – Co-worker  **External Determinants** | | | | | |
| --- | --- | --- | --- | --- | --- |
| Performance  Objectives | **Norms**  **and policies** | **Social support** | **Reinforcement** | **Resources** | **Organizational climate** |
| de-stigmatize mental health | Explain the anti-harassment and accommodation policies and standards  Senior leaders share their experience in working with mental health issues; senior leaders talk openly | De-stigmatization training for managers  Add de-stigmatization training for employees | Demonstrate and praise individuals who discuss their mental health issues |  | Demonstrate that it’s okay for employee to discuss openly the issues around mental health as it impacts their function at work;  Demonstrate to employees that the organization cares and will be compassionate towards the issues and managers will be understanding  Senior leaders share their experience in working with mental health issues; |
| be supportive - Work together common goals and objectives-show compassion and concern | Make it the norm that co-worker are supportive and show compassion and concern for other co-workers with MH | Describe an environment where co-workers support each other | Co-workers are praised for supporting and showing compassion | The Source provides examples and re-enforces a supportive environment. Wellness programs encourage a supportive workplace | Organizational climate is such that a supportive and compassionate workplace is the norm |
| help create a positive environment/ relationship | Demonstrate how teams who have positive relationships impact the work environment (e.g. socializing with teams; creating opportunities to know each other better) | Demonstrate within the teams that positive relationships are encouraged and well accepted while negative talk/gossip is discouraged within the teams | Recognize/praise positive relationships that are developed by co-workers |  | Demonstrate how senior leaders and their relationships impact the organization positively |
| learn about mental illness | It is the norm to try to understand/ learn about MH | Co-workers learn /share knowledge about Mental health | The Source and Wellness initiatives provide opportunity to reinforce key messages about MH | Awareness of the resources available to learn more about MH | Help create a culture where it is expected that all employees learn about mental illness for the benefit of their co-workers |

| Mental Health – Co-worker  **External Determinants** | | | | | |
| --- | --- | --- | --- | --- | --- |
| Performance  Objectives | **Norms**  **and policies** | **Social support** | **Reinforcement** | **Resources** | **Organizational climate** |
| reach out to manager when needed | Make reaching out to managers the norm for co-worker who suspect their co-worker is suffering from MH | Co-worker encourage others to reach out to managers | The Source and wellness initiative emphasize take action with MH | High awareness of where to find information about co-worker role in MH |  |
| respect confidentiality | Understand policies around confidentiality of health information  It is the norm to respect confidentiality |  | Senior management demonstrates very high degree of importance to maintain confidentiality |  | Organizational climate is such that respect for confidentiality of heath information is a high priority |

| **Mental Health – Senior management Individual Determinants** | | | | |
| --- | --- | --- | --- | --- |
| **Performance**  **Objectives** | **Knowledge, capability or skill** | **Attitudes, beliefs and values** | **Expectations** | **Self efficacy** |
| -leadership to prioritize mental health  -invest time and budget | Increase sensitivity around mental health within Group Benefits and throughout other business units  Make mandatory mental health training (currently in place for Group Benefits) .  for all business units and as a on-demand resource. | Opportunity within other areas of the business to enhance attitudes, beliefs and values toward mental health, and for these attitudes to cascade down to employees | Expectation that there will be a movement within the company to invest more time/budget in mental health | Maintain/increase self confidence within Group Benefits and other business units to make MH a priority |
| -walk the talk  - lead by example  - mentorship for prevention  - be an advocate for a healthy workplace | Understand important role and learn skills to lead by example | Acquire the attitude that it is your responsibility to lead by example in MH | Those who have experienced mental health issues could share their personal story, in order to show their understanding, the impact on them, add a personal touch  (requires creation of opportunity to share mental health story, similar to the current opportunities to share other stories – e.g. fitness, physical activity | Build self confidence to lead by example in MH |
| **Performance**  **Objectives** | **Knowledge, capability or skill** | **Attitudes, beliefs and values** | **Expectations** | **Self efficacy** |
| -training to develop positive relationships with employees  -learn to identify behavioural issues | Continue to move toward mandatory training for all managers to enhance knowledge, capability, skill in the area of mental health | Develop attitude that positive relationship are important and it they have a role to foster/encourage positive relationship with employees | Provide opportunities to facilitate dialogue in this area. E.g. sharing of personal stories  Share stories after training that demonstrates how managers were able to help employees / felt better equipped to support the team due to the training | Share stories after training that demonstrates how managers were able to help employees / felt better equipped to support the team due to the training |
| **Performance**  **Objectives** | **Knowledge, capability or skill** | **Attitudes, beliefs and values** | **Expectations** | **Self efficacy** |
| -demonstrate flexibility and compassion  - foster open communication  - recognize each employees' 'normal'  - Provide positive recognition of employees | Mandatory training will help enhance knowledge and skills around mental health |  | Through training, EAP training and support services, and other means of education there will be the expectation that senior management will foster open communication and provide positive recognition | Develop self confidence to be able to foster compassion, open communication and recognition of employees |

**Mental Health – Senior management**

| Mental Health -**organization External Determinants** | | | | | |
| --- | --- | --- | --- | --- | --- |
| Performance  Objectives | **Norms**  **and policies** | **Social support** | **Reinforcement** | **Resources** | **Organizational climate** |
| -benchmarking - comparing organization to others re mental health | Continue with benchmarking of mental health indicators |  | Use benchmarking as a means to provide feedback on how we are doing relative to other companies and relative to past company measures  Use the data to help facilitate learning and change throughout the business (managers) |  | Leverage the data most effectively |
| - establish mission and philosophy around health of employees  - set the tone for the culture | Establish that employee health is a top priority  Recognition among senior managers that health is a key component of achieving the goal of being the best performing life insurance company in Canada | Develop a social support throughout the company – from top down around MH goals and objectives | Establish MH as a priority and establish metrics to measure success in this area (e.g. health index) | Maintain adequate resources to conduct analysis around performance in MH | Establish a culture where as part of a definition of high performance culture imbedded in this is the importance of a healthy organization |
| Performance  Objectives | **Norms**  **and policies** | **Social support** | **Reinforcement** | **Resources** | **Organizational climate** |
| - communicate expectations and procedures to managers and employees | Make MH and wellness part of managers objectives/performance mgmt. plan |  | Delivering of messages from senior mgmt across to employees through avenues such as videos  Anchor it to the high performance culture, connected to organization view on org health/our desire to ensure we have a healthy workforce message  Anchor it to other key overarching messages | Provide messages and resources to senior management to clarify expectations and procedures  Ensure that messages are clear for senior management to trickle down to employees | Delivering of messages from senior mgmt across to employees through avenues such as videos  Anchor it to the high performance culture, connected to organization view on org health/our desire to ensure we have a healthy workforce message  Anchor it to other key overarching messages |
| Performance  Objectives | Norms  and policies | Social support | Reinforcement | Resources | Organizational climate |
| - align policies and procedures to philosophy | Mental health is a consideration throughout decision making processes within the business  Put decisions through the mental health lens to consider the impact to employee health  Norm around communication between managers and employees |  | There is an understanding that all policies and procedures will be also measured and assessed on how they impact employee health |  | Establish a culture where employee health is paramount in all decisions around policies and procedures |

| Mental Health -**organization External Determinants** | | | | | |
| --- | --- | --- | --- | --- | --- |
| Performance  Objectives | **Norms**  **and policies** | **Social support** | **Reinforcement** | **Resources** | **Organizational climate** |
| know the standards from regulatory bodies (e.g. mental health commission of Canada –  - enforce policies and procedures | Understand new regulations and create a plan for implementation |  | through the commission there will be a push for employers to mandate education around mental health; employees need to accommodate; may drive what we do around mental health policies and procedures) | provide necessary resources to educate and reach out to Project Leader, Total Benefits and provide means to assess adherence | Establish a climate that are policies adheres and goes beyond regulations recommended by the commission |
| - provide resources for training managers/ supervisors/employees  -invest in social capital | Investment in social capital is the norm | There is support throughout organization for training and investment is social capital | Establishes procedures to provide feedback on success of training and ensure all managers/supervisors and senior management receives training | we provide necessary resources for training and establishing high social capital | There is a climate that highly values training in wellness and MH and investment in social capital |

| **Mental Health –family/partner/community** Individual Determinants | | | | |
| --- | --- | --- | --- | --- |
| Performance  Objectives | **Knowledge, capability**  **or skill** | **Attitudes, beliefs and values** | **Expectations** | **Self efficacy** |
| - supportive environment  - understanding of mental health and reduced stigmatization  - understanding of the impact of mental health on the individual  - discuss openly and honestly with the person having mental health problems | Establish understanding of MH and role family/partner/community plays in reducing negative impact  Make education/information on MH easily accessible and available  (currently sponsoring Dalhousie youth mental health awareness)  (Company may directionally be moving toward thought leadership in mental health – decisions to be made)  Learns skills on open communication, providing positive support and compassion | Demonstrate a caring and supportive attitude  Establish a belief that we all play a role in MH | There is an expectation that everyone needs to be supportive and have a understanding of MH and how they can help in a positive way | Develop the self confidence to acquire and act on the knowledge about MH and role of community/partner/friend |
| - recommendation to seek help, not be afraid to seek help  (identification of the signs) | Acquire the knowledge how to identify MH | Establish attitude and belief that we all have responsibility to identify danger signals and recommend they seek help | Know where they should seek help….what resources are available | Develop the self confidence to recommend to seek help |
| - foster awareness about available support and programs (community perspective)  have resources available for people who need help when they need it (community) | Acquire the knowledge of available resources in community |  | Provide the necessary advice is expected  All communities should have resources available and there is a high awareness of this information in community |  |
| bringing awareness and providing resources to all demographics | Provide the necessary knowledge to all demographics | Establish the belief that no one is immune to MH and that everyone should have access to the necessary information on how to help |  |  |

| **Mental Health - family/partner/community** **External Determinants** | | | | | |
| --- | --- | --- | --- | --- | --- |
| Performance  Objectives | **Norms**  **and policies** | **Social support** | **Reinforcement** | **Resources** | **Organizational climate** |
| - understanding of mental health and reduced stigmatization  - understanding of the impact of mental health on the individual  - discuss openly and honestly with the person having mental health problems | It is the norm that the community is educated on MH issues | There is support among all members of the community of they importance of MH | The community shares positive stories around MH | Community has the necessary resources to educate members | Establish a climate in the community that MH is important that we all should be aware of the stigma and discuss openly |
| - recommendation to seek help, not be afraid to seek help  (identification of the signs) | Establish as the norm in community that we should recognize the signs and advice to seek help | Demonstrate the attitude that we all have a responsibility to prevent consequences of MH | Share stories among friend/community | Ensure that resource available to learn more about the signs are widely available and accessible | Establish a climate that we need to take responsibility to identify those in need and recommend they seek help |
| - foster awareness about available support and programs (community perspective)  have resources available for people who need help when they need it (community) | Establish the norm that everyone should know about available support and resources |  | There is a means to establish awareness through sharing of stories and highlighted in community events | Community provides necessary resources to educate and increase awareness | Community establishes MH a high priority and provides necessary resources |
| bringing awareness and providing resources to all demographics | The norm is that all members of community can be impacted and that everyone has a role to help |  |  | Resources are available to educate all segments of the community-ethic groups all ages, most vulnerable |  |

| **Mental Health –health care providers** Individual Determinants | | | | |
| --- | --- | --- | --- | --- |
| Performance  Objectives | **Knowledge, capability**  **or skill** | **Attitudes, beliefs and values** | **Expectations** | **Self efficacy** |
| - ability to screen for individuals at high risk and provide appropriate interventions (e.g. referral, medication, counseling, etc.) | Demonstrate/acquire the necessary skills and knowledge | Acquire the belief that early identification and treatment is key to prevention | Demonstrate skills and knowledge and to act on this knowledge | Demonstrate high confidence to identify and provide necessary treatment/advice |
| stay up-to-date and current with regards to mental health trends in Canada | Demonstrate the ability to keep current | Attitude that it is their responsibility to keep up to date | Responsibility to stay current |  |
| - having more dialogue with the employee, providing teaching and information | Acquire the knowledge and skills to inform employees to discuss MH | Acquire the belief that dialogue is important to the health care provider/employee relationship | The expectation is that they will discuss, educate and inform employees |  |
| holistic approach (combination of medications, therapy, etc.) commitment to keep working at a treatment plan | To be aware of the various approaches to treatment and be open to employee preferences and a holistic approach. | Acquire the attitude that a holistic approach can be an option for treatment – be open to different possibilities and combinations | It is expected that all treatment options will be discussed and offered to employees | Develop self confidence to use an holistic approach and involve employees in decision process around treatment options |
| follow-up - is the treatment plan working? what adjustments are needed? ongoing case management at the doctor-patient level | Acquire the knowledge around the need to follow-up and monitor progress | Establish the attitude that follow-up and monitoring is important | There is the expectation that there will be follow-up and monitoring and modifying treatment as necessary |  |

| **Mental Health - health care providers** (HCP) **External Determinants** | | | | | |
| --- | --- | --- | --- | --- | --- |
| Performance  Objectives | **Norms**  **and policies** | **Social support** | **Reinforcement** | **Resources** | **Organizational climate** |
| - ability to screen for individuals at high risk and provide appropriate interventions (e.g. referral, medication, counseling, etc.) | It is the norm to provide appropriate care including screening and treatment | Other HCP provide positive support to provide appropriate care | Describe how others are providing excellent care and using appropriate guidelines  Highlight in community what is high quality care | Ensure sufficient resources are available to provide the appropriate care | There is a climate in the community and among other HCP that appropriate screening and treatment is provided |
| - stay up-to-date and current with regards to mental health trends in Canada | It is the norm to stay up to date on MH care and provide dialogue and information and teaching on MH | Demonstrate positive social pressure in the community to stay up to date | Provide feedback on how others keep up to date | Have available educational material that HCP can use to educate | Describe a climate where it is expected the HCP stay up to date on MH issues…screening and management |
| - having more dialogue with the employee, providing teaching and information |  |  |  | Provide resources for HCP to easily pass on to employees |  |
| follow-up - is the treatment plan working? what adjustments are needed? ongoing case management at the doctor-patient level | It is the norm to provide the appropriate follow-up and to regularly assess progress/ management effectiveness and outcomes in these individuals | there is positive social support in providing appropriate follow-up | Demonstrate examples where follow-up was essential for improved outcomes | Demonstrate the value of follow-up to the complete care in MH | The climate exists where follow-up and re-assessment is expected for quality care |

**Matrix B**

| **MSK Disorders – Employee Individual Determinants** | | | | |
| --- | --- | --- | --- | --- |
| **Performance**  **Objectives** | **Knowledge, capability or skill** | **Attitudes, beliefs and values** | **Expectations** | **Self efficacy** |
| -if they have physical limitations, need to communicate with their manager  - knowing when to seek help if they have physical pain  reduce physical work hazards  -adapt/accommodate worksite  - take regular breaks | Understand the importance of repetitive strain and worksite ergonomics. Describe or explain what help is available to prevent repetitive strain.  Explain that there are resources available that will tell you how to seek help (who, where to go)  Understand the importance of adapting the worksite to accommodate any limitations and/or risk  Acquire knowledge about regular rest breaks | Explain that by seeking ergonomic help this may prevent injuries. | Demonstrate that people that seek help can be helped | Provide the resources to foster the self-confidence for people to know when and where to seek help when they need it |

**Musculoskeletal (MSK) Disorders – Employee**

| use available resources | Describe where they can find available resources when they are having a MSK health issues (e.g. ergonomics assessment request through Source) |  |  | Improve confidence in seeking out available resources |
| --- | --- | --- | --- | --- |
| - take the appropriate medication | Describe the benefits of medication compliance in MSK pain.  Provide information on benefits site /wellness centre /health library on preventing MSK injuries/pain | Explain that taking as recommended medication may improve function and reduce pain | Describe that it is okay to seek support (also applies to attitudes) | Build confidence to seek help |

| **MSK Disorders - Employee Individual Determinants** | | | | |
| --- | --- | --- | --- | --- |
| **Performance**  **Objectives** | **Knowledge, capability or skill** | **Attitudes, beliefs and values** | **Expectations** | **Self efficacy** |
| regular exercise (30 min/day)  seek advice on appropriate exercise/self management | Explain how exercise is beneficial to MSK health – links on Source to articles for example  Demonstrate how you can find 30 minutes per day to exercise  Explain how exercise can improve your work performance, increase efficiency, reduce MSK pain  Seek advice on appropriate exercises for specific MSK pain. | Explain that it is possible to find 30 min per day to exercise – provide tips and examples | Build positive expectations around the ability to exercise and the benefits that can be realized. | Build confidence to find 30 minutes during the day to exercise and that it will not impact work performance  Improve confidence in seeking out available resources |
| maintain adequate weight  - proper diet  - lifestyle, walking | Explain how one can achieve adequate nutrition (low fat, high fibre, low sugar, calories) and where to find the resources to assist them in getting better nutrition  Adequate weight will help reduce pain from arthritis of knees and hips  Wellness Centre available 24/7 to get more information on lifestyle; medication; conditions; etc.  Explain the relationship between healthy living and MSK pain | Explain that it is possible to eat properly (provide tips and links)  Explain how proper diet/nutrition can positively impact health | Build positive expectations around the ability to have nutritious meals | Build confidence to seek/make nutritious meals  Provide tips on how to eat healthy  Improve confidence in seeking out available resources |
| sit more ergonomically-appropriate work posture (reduce physical strains of sitting) | Provide knowledge on available resources for information on ergonomics  Provide knowledge on how to get ergonomic assessment | Acquire attitude/belief that proper ergonomics can impact MSK pain both prevention and management | Expectation that employees have knowledge on proper ergonomic involving worksite (work and home) | Improve confidence in seeking out available resources |

| **MSK Disorders – Employee Individual Determinants** | | | | |
| --- | --- | --- | --- | --- |
| **Performance**  **Objectives** | **Knowledge, capability or skill** | **Attitudes, beliefs and values** | **Expectations** | **Self efficacy** |
| develop positive relationships with co-workers/manager/ supervisors | Understanding that positive relationships reduce the negative impact of MSK disorders | Explain that positive workplace relationships can improve workplace health |  |  |
| Engage in stress/time management  work/life balance | Learn how to better manage time and stress  Describe the available resources to help work/life balance  Describe linkages between stress management and MSK issues  Wellness Centre information. Some of the wellness programs target stress management  Understand the role of work/life balance in MSK health | Show that it is possible to have work/life balance  Demonstrate that it is OK to seek help for stress management | Persuade that it is possible to reduce stress  Demonstrate that it is okay to create work/life balance in a way that works for you | Build confidence to seek help when work/life stress is totally out of balance |
| if at risk for arthritis - need to have it checked out by health care person  aware of links between muscul-skeletal health and depression | Explain that if MSK pain is impacting work/ home life should seek advice from health professional.  Explain there is a strong association between MSK pain and depression | Establish attitudes and beliefs to be proactive and seek preventive measures | Establish expectations that regular check up is necessary if high risk for pain and disability due MSK disorder |  |

| **MSK Disorders – Employee External Determinants** | | | | | | |
| --- | --- | --- | --- | --- | --- | --- |
| Performance  Objectives | | **Norms**  **and policies** | **Social support** | **Reinforcement** | **Resources** | **Organizational climate** |
| -if they have physical limitations, need to communicate with their manager  - knowing when to seek help if they have physical pain  reduce physical work hazards  -adapt/accommodate worksite  - take regular breaks | | Promote/reinforce the Norm that we will support employee that seeks help.  Make it the norm to encourage regular breaks | Emphasize that if employees seek help they will be supported by their team/manager | Provide stories in The Source emphasis on support of managers to those who seek help | Enhance visibility of resources available to employees on MSK health | Promote an organizational culture where it is okay to seek help and where SL will provide necessary support |
| use available resources | Demonstrate how team leaders can provide information/promote resources to team members (e.g. in team meetings)  Other examples: promoting Wellness Centre, smoking cessation aids, other | |  | Demonstrate using data or graphs the utilization of the resources by employees  Host a fair where vendors can provide education and information on services available | Demonstrate that resources are readily accessible; enhance communication and visibility around the resources  Enhance visibility through communicating in common areas (e.g. cafeterias) | Encourage the use of the available resources when needed |

| **MSK Disorders - Employee External Determinants** | | | | | |
| --- | --- | --- | --- | --- | --- |
| **Performance**  **Objectives** | **Norms**  **and policies** | **Social support** | **Reinforcement** | **Resources** | **Organizational climate** |
| medication compliance/appropriate  use | describe that the norm to have resources available on appropriate use of medication | provide through benefits program to info on medication use  (eg Narcotics) | educate employees on the importance of compliance; reinforce that the organization is here to support employees  Provide feedback using HRA | Use flex benefits  and microsite to educate  Encourage use of EAP communications  Include info on Source  Provide information on the importance of medication adherence |  |
| do regular exercise (at least 20 minutes of walking 5 out 7 days per week) | Demonstrate via mentorship by management; role models for exercise and get away from their desk on lunch. Make 20 min of exercise the Norm.  Explain the norm to get expert advice to ensure appropriate exercises for MSK problem | Provide a forum for individuals looking for social support to promote physical activity (i.e. discussions for people to look for group fitness activities) | Use verbal reinforcement and reward positive behaviour  sharing of best practices (e.g. collaboration tool or forum to share what employees/managers are doing) | Use videos  (e.g 23 1/2 hours)  Wellness programs such as health challenges  Reinforce programs such as Weight Watchers at Work, fitness reimbursements, Wellness Account, on-site gyms | senior leaders demonstrate participating in national wellness day and other wellness programs offered |

| **MSK Disorders– Employee External Determinants** | | | | | |
| --- | --- | --- | --- | --- | --- |
| Performance  Objectives | **Norms**  **and policies** | **Social support** | **Reinforcement** | **Resources** | **Organizational climate** |
| seek advice on appropriate exercise/self management  maintain adequate weight  - proper diet  - lifestyle, walking | show role modeling; walking the walk; healthy snacks and meals for meetings; where cafeterias exist, ensuring healthy options  discuss about the community norms shifting toward healthy food/drink choices  Provide more option in drinks like juices rather than coffee or pop | Demonstrate peer to peer support around positive eating habits;  collaboration spaces or forums to share success stories; profile leaders favorite recipes | Demonstrate positive reinforcement by managers on healthy choices by employees | healthy cafeteria food; information on the wellness -part of the Source website e.g. healthy recipes | Senior management demonstrates the importance of proper nutrition and healthy lifestyle and how this is important for a high performance culture |

| **MSK Disorders – Employee External Determinants** | | | | | |
| --- | --- | --- | --- | --- | --- |
| Performance  Objectives | **Norms**  **and policies** | **Social support** | **Reinforcement** | **Resources** | **Organizational climate** |
| Engage in stress/time management  work/life balance | Describe flex time schedule to enable employees to accommodate home situation and have time flexibility  Encourage telecommuting and use of flex hours  Encourage discussions between management and employees to regularly check in on stress/time management and work/life balance | Describe how peers encourage and support others who attempt to achieve work-life balance | Directors/ leaders demonstrate work life balance  On The Source have stories/testimonials that may have an effect to model other to achieve the same | Health and wellness programs that focus on stress mgmt, work life balance, time mgmt  Provide education and opportunities to learn relaxation and stress management eg, lunch and learn  Promote the EAP as resource to get help  Continue to promote wellness programs that target stress mgmt.  Share specialized training information to other departments in the organization (e.g. Market Development conducted time mgmt. training) | Describe a climate by which employee attempt to achieve work-life balance  Describes a climate where work-life balance is valued |

| **MSK Disorders– Manager/Supervisor Individual Determinants** | | | | |
| --- | --- | --- | --- | --- |
| **Performance**  **Objectives** | **Knowledge, capability**  **or skill** | **Attitudes, beliefs and values** | **Expectations** | **Self efficacy** |
| be educated about musculo-skeletal disorders  Focus on function – how is the condition impacting ability to perform the job  observe people sitting at their desk - identifying and suggesting an ergonomic assessment  monitor physical/stress demands  suggests/accommodate to worker abilities/tolerances | Acquire the knowledge and skills to identify employees in MSK distress and know available resources for employee to seek help. Include as part of manager training, identifying MSK problems and how to deal with them  Accountability to point out when there are gaps in ability to function or change in performance – then strongly encourage to seek professional help | Eliminate negative attitudes toward MSK problems | Expectations that managers will acquire knowledge to deal appropriately with employees with MSK health issues  Knowing when to suggest an ergonomic assessment | Build confidence for managers to identify and take appropriate steps when presented with MSK health issues |
| role model (eating properly, exercising, going out for a walk at lunch time, work-life balance, adequate weight) | Understand that actions of superiors impact behaviours of employees. |  | The expectations that managers will act a s role models on healthy living  Expected that managers  We act as role models using appropriate ergonomics at workplace | Build confidence among managers to know when to refer for ergo assessment and how to maintain proper work posture and ergonomically sound work space |
| -follow processes  -know when to seek help  - minimize ambiguity in employee roles  awareness of resources available to employees (e.g. wellness programs, fitness programs) | Be aware of available resources and what procedures to follow | Encourage attitude that manager and supervisors have an important role in MSK health helping to adapt the workplace to the health needs. | Provide training on processes, role and resources available | Demonstrate the confidence to follow processes, when to ask for help and knowledge of available resources |

| **MSK Disorders – Manager/Supervisor Individual Determinants** | | | | |
| --- | --- | --- | --- | --- |
| **Performance**  **Objectives** | **Knowledge, capability or skill** | **Attitudes, beliefs and values** | **Expectations** | **Self efficacy** |
| -mentorship for prevention  -encourage employees to participate in wellness | Understand that positive (and negative) actions of superiors impact behaviours of employees. | Express belief that participation is important in prevention | Encourage participation in the wellness programs |  |
| - help enable employee to get better | Acquire skills and knowledge to enable employees to seek help they need. |  | Demonstrate willingness  To help | Demonstrate confidence to provide resources to employees to get better (knowing what to provide, when to provide it, etc.) |
| develop positive relationships with employees/subordinates - can lead to open communication, reduced stress, show concern | Communicate ways to recognize employees  Obtain training on how to praise and recognise  Profile on intranet recognition  Provide positive reinforcement. | Understand the importance of recognition | Provide positive recognition – use tools available on the source such as VIP recognition | Demonstrates confidence to provide praise and recognition |

| **MSK Disorders – Manager/Supervisor External Determinants** | | | | | |
| --- | --- | --- | --- | --- | --- |
| Performance  Objectives | **Norms**  **and policies** | **Social support** | **Reinforcement** | **Resources** | **Organizational climate** |
| be educated about musculoskeletal disorders  Focus on function – how is the condition impacting ability to perform the job  observe people sitting at their desk - identifying and suggesting an ergonomic assessment  monitor physical/stress demands  suggests/accommodate to worker abilities/tolerances | Inform that it is the norm (policy) that all managers/supervisors obtain training on best practices  Comparison with other organizations (feedback)  Understanding policy around procedures on how to act when function is impacted due to MSK problem | Other manager provide support on how to manage MSK problems  Encourage support among managers | Senior management recognizes those who have helped others with MSK disorders. The Source highlights role  Opportunity to share experiences with other managers on how to act appropriately.  Senior management/ company publications/ wellness website/training manuals, The Source all  Provide information on available resources for MSK disorders for managers | Management provides sufficient resources for education and training  Have refresher training sessions  Enhance visibility of resources available to managers/supervisors  Have a go to person for advise/guidance | Demonstrated climate of understanding and open dialogue among managers/supervisors on how to manage effectively when MSK disorders impact work ability. |

| **MSK Disorders – Manager/Supervisor External Determinants** | | | | | |
| --- | --- | --- | --- | --- | --- |
| Performance  Objectives | **Norms**  **and policies** | **Social support** | **Reinforcement** | **Resources** | **Organizational climate** |
| -mentorship for prevention  --encourage employees to participate in wellness  role model (eating properly, exercising, going out for a walk at lunch time, work-life balance, adequate weight) | It is the norm that managers act as role models in MSK health and participate in wellness initiatives and encourage employees to participate | Manager encourage other managers to be role models and participate in wellness initiatives – discuss wellness in leader meetings | Senior management recognized managers who participate in wellness programs and act as role models in MSK prevention |  | Construct a climate where everyone wants to participate in MSK wellness initiatives |
| - help enable employee to get resources  awareness of resources available to employees (e.g. wellness programs, fitness programs)  better | Define the norm/policy that managers be trained how to enable employees to help themselves | Managers assist each other on how to enable employees |  | Managers receive the necessary training on how to enable employees | Construct a climate where managers want to help enable employees |
| Provide positive recognition of employees  develop positive relationships with employees/subordinates - can lead to open communication, reduced stress, show concern | Describe it is the norm to provide positive recognition to employees |  | Reinforcement by senior leadership of managers/supervisors who provide positive recognition of employees | Provide necessary training on how to provide positive recognition of employees | Senior management demonstrate positive recognition of managers |

**MSK Disorders– Co-worker**

| MSK Disorders– Co-worker Individual Determinants | | | | |
| --- | --- | --- | --- | --- |
| Performance  Objectives | **Knowledge, capability**  **or skill** | **Attitudes, beliefs and values** | **Expectations** | **Self efficacy** |
| be supportive - Work together common goals and objectives-show compassion and concern | Inform co-workers on importance of being supportive and showing compassion | Develop a supportive and caring attitude for workers with MSK disorders | All co-workers work together and show concern and compassion | Demonstrates confidence in showing compassion and concern |
| help create a positive environment  positive healthy role models(eating, exercise, weight, attitudes, relationships, use of proper ergonomic, work-life balance) | Develop skills to develop and maintain a positive environment  Acquire knowledge to act as role model for other co-workers in MSK health | Show how to act into a new way of thinking. Demonstrate that a positive environment is a fun place to work  Demonstrate positive work/life balance and positive attitude | All employees are expected to be role models for each other in healthy behaviours | Demonstrate that employees have the ability to provide suggestions in creating a positive environment |
| awareness of signs, identify amongst co-workers (fostering knowledge) who are suffering from MSK disorders- acquire basic knowledge | Provide accessible information about MSK disorders and prevention for all employees | Demonstrate the belief that knowing about MSK health is important | It is expected that all co-worker learn where they can seek out info on MSK health and prevention | Demonstrate confidence in knowing where to learn about MSK health |

| MSK Disorders – Co-worker Individual Determinants | | | | |
| --- | --- | --- | --- | --- |
| Performance  Objectives | **Knowledge, capability or skill** | **Attitudes, beliefs and values** | **Expectations** | **Self efficacy** |
| reach out to manager when needed | Understand the importance to reach out to managers when you suspect MSK problem among co-workers | Describe the attitude that we all have a role in MSK health -identification and prevention | It is the norm to reach out to managers when suspect MSK problem in co-worker | Demonstrate the confidence to reach out to manager when suspect MSK disorder among co-worker |
| respect confidentiality | Obtain the knowledge around importance of maintaining confidentiality | Demonstrate high level of respect for confidentiality around MSK health issues of co-workers | It is the norm not to discuss the MSK problems of co-workers with other persons |  |

| **MSK Disorders– Co-worker**  **External Determinants** | | | | | |
| --- | --- | --- | --- | --- | --- |
| Performance  Objectives | **Norms**  **and policies** | **Social support** | **Reinforcement** | **Resources** | **Organizational climate** |
| support, positive relationships show compassion and concern | Make it the norm that co-worker are supportive and concern for other co-workers with MSK Health issues | Describe an environment where co-workers support each other | Co-workers are praised for supporting and showing compassion | The Source provides examples and re-enforces a supportive environment. Wellness programs encourage a supportive workplace | Organizational climate is such that a supportive and compassionate workplace is the norm |
| positive healthy role models(eating, exercise, weight, attitudes, relationships, use of proper ergonomic, work-life balance) | Demonstrate how teams who have positive relationships impact the work environment (e.g. socializing with teams; creating opportunities to know each other better)  It is the norm to strive for  Healthy living and act as role models | Demonstrate within the teams that positive relationships are encouraged and well accepted while negative talk/gossip is discouraged within the teams  Co-workers support each other to achieve healthy behaviours | Recognize/praise positive relationships that are developed by co-workers and healthy behaviours |  | Demonstrate how senior leaders and their relationships impact the organization positively |
| awareness of early signs, identify amongst co-workers (fostering knowledge) | It is the norm to try to understand/ learn about MSK health | Co-workers learn /share knowledge about MSK health and prevention | The Source and Wellness initiatives provide opportunity to reinforce key messages about MSK health | Awareness of the resources available to learn more about MSK health | Help create a culture where it is expected that all employees learn about MSK health |

| **MSK Disorders- Co-worker**  **External Determinants** | | | | | |
| --- | --- | --- | --- | --- | --- |
| Performance  Objectives | **Norms**  **and policies** | **Social support** | **Reinforcement** | **Resources** | **Organizational climate** |
| reach out to manager when needed | Make reaching out to managers the norm for co-worker who suspect their co-worker is suffering from MSK problem that is impacting work | Co-worker encourage others to reach out to managers | The Source and wellness initiative emphasize take action with MSK Disorder | High awareness of where to find information about co-worker role in MSK Disorders and prevention |  |
| respect confidentiality | Understand policies around confidentiality of health information  It is the norm to respect confidentiality |  | Senior management demonstrates very high degree of importance to maintain confidentiality |  | Organizational climate is such that respect for confidentiality of heath information is a high priority |

| **MSK Disorders– Senior management Individual Determinants** | | | | |
| --- | --- | --- | --- | --- |
| **Performance**  **Objectives** | **Knowledge, capability or skill** | **Attitudes, beliefs and values** | **Expectations** | **Self efficacy** |
| -invest time and budget  allow for budgeting for programs/training related to MSK Disorders (providing time and resources to educate) | Increase knowledge on impact of MSK Disorders on presenteeism.  Provide opportunities to education the importance of these health issues at workplace. | Opportunity within other areas of the business to enhance attitudes, beliefs and values toward MSK health for these attitudes to cascade down to employees | Expectation that there will be a movement within the company to provide necessary investment in management/prevention of MSK at workplace |  |
| -walk the talk  - lead by example  - mentorship for prevention  - be an advocate for a healthy workplace  - positive role models, walk the talk (diet, exercise, lifestyle, work-life balance) lead by example  - make sure they are sitting properly with the correct equipment  Positive relations with managers/employees | Understand important role and learn skills to lead by example  Understand the important impact of developing and maintaining positive relationships on negative consequences of MSK disorders | Acquire the attitude that it is your responsibility to lead by example in MSK health | Those who have experienced MSK health issues could share their personal story, in order to show their understanding, the impact on them, add a personal touch  (requires creation of opportunity to share health story, similar to the current opportunities to share other stories – e.g. fitness, physical activity | Build self confidence to lead by example in MSK health |
| **Performance**  **Objectives** | **Knowledge, capability or skill** | **Attitudes, beliefs and values** | **Expectations** | **Self efficacy** |
| -demonstrating accommodation, flexibility | training will help enhance knowledge and skills around MSK disorders |  | Through training, EAP training and support services, and other means of education there will be the expectation that senior management will foster open communication and provide positive recognition | Develop self confidence to be able to foster compassion, open communication and recognition of employees |

| **MSK Disorders- organization External Determinants** | | | | | |
| --- | --- | --- | --- | --- | --- |
| Performance  Objectives | **Norms**  **and policies** | **Social support** | **Reinforcement** | **Resources** | **Organizational climate** |
| -benchmarking - comparing organization to others re MSK health  Integrated data analysis | Continue with benchmarking of MSK health indicators (eg claims) |  | Use benchmarking as a means to provide feedback on how we are doing relative to other companies and relative to past company measures  Use the data to help facilitate learning and change throughout the business (managers) |  | Leverage the data most effectively |
| - establish mission and philosophy around health and wellness of employees  - set the tone for the culture  mission statement, main message around health in general  culture - how important is the well-being of our employees  instill/facilitate a health/safety culture  -invest in social capital | Establish that employee health is a top priority  Recognition among senior managers that health is a key component of achieving the goal of being the best performing life insurance company in Canada | Develop a social support throughout the company – from top down around employee health goals and objectives | Establish employee health as a priority and establish metrics to measure success in this area (e.g. health index) | Maintain adequate resources to conduct analysis around performance in employee health  -invest in social capital | Establish a culture where as part of a definition of high performance culture imbedded in this is the importance of a healthy organization |
| Performance  Objectives | **Norms**  **and policies** | **Social support** | **Reinforcement** | **Resources** | **Organizational climate** |
| - communicate expectations and procedures to managers and employees  communication around expectations re: health/ wellness-make health/wellness the norm | Make wellness and prevention part of managers objectives/performance mgmt. plan  - communicate around expectations re: health/safety/wellness-make health/wellness the norm |  | Delivering of messages from senior mgmt across to employees through avenues such as videos  Anchor it to the high performance culture, connected to organization view on org health/our desire to ensure we have a healthy workforce message  Anchor it to other key overarching messages | Provide messages and resources to senior management to clarify expectations and procedures  Ensure that messages are clear for senior management to trickle down to employees | Delivering of messages from senior mgmt across to employees through avenues such as videos  Anchor it to the high performance culture, connected to organization view on org health/our desire to ensure we have a healthy workforce message  Anchor it to other key overarching messages |
| - align policies and procedures to philosophy  policies and procedures related to wellness and prevention | Employee health is a consideration throughout decision making processes within the business  Put decisions through the employee health lens to consider the impact to employee health  Norm around communication between managers and employees |  | There is an understanding that all policies and procedures will be also measured and assessed on how they impact employee health |  | Establish a culture where employee health is paramount in all decisions around policies and procedures |

| **MSK Disorders**-**organization External Determinants** | | | | | |
| --- | --- | --- | --- | --- | --- |
| Performance  Objectives | **Norms**  **and policies** | **Social support** | **Reinforcement** | **Resources** | **Organizational climate** |
| - provide resources for training managers/ supervisors/employees  -invest in human capital | Investment in social capital is the norm | There is support throughout organization for training and investment is social capital | Establishes procedures to provide feedback on success of training and ensure all managers/supervisors and senior management receives training | We provide necessary resources for training and establishing high social capital | There is a climate that highly values training in health and wellness and investment in social capital |

| **MSK Disorders- family/partner/community** Individual Determinants | | | | |
| --- | --- | --- | --- | --- |
| **Performance**  **Objectives** | **Knowledge, capability**  **or skill** | **Attitudes, beliefs and values** | **Expectations** | **Self efficacy** |
| - education around how to lift properly  - education on proper ergonomic at home  - understand the relationship between chronic MSK and depression/anxiety | Provide a mechanism where employee shares workplace educational resources with family/partner to establish understanding of MSK health and role family/partner/community plays in reducing negative impact  Make education/information on MSK health easily accessible and transferable to family/partner | Demonstrate a caring and supportive attitude  Understanding of the serious negative consequences of poor MSK and the role of family in prevention | There is an expectation that everyone needs to be aware of the negative consequences and how they can help in a positive way | Develop the self confidence to acquire and act on the knowledge about MSK health and role of community/partner/friend |
| - foster awareness about available support and programs (community perspective)  have resources available for people who need help when they need it (community) | Acquire the knowledge of available resources in community |  | Provide the necessary advice is expected  All communities should have resources available and there is a high awareness of this information in community |  |
| encourage healthy eating, diet, nutrition at home  - encourage exercise, more walking  encourage physical activity, healthy weight  - model for healthy living  - awareness of the role and potential negative consequences of narcotic use for pain  - understanding role of self-management in chronic MSK disorders  - provide encouragement and support for ongoing treatment | Provide the necessary knowledge to all demographics the importance of healthy living to prevent disability due to MSK disorders  Provide the necessary support to encourage appropriate use of medication  Make available to community information on proper ergonomics and role of prevention  Encourage the self management of chronic MSK pain | Establish attitude that we all play a role in encouraging and reinforcing healthy living to prevent and adequately manage MSK disorders  Establish the attitude and belief that self-management is key factor in successful management of chronic MSK pain. | Expectation that we all should be role models for healthy living among friends and family  Expectation that family members encourage and support self management | Develop the self confidence to acquire the knowledge and to act to prevent disability due to MSK disorders |

| **MSK Disorders - family/partner/community** **External Determinants** | | | | | |
| --- | --- | --- | --- | --- | --- |
| Performance  Objectives | **Norms**  **and policies** | **Social support** | **Reinforcement** | **Resources** | **Organizational climate** |
| - education around how to lift properly  - education on proper ergonomic at home  - understand the relationship between chronic MSK and depression/anxiety | It is the norm that the community is educated on the prevention of disability due to MSK disorders and when to recommend to seek help and its association with depression/anxiety | There is support among all members of the community of the importance of MSK health and its prevention | The community shares positive stories around MSK health | Community has the necessary resources to educate members | Establish a climate in the community that MSK health is important |
| - foster awareness about available support and programs (community perspective)  have resources available for people who need help when they need it (community) | Establish the norm that everyone should know about available support and resources |  | There is a means to establish awareness through sharing of stories and highlighted in community events | Community provides necessary resources to educate and increase awareness | Community establishes MSK health as a priority and provides necessary resources |
| encourage healthy eating, diet, nutrition at home  - encourage exercise, more walking  encourage physical activity, healthy weight  - model for healthy living  - awareness of the role and potential negative consequences of narcotic use for pain  - understanding role of self-management in chronic MSK disorders  - provide encouragement and support for ongoing treatment | The norm is that all members of community can be impacted and that everyone has a role to help and act as role models  It is the Norm to act as a role model for healthy living  It is the norm to understand the negative consequences due to misuse of narcotics for chronic MSK pain. | Describe a community where there is support and encouragement for healthy living | Highlight where Community shares positive stories on how the community helps others with MSK pain and disability | Resources are available to educate all segments of the community-ethic groups all ages, most vulnerable  Make resources available  On narcotic use for MSK pain  Make resources available for education on self management |  |

| **MSK Disorders–health care providers** Individual Determinants | | | | |
| --- | --- | --- | --- | --- |
| Performance  Objectives | **Knowledge, capability**  **or skill** | **Attitudes, beliefs and values** | **Expectations** | **Self efficacy** |
| - ability to screen for individuals at high risk and provide appropriate interventions (e.g. referral, medication, etc.) and follow up | Demonstrate/acquire the necessary skills and knowledge  Demonstrate knowledge in evidence-based management of MSK pain | Acquire the belief that early identification and treatment is key to prevention | Demonstrate skills and knowledge and to act on this knowledge | Demonstrate high confidence to identify and provide necessary treatment/advice |
| stay up-to-date and current with regards to management of MSK pain  - aware of ergonomic issues, educate patients on proper ergonomics/ prevention  - recommend /refer for appropriate treatment/ management | Demonstrate the ability to keep current and knowledge on ergonomics  Know when and who to refer | Attitude that it is their responsibility to keep up to date | Responsibility to stay current |  |
| providing teaching and information  in terms of exercise and diet, - encourage to stay active  provide appropriate resources | Acquire the knowledge and skills to inform employees on self-management strategies | Acquire the belief that dialogue is important to the health care provider/employee relationship | The expectation is that they will discuss, educate and inform employees |  |
| follow-up - is the treatment plan working? what adjustments are needed? ongoing case management at the doctor-patient level  regular follow-ups to see if risk factors are under control  - communicate with workplace for work modification  - positive role model | Acquire the knowledge around the need to follow-up and monitor progress  And how to communicate with workplace | Establish the attitude that follow-up and monitoring is important | There is the expectation that there will be follow-up and monitoring and modifying treatment as necessary |  |

| **MSK Disorders- health care providers** (HCP) **External Determinants** | | | | | |
| --- | --- | --- | --- | --- | --- |
| Performance  Objectives | **Norms**  **and policies** | **Social support** | **Reinforcement** | **Resources** | **Organizational climate** |
| - ability to screen for individuals at high risk and provide appropriate interventions (e.g. referral, medication, etc.) and follow up | It is the norm to provide appropriate care including screening and treatment | Other HCP provide positive support to provide appropriate care | Describe how others are providing excellent care and using appropriate guidelines  Highlight in community what is high quality care | Ensure sufficient resources are available to provide the appropriate care | There is a climate in the community and among other HCP that appropriate screening and treatment is provided |
| stay up-to-date and current with regards to management of MSK pain  - aware of ergonomic issues, educate patients on proper ergonomics/ prevention  - recommend /refer for appropriate treatment/ management | It is the norm to stay up to date on MSK care and provide information and teaching on MSK health and prevention | Demonstrate positive social pressure in the community to stay up to date | Provide feedback on how others keep up to date | Have available educational material that HCP can use to educate | Describe a climate where it is expected the HCP stay up to date on MSK health …screening and management |
| providing teaching and information  in terms of exercise and diet, - encourage to stay active  provide appropriate resources | Describe the norm to provide appropriate education and treatment on management and prevention |  |  | Provide resources for HCP to easily pass on to employees |  |
| follow-up - is the treatment plan working? what adjustments are needed? ongoing case management at the doctor-patient level  regular follow-ups to see if risk factors are under control  - communicate with workplace for work modification  - positive role model | It is the norm to provide the appropriate follow-up and to regularly assess progress/ management effectiveness and outcomes in these individuals  Describe the norm where the HCP, employee and workplace communicate effectively and work together to maximize health of employee | there is positive social support in providing appropriate follow-up | Demonstrate examples where follow-up was essential for improved outcomes | Demonstrate the value of follow-up to the complete care in MSK pain | The climate exists where follow-up and re-assessment is expected for quality care |

**Matrix C**

**Cardiovascular– Employee**

| \| **Cardiovascular/diabetes– Employee Individual Determinants** \| \| \| \| \| \| --- \| --- \| --- \| --- \| --- \| \| **Performance**  **Objectives** \| **Knowledge, capability or skill** \| **Attitudes, beliefs and values** \| **Expectations** \| **Self efficacy** \| \| \| be aware of the signs and symptoms and when they do occur do something about it \| Describe or explain what help is available  Explain that there are resources available that will tell you how to seek help (who, where to go) \| Explain that by seeking help it will be dealt with in a confidential way  Explain or describe that CV-diabetes can be effectively treated \| Demonstrate that people that seek help can be helped \| Provide the resources to foster the self-confidence for people to know when and where to seek help when they need it \| \|   use available  reso | ues | Explain that by using available resources it will be dealt with in a confidential way | Build the expectation that resources should be available if employees seek them (e.g. at work, in the community) | Improve confidence in seeking out available resources |
| --- | --- | --- | --- | --- | --- | --- | --- | --- | --- | --- | --- | --- | --- | --- | --- | --- | --- | --- | --- | --- | --- |
| medication compliance/appropriate use of medication  be aware of the drugs that you are taking (drug interactions can impact cardiovascular health) | Describe the benefits of seeking support, medication compliance (how to, consequences of not following)  Minimum amount of information available on benefits site; information available on wellness centre through health library | Explain that taking as recommended medication will impact success | Describe that it is okay to seek support (also applies to attitudes) | Build confidence to seek support  Build confidence to manage your own compliance |

| **CV- Diabetes– Employee Individual Determinants** | | | | |
| --- | --- | --- | --- | --- |
| **Performance**  **Objectives** | **Knowledge, capability or skill** | **Attitudes, beliefs and values** | **Expectations** | **Self efficacy** |
| do regular exercise (at least 20 minutes of walking 5 out 7 days per week) | Explain how exercise is beneficial to CV health – links on Source to articles for example  Demonstrate how you can find 30 minutes per day to exercise  Explain how exercise can improve your work performance, increase efficiency  Wellness program available at certain times throughout the year – e.g. cardiovascular screening clinics; health challenges; education sessions/  webinars; online learning programs | Explain that it is possible to find 120 min per week to exercise – provide tips and examples  Explain how regular exercise can be beneficial to impact CV and/or diabetes | Build positive expectations around the ability to exercise and the benefits that can be realized. | Build confidence to find 30 minutes during the day to exercise and that it will not impact work performance |
| Get adequate sleep | Explain how one can achieve adequate sleep (7 hours) and where to find the resources to assist them in getting better sleep  Explain the difference between quantity and quality of sleep and how needs might differ between people | Explain that it is possible to obtain adequate sleep  Explain how adequate sleep can be beneficial to impact CV and/or diabetes | Build positive expectations around the ability to get good sleep | Build confidence in ability to utilize strategies to get better sleep |
| eat properly - reduce fat intake, increase fiber, increase greens reduce refined sugars/alcohol,  monitor diet, reduced processed foods and eating out  weight management | Explain how one can achieve adequate nutrition (low fat, high fibre, low sugar, calories) and where to find the resources to assist them in getting better nutrition  Same programs as per above  Wellness Centre available 24/7 to get more information on lifestyle; medication; conditions; etc. | Explain that it is possible to eat properly (provide tips and links)  Explain how proper diet/nutrition can positively impact health and specifically CV and diabetes | Build positive expectations around the ability to have nutritious meals | Build confidence to seek/make nutritious meals  Provide tips on how to eat healthy |

| **CV-Diabetes– Employee Individual Determinants** | | | | |
| --- | --- | --- | --- | --- |
| **Performance**  **Objectives** | **Knowledge, capability or skill** | **Attitudes, beliefs and values** | **Expectations** | **Self efficacy** |
| Avoid tobacco  Minimize alcohol | Describe how these can impact CV health  Describe resources to be able to stop smoking, where you need to go seek help  (smoking cessation aids available as part of benefit plan??) | Demonstrate that it is okay to seek help | Persuade that it is possible stop smoking or reduce alcohol | Build confidence that you can make changes to these issues |
| Engage in stress/time management  work/life balance | Learn how to better manage time and stress  Describe the available resources to help work/life balance  Wellness Centre information. Some of the wellness programs target stress management | Show that it is possible to have work/life balance  Demonstrate that it is OK to seek help for stress management  Demonstrate the benefits of stress / time management and work/life balance to reducing risk factors for CV and to improve symptoms from CV and diabetes | Persuade that it is possible to reduce stress  Demonstrate that it is okay to create work/life balance in a way that works for you | Build confidence to seek help when work/life stress is totally out of balance |
| seeing family doctor for regular check-up  -monitor BP and blood glucose regularly  check BMI | Learn importance-provide education on need to see doctor to check risk factors  Explain that On-site cardiovascular screening clinics available to all employees at main locations – 15 min with a registered nurse to check CV measures | Establish attitudes beliefs to be proactive and seek preventive measures | Establish expectations that regular check up is necessary |  |
| understanding family history | Learn how family history is a key factor in risk for CV disease.  Annual Wellness Assessment includes family history questions and provides employee with a report that includes information around how this can affect their risk levels |  |  | Build self confidence to ask necessary questions about family history |

| **CV- Diabetes– Employee External Determinants** | | | | | | |
| --- | --- | --- | --- | --- | --- | --- |
| Performance  Objectives | | **Norms**  **and policies** | **Social support** | **Reinforcement** | **Resources** | **Organizational climate** |
| be aware of the signs and symptoms and when they do occur do something about it | | Promote/reinforce the Norm that company will support employee that seeks help.  Include benefits coverage for drugs/treatment for CV/diabetes | Emphasize that if employees seek help they will be supported by their team/manager | Incorporate CV health training into new manager training  Include applicable information in new employee manual/training | Enhance visibility of resources available to employees | Promote an organizational culture where it is okay to seek help and where company will provide necessary support |
| use available resources | Demonstrate how team leaders can provide information/promote resources to team members (e.g. in team meetings)  Other examples: promoting Wellness Centre, smoking cessation aids, other | |  | Demonstrate using data or graphs the utilization of the resources by employees  Host a fair where vendors can provide education and information on services available | Demonstrate that resources are readily accessible; enhance communication and visibility around the resources  Enhance visibility through communicating in common areas (e.g. cafeterias) | Encourage the use of the available resources when needed  Attach the Company brand to philanthropy to support the area of CV health |

| **CV–Diabetes- Employee External Determinants** | | | | | |
| --- | --- | --- | --- | --- | --- |
| **Performance**  **Objectives** | **Norms**  **and policies** | **Social support** | **Reinforcement** | **Resources** | **Organizational climate** |
| medication compliance/appropriate use of medication  be aware of the drugs that you are taking (drug interactions can impact cardiovascular health) | describe that the norm to have resources available on appropriate use of medication and awareness of drug interactions | provide through benefits program to info on medication use | educate employees on the importance of compliance; reinforce that the organization is here to support employees  Provide feedback using HRA | Use flex benefits  and microsite to educate  Encourage use of EAP communications  Include info on Source  Provide information on the importance of medication adherence |  |
| do regular exercise (at least 30 minutes of walking 5 out 7 days per week) | Demonstrate via mentorship by management; role models for exercise and get away from their desk on lunch. Make 20 min of exercise the Norm. | Provide a forum for individuals looking for social support to promote physical activity (i.e. discussions for people to look for group fitness activities) | Use verbal reinforcement and reward positive behaviour  sharing of best practices (e.g. collaboration tool or forum to share what employees/managers are doing)  Managers support for their staff’s healthy behaviours, such as going to the gym at lunch, etc. | Use videos  (e.g 23 1/2 hours)  Wellness programs such as health challenges  Reinforce programs such as Weight Watchers at Work, fitness reimbursements, Wellness Account, on-site gyms | senior leaders demonstrate participating in national wellness day and other wellness programs offered |

| **CV- Diabetes– Employee External Determinants** | | | | | |
| --- | --- | --- | --- | --- | --- |
| Performance  Objectives | **Norms**  **and policies** | **Social support** | **Reinforcement** | **Resources** | **Organizational climate** |
| Adequate sleep | Provide guidelines on avoid sending emails late at night too often, respectful of getting sleep  Describe the recommended sleep requirement (guidelines) |  | Provide feedback using HRA assessment | education around the impact of sleep deprivation and how to ensure adequate sleep  EAP program; sleep webinars as part of wellness program; HRA sleep data for analysis to see if it's going in the right direction over time | emphasize CEO messaging around being a high performance culture and the type of climate we're striving for and how we're going to get there; ensure employees know that a positive culture is important |
| eat properly - reduce fat intake, increase fibre, increase greens reduce refined sugars/alcohol,  monitor diet, reduced processed foods and eating out  weight management | show role modeling; walking the walk; healthy snacks and meals for meetings; where cafeterias exist, ensuring healthy options  discuss about the community norms shifting toward healthy food/drink choices  Provide more option in drinks like juices rather than coffee or pop | Demonstrate peer to peer support around positive eating habits;  Collaboration spaces or forums to share success stories; profile leaders favorite recipes | Demonstrate positive reinforcement by managers on healthy choices by employees | healthy cafeteria food; information on the wellness -part of the Source website e.g. healthy recipes | Senior management demonstrates the importance of proper nutrition and healthy lifestyle and how this is important for a high performance culture |
| Avoid tobacco  Minimize alcohol | Describe that the norm (trends) is to avoid excessive Alcohol and avoid tobacco  Norms around alcohol during socials/limiting use/  Describe policy around allowable expenses for alcohol | Role modeling among peers and provide to help/support for those wanting to quit smoking | Reinforcing smoking policies  Enforce policy smoking around building (second hand smoke) | Demonstrate how EAP can be used to assist employees  Leverage using the Source with other community programs/initiate  Smoking cessation aids potentially covered under benefit plan (?) | An organizational climate that values a positive healthy lifestyle and demonstrates this through the actions of senior management |

| **CV-Diabetes– Employee External Determinants** | | | | | |
| --- | --- | --- | --- | --- | --- |
| Performance  Objectives | **Norms**  **and policies** | **Social support** | **Reinforcement** | **Resources** | **Organizational climate** |
| Engage in stress/time management  work/life balance | Describe flex time schedule to enable employees to accommodate home situation and have time flexibility  Encourage telecommuting and use of flex hours  Encourage discussions between management and employees to regularly check in on stress/time management and work/life balance | Describe how peers encourage and support others who attempt to achieve work-life balance | Directors/ leaders demonstrate work life balance  On The Source have stories/testimonials that may have an effect to model other to achieve the same  Flex time schedules and telecommuting schedules (reinforce that it is an option) | Health and wellness programs that focus on stress mgmt, work life balance, time mgmt  Provide education and opportunities to learn relaxation and stress management eg, lunch and learn  Promote the EAP as resource to get help  Continue to promote wellness programs that target stress mgmt.  Share specialized training information to other departments in the organization (e.g. Market Development conducted time mgmt. training) | Describe a climate by which employee attempt to achieve work-life balance  Describes a climate where work-life balance is valued |
| seeing family doctor for regular check-up  -monitor BP and blood glucose regularly  Check BMI | Describe that is the norm to get check ups to assess risk factors | Establish peer support to encourage regular check ups | Demonstrate that regular check up can prevention mortality and morbidity from CV and/ diabetes  Demonstrate that screening clinics provide information about importance of regular check ups | Demonstrate how available resources can provide education on prevention  Cardiovascular screening clinics | Describes a climate that encourages CV prevention and need to assess risk factors |
| understanding family history | Describe as the norm the understanding that there is a strong link between CV diabetes and family history |  | Describe where screening clinics inquire and act upon high risk family history | Explain resources available to obtain information on family history and CV-Diabetes |  |

**CV-Diabetes– Manager/Supervisor**

| **CV-Diabetes– Manager/Supervisor Individual Determinants** | | | | |
| --- | --- | --- | --- | --- |
| **Performance**  **Objectives** | **Knowledge, capability**  **or skill** | **Attitudes, beliefs and values** | **Expectations** | **Self efficacy** |
| - accountability - awareness of signs and symptoms of employees (e.g. arm pain, shortness of breath, fatigue)  - education/training re signs of cardiovascular disease/diabetes  Focus on function – how is the condition impacting ability to perform the job | Acquire the knowledge and skills to identify employees in distress and available resources for employee to seek help. Include as part of manager training, identifying CV and diabetic health issues and how to deal with them  Accountability to point out when there are gaps in ability to function or change in performance – then strongly encourage to seek medical care | Eliminate negative attitudes toward CV health and diabetes | Expectations that managers will acquire knowledge to deal appropriately with employees with CV health issues and diabetes | Build confidence for managers to identify and take appropriate steps when presented with CV health issues |
| role model (eating properly, exercising, going out for a walk at lunch time, work-life balance, adequate weight) |  | Understand that actions of superiors impact behaviours of employees. | The expectations that managers will act a s role models on healthy living |  |
| -follow processes  -know when to seek help  - minimize ambiguity in employee roles  awareness of resources available to employees (e.g. wellness programs, fitness programs) | Be aware of available resources and what procedures to follow | Encourage attitude that manager and supervisors have an important role in CV/Diabetic health helping to adapt the workplace to the health needs. | Provide training on processes, role and resources available | Demonstrate the confidence to follow processes, when to ask for help and knowledge of available resources |

| **CV-Diabetes– Manager/Supervisor Individual Determinants** | | | | |
| --- | --- | --- | --- | --- |
| **Performance**  **Objectives** | **Knowledge, capability or skill** | **Attitudes, beliefs and values** | **Expectations** | **Self efficacy** |
| -mentorship for prevention  -encourage employees to participate in wellness | Understand that positive (and negative) actions of superiors impact behaviours of employees. | Express belief that participation is important in prevention | Encourage participation in the wellness programs |  |
| - help enable employee to get better | Acquire skills and knowledge to enable employees to seek help they need. |  | Demonstrate willingness  To help | Demonstrate confidence to provide resources to employees to get better (knowing what to provide, when to provide it, etc.) |
| develop positive relationships with employees/subordinates - can lead to open communication, reduced stress, show concern | Communicate ways to recognize employees  Obtain training on how to praise and recognise  Profile on intranet recognition  Provide positive reinforcement. | Understand the importance of recognition | Provide positive recognition | Demonstrates confidence to provide praise and recognition |

| **CV-Diabetes– Manager/Supervisor External Determinants** | | | | | |
| --- | --- | --- | --- | --- | --- |
| Performance  Objectives | **Norms**  **and policies** | **Social support** | **Reinforcement** | **Resources** | **Organizational climate** |
| --learn to identify signs and symptoms and risks  - accountability - awareness of signs and symptoms of employees (e.g. arm pain)  -education/training re signs of cardiovascular disease/diabetes  More in terms of how it’s impacting function and the appropriate action steps to become the norm | Inform that it is the norm (policy) that all managers/supervisors obtain training on best practices  Comparison with other organizations (feedback)  Understanding policy around procedures on how to act when function is impacted due to CV/diabetic illness | Other manager provide support on how to manage CV and diabetic health issues  Encourage support among managers | Senior management recognizes those who have helped others with CV and or diabetic health issues. The Source highlights role  Opportunity to share experiences with other managers on how to act appropriately. | Management provides sufficient resources for education and training  Have refresher training sessions | Demonstrated climate of understanding and open dialogue among managers/supervisors on how to manage effectively when CV/diabetes impact work ability. |
| - better understanding and being active about it - providing modifications, | It is the norm that managers are aware of the resources/processes that will help employees | Shows high support among managers for importance of awareness of resource/processes | Senior management publications/ wellness website/training manuals, The Source all  Provide information on available resources for CV and diabetic health issues for managers | Enhance visibility of resources available to managers/supervisors  Have a go to person for advise/guidance |  |
| -accommodation /flexibility on the part of managers and supervisors | Inform that it is the norm (policy) that all managers/supervisors receive education in CV and diabetes health issues and understand role and actions that should be taken | Shows high support among managers for importance of open communication/ compassion and flexibility | Develop policies to recognize managers who excel in role  Senior management provides positive feedback on their performance |  | Demonstrated climate of understanding and compassion and open dialogue among managers/supervisors and employees |

| **CV-Diabetes– Manager/Supervisor External Determinants** | | | | | |
| --- | --- | --- | --- | --- | --- |
| Performance  Objectives | **Norms**  **and policies** | **Social support** | **Reinforcement** | **Resources** | **Organizational climate** |
| -mentorship for prevention  --encourage employees to participate in wellness  role model (eating properly, exercising, going out for a walk at lunch time, work-life balance, adequate weight) | It is the norm that managers act as role models CV health and participate in wellness initiatives and encourage employees to participate | Manager encourage other managers to be role models and participate in wellness initiatives | Senior management recognized managers who participate in wellness programs and act as role models in CV and diabetes prevention  Support flex time schedules/telecommuting schedules for their employees |  | Construct a climate where everyone wants and has the opportunity to participate in CV wellness initiatives |
| - help enable employee to get resources  awareness of resources available to employees (e.g. wellness programs, fitness programs)  better | Define the norm/policy that managers be trained how to enable employees to help themselves | Managers assist each other on how to enable employees |  | Managers receive the necessary training on how to enable employees | Construct a climate where managers want to help enable employees |
| Provide positive recognition of employees  Develop positive relationships with employees/subordinates - can lead to open communication, reduced stress, show concern | Describe it is the norm to provide positive recognition to employees | Provide positive recognitions among managers | Reinforcement by senior leadership of managers/supervisors who provide positive recognition of employees | Provide necessary training on how to provide positive recognition of employees | Senior management demonstrate positive recognition of managers |

**CV- Diabetes– Co-worker**

| CV-Diabetes– Co-worker Individual Determinants | | | | |
| --- | --- | --- | --- | --- |
| Performance  Objectives | **Knowledge, capability**  **or skill** | **Attitudes, beliefs and values** | **Expectations** | **Self efficacy** |
| be supportive - Work together common goals and objectives-show compassion and concern | Inform co-workers on importance of being supportive and showing compassion | Develop a supportive and caring attitude for workers with CV and diabetic health issues | All co-workers work together and show concern and compassion | Demonstrates confidence in showing compassion and concern |
| help create a positive environment  positive role model (engage in healthy behaviours and wellness programs) | Develop skills to develop and maintain a positive environment | Show how to act into a new way of thinking. Demonstrate that a positive environment is a fun place to work | Expectation that Company is a healthy/positive place to work | Demonstrate that employees have the ability to provide suggestions in creating a positive environment |
| awareness of early signs, identify amongst co-workers (fostering knowledge) | Provide accessible information about CV health and diabetes all employees | Demonstrate the belief that knowing about CV and diabetic health is important | It is expected that all co-worker learn about CV and diabetic health | Demonstrate confidence in learning about CV and diabetic health |

| CV-Diabetes– Co-worker Individual Determinants | | | | |
| --- | --- | --- | --- | --- |
| Performance  Objectives | **Knowledge, capability or skill** | **Attitudes, beliefs and values** | **Expectations** | **Self efficacy** |
| reach out to manager when needed | Understand the importance to reach out to managers when you suspect CV or diabetic health issues among co-workers | Describe the attitude that we all have a role in CV and diabetic health identification and prevention | It is the norm to reach out to managers when suspect CV or diabetic health issues in co-worker | Demonstrate the confidence to reach out to manager when suspect CV issue among co-worker |
| respect confidentiality | Obtain the knowledge around importance of maintaining confidentiality | Demonstrate high level of respect for confidentiality around CV issues of co-workers | It is the norm not to discuss the CV issues of co-workers with other persons |  |

| CV-Diabetes– Co-worker  **External Determinants** | | | | | |
| --- | --- | --- | --- | --- | --- |
| Performance  Objectives | **Norms**  **and policies** | **Social support** | **Reinforcement** | **Resources** | **Organizational climate** |
| support, positive relationships show compassion and concern | Make it the norm that co-worker are supportive and concern for other co-workers with CV or diabetic Health issues | Describe an environment where co-workers support each other | Co-workers are praised for supporting and showing compassion | The Source provides examples and re-enforces a supportive environment. Wellness programs encourage a supportive workplace | Organizational climate is such that a supportive and compassionate workplace is the norm |
| positive role model (engage in healthy behaviours and wellness programs) | Demonstrate how teams who have positive relationships impact the work environment (e.g. socializing with teams; creating opportunities to know each other better) | Demonstrate within the teams that positive relationships are encouraged and well accepted while negative talk/gossip is discouraged within the teams | Recognize/praise positive relationships that are developed by co-workers |  | Demonstrate how senior leaders and their relationships impact the organization positively |
| awareness of early signs, identify amongst co-workers (fostering knowledge) | It is the norm to try to understand/ learn about CV and diabetic health | Co-workers learn /share knowledge about CV/diabetic health | The Source and Wellness initiatives provide opportunity to reinforce key messages about CV/diabetes | Awareness of the resources available to learn more about CV/diabetes | Help create a culture where it is expected that all employees learn about CV/diabetic health |

| CV-Diabetes– Co-worker  **External Determinants** | | | | | |
| --- | --- | --- | --- | --- | --- |
| Performance  Objectives | **Norms**  **and policies** | **Social support** | **Reinforcement** | **Resources** | **Organizational climate** |
| reach out to manager when needed | Make reaching out to managers the norm for co-worker who suspect their co-worker is suffering from CV disease | Co-worker encourage others to reach out to managers | The Source and wellness initiative emphasize take action with CV disease and diabetes | High awareness of where to find information about co-worker role in CV disease and diabetes |  |
| respect confidentiality | Understand policies around confidentiality of health information  It is the norm to respect confidentiality |  | Senior management demonstrates very high degree of importance to maintain confidentiality |  | Organizational climate is such that respect for confidentiality of heath information is a high priority |

| **CV-Diabetes– Senior management Individual Determinants** | | | | |
| --- | --- | --- | --- | --- |
| **Performance**  **Objectives** | **Knowledge, capability or skill** | **Attitudes, beliefs and values** | **Expectations** | **Self efficacy** |
| -invest time and budget  allow for budgeting for programs related to cardiovascular/diabetes disease (providing time and resources to educate) | Increase knowledge on impact of CV/diabetes on presenteeism.  Provide opportunities to education the importance of these health issues at work place. | Opportunity within other areas of the business to enhance attitudes, beliefs and values toward CV/diabetic health for these attitudes to cascade down to employees | Expectation that there will be a movement within the company to provide necessary investment in management/prevention of CV disease/diabetes at workplace |  |
| - role models, walk the talk (diet, exercise, lifestyle, work-life balance) lead by example | Understand important role and learn skills to lead by example | Acquire the attitude that it is your responsibility to lead by example in CV/diabetic health | Those who have experienced CV/diabetic health issues could share their personal story, in order to show their understanding, the impact on them, add a personal touch  (requires creation of opportunity to share health story, similar to the current opportunities to share other stories – e.g. fitness, physical activity | Build self confidence to lead by example in CV health |
| **Performance**  **Objectives** | **Knowledge, capability or skill** | **Attitudes, beliefs and values** | **Expectations** | **Self efficacy** |
| -training to develop positive relationships with employees  training with regards to Cardiovascular Disease | Continue to move toward mandatory education for all managers to enhance knowledge, capability, skill in the area of CV health. | Develop attitude that positive relationship are important and it they have a role to foster/encourage positive relationship with employees | Provide opportunities to facilitate dialogue in this area. E.g. sharing of personal stories  Share stories after training that demonstrates how managers were able to help employees / felt better equipped to support the team due to the training | Share stories after training that demonstrates how managers were able to help employees / felt better equipped to support the team due to the training |
| - foster open communication  - Provide positive recognition of employees  demonstrating accommodation, flexibility | Mandatory training will help enhance knowledge and skills around mental health | Promote the attitude and belief that open communication is important among all levels of employees | Through training, EAP training and support services, and other means of education there will be the expectation that senior management will foster open communication and provide positive recognition | Develop self confidence to be able to foster compassion, open communication and recognition of employees |

**CV-Diabetes– Senior management**

| **CV-Diabetes**-**organization External Determinants** | | | | | |
| --- | --- | --- | --- | --- | --- |
| Performance  Objectives | **Norms**  **and policies** | **Social support** | **Reinforcement** | **Resources** | **Organizational climate** |
| benchmarking - how well is Company doing in comparison with other companies with regards to cardiovascular /diabetic claims?  Integrated data analysis | Continue with benchmarking of CV health indicators |  | Use benchmarking as a means to provide feedback on how we are doing relative to other companies and relative to past company measures  Use the data to help facilitate learning and change throughout the business (managers) | Know where to readily find resources related to benchmarking | Leverage the data most effectively |
| mission statement, main message around health in general  culture - how important is the well-being of our employees  instill/facilitate a health/safety culture  -invest in social capital | Establish that employee health is a top priority  Recognition among senior managers that health is a key component of achieving the goal of being the best performing life insurance company in Canada | Develop a social support throughout the company – from top down around employee health goals and objectives | Establish employee health as a priority and establish metrics to measure success in this area (e.g. health index) | Maintain adequate resources to conduct analysis around performance in employee health | Establish a culture where as part of a definition of high performance culture imbedded in this is the importance of a healthy organization |
| Performance  Objectives | **Norms**  **and policies** | **Social support** | **Reinforcement** | **Resources** | **Organizational climate** |
| communication around expectations re: health/ wellness-make health/wellness the norm | Make wellness part of managers objectives/performance mgmt. plan  Establish a norm for wellness champions to make wellness the norm across the organization |  | Delivering of messages from senior mgmt across to employees through avenues such as videos  Anchor it to the high performance culture, connected to org’n view on org health/our desire to ensure we have a healthy workforce message  Anchor it to other key overarching messages | Provide messages and resources to senior management to clarify expectations and procedures  Ensure that messages are clear for senior management to trickle down to employees | Delivering of messages from senior mgmt across to employees through avenues such as videos  Anchor it to the high performance culture, connected to organization view on org health/our desire to ensure we have a healthy workforce message  Anchor it to other key overarching messages |
| - align policies and procedures to philosophy  policies and procedures related to wellness and prevention | Employee health is a consideration throughout decision making processes within the business  Put decisions through the employee health lens to consider the impact to employee health  Norm around communication between managers and employees |  | There is an understanding that all policies and procedures will be also measured and assessed on how they impact employee health |  | Establish a culture where employee health is paramount in all decisions around policies and procedures |

| **CV-Diabetes**-**organization External Determinants** | | | | | |
| --- | --- | --- | --- | --- | --- |
| Performance  Objectives | **Norms**  **and policies** | **Social support** | **Reinforcement** | **Resources** | **Organizational climate** |
| - provide resources for training managers/ supervisors/ employees  -invest in human capital | Investment in social capital is the norm | There is support throughout organization for training and investment is social capital | Establishes procedures to provide feedback on success of training and ensure all managers/supervisors and senior management receives training | provide necessary resources for training and establishing high social capital | There is a climate that highly values training in health and wellness and investment in social capital |

| **CV-Diabetes–family/partner/community** Individual Determinants | | | | |
| --- | --- | --- | --- | --- |
| Performance  Objectives | **Knowledge, capability**  **or skill** | **Attitudes, beliefs and values** | **Expectations** | **Self efficacy** |
| - supportive environment  being aware of early signs of Cardiovascular Disease | Establish understanding of CV health and role family/partner/community plays in reducing negative impact  Make education/information on CV easily accessible and available  to family/partner/community (role of legislative bodies or government?) | Demonstrate a caring and supportive attitude  Understanding of the serious negative consequences of poor CV health and the role of family in prevention | There is an expectation that everyone needs to be aware of the negative consequences and how they can help in a positive way | Develop the self confidence to acquire and act on the knowledge about CV disease and role of community/partner/friend |
| - recommendation to seek help, not be afraid to seek help  (identification of the signs)  - encourage family members to go to the doctor for check-ups | Acquire the knowledge how to identify CV disease and recommend to seek help. | Establish attitude and belief that we all have responsibility to identify danger signals and recommend they seek help | Know where they should seek help….what resources are available | Develop the self confidence to recommend to seek help |
| - foster awareness about available support and programs (community perspective)  have resources available for people who need help when they need it (community) | Acquire the knowledge of available resources in community | Foster the belief that community supports are accessible and can be helpful (as opposed to providing information only) | Provide the necessary advice is expected  All communities should have resources available and there is a high awareness of this information in community |  |
| bringing awareness and providing resources to all demographics  encourage healthy eating, diet, nutrition at home  - encourage exercise, more walking  - proper use of medication  being aware of and providing support for proper use of medication  - education on signs and symptoms/prevention | Provide the necessary knowledge to all demographics the importance of health living to prevent CV disease and diabetes  Provide the necessary support to encourage appropriate use of medication and to encourage regular testing/screening.  Make available to community information on early signs of CV disease and diabetes and role of prevention | Establish attitude that we all play a role in encouraging and reinforcing healthy living to prevent and adequately treat CV disease and diabetes | Expectation that we all should be role models for healthy living among friends and family | Develop the self confidence to acquire the knowledge and to act to prevent CV disease and diabetes in the home and community |

| **CV-Diabetes- family/partner/community** **External Determinants** | | | | | |
| --- | --- | --- | --- | --- | --- |
| Performance  Objectives | **Norms**  **and policies** | **Social support** | **Reinforcement** | **Resources** | **Organizational climate** |
| - understanding of the impact of CV disease and diabetes on the individual  being aware of early signs of Cardiovascular Disease and recommendation to seek help | It is the norm that the community is educated on CV and diabetes health issues and when to recommend to seek help | There is support among all members of the community of the importance of CV health and its prevention | The community shares positive stories around CV health | Community has the necessary resources to educate members | Establish a climate in the community that CV health is important |
| - foster awareness about available support and programs (community perspective)  have resources available for people who need help when they need it (community)  encourage family members to go to the doctor for check-ups | Establish the norm that everyone should know about available support and resources | Foster support and collaboration between community groups | There is a means to establish awareness through sharing of stories and highlighted in community events | Community provides necessary resources to educate and increase awareness | Community establishes CV health as a priority and provides necessary resources |
| bringing awareness and providing resources to all demographics  encourage healthy eating, diet, nutrition at home  - encourage exercise, more walking  - proper use of medication | The norm is that all members of community can be impacted and that everyone has a role to help  It is the Norm to act as a role model for healthy living | Describe a community where there is support and encouragement for healthy living | Highlight where Community shares positive stories on how the community helps others with CV health issues | Resources are available to educate all segments of the community-ethic groups all ages, most vulnerable |  |

| **CV-Diabetes–health care providers** Individual Determinants | | | | |
| --- | --- | --- | --- | --- |
| Performance  Objectives | **Knowledge, capability**  **or skill** | **Attitudes, beliefs and values** | **Expectations** | **Self efficacy** |
| provide screening for high risk individuals  - provide appropriate interventions  Provide health coaching | Demonstrate/acquire the necessary skills and knowledge  Demonstrate knowledge in evidence-based management of CV disease and diabetes | Acquire the belief that early identification and treatment is key to prevention | Demonstrate skills and knowledge and to act on this knowledge | Demonstrate high confidence to identify and provide necessary treatment/advice |
| stay up-to-date and current with regards to management of CV disease and diabetes | Demonstrate the ability to keep current | Attitude that it is their responsibility to keep up to date | Responsibility to stay current | Enhance confidence that it is possible to stay up-to-date with relevant materials to provide to employees/patients |
| providing teaching and information  in terms of exercise and diet, provide appropriate resources  provide educational resources for people to look for in terms of Cardiovascular disease and risks | Acquire the knowledge and skills to inform employees on self-management strategies | Acquire the belief that dialogue is important to the health care provider/employee relationship | The expectation is that they will discuss, educate and inform employees |  |
| holistic approach (combination of medications, therapy, etc.) commitment to keep working at a treatment plan | To be aware of the various approaches to treatment and be open to employee preferences and a holistic approach. | Acquire the attitude that a holistic approach can be an option for treatment – be open to different possibilities and combinations | It is expected that all treatment options will be discussed and offered to employees | Develop self confidence to use an holistic approach and involve employees in decision process around treatment options |
| follow-up - is the treatment plan working? what adjustments are needed? ongoing case management at the doctor-patient level  regular follow-ups to see if risk factors are under control | Acquire the knowledge around the need to follow-up and monitor progress | Establish the attitude that follow-up and monitoring is important | There is the expectation that there will be follow-up and monitoring and modifying treatment as necessary |  |

| **CV-Diabetes- health care providers** (HCP) **External Determinants** | | | | | |
| --- | --- | --- | --- | --- | --- |
| Performance  Objectives | **Norms**  **and policies** | **Social support** | **Reinforcement** | **Resources** | **Organizational climate** |
| - ability to screen for individuals at high risk and provide appropriate interventions (e.g. referral, medication,  provide screening for high risk individuals | It is the norm to provide appropriate care including screening and treatment | Other HCP provide positive support to provide appropriate care | Describe how others are providing excellent care and using appropriate guidelines  Highlight in community what is high quality care | Ensure sufficient resources are available to provide the appropriate care | There is a climate in the community and among other HCP that appropriate screening and treatment is provided |
| - stay up-to-date and current with regards to mental health trends in Canada | It is the norm to stay up to date on CV care and provide information and teaching on CV health and prevention | Demonstrate positive social pressure in the community to stay up to date | Provide feedback on how others keep up to date | Have available educational material that HCP can use to educate | Describe a climate where it is expected the HCP stay up to date on CV issues…screening and management |
| providing teaching and information  in terms of exercise and diet, provide appropriate resources in terms of Cardiovascular disease and risk prevention | Describe the norm to provide appropriate education and treatment on management and prevention |  |  | Provide resources for HCP to easily pass on to employees | Promote a climate where continuous learning and education is encouraged among HCPs |
| follow-up - is the treatment plan working? what adjustments are needed? ongoing case management at the doctor-patient level  regular follow-ups to see if risk factors are under control | It is the norm to provide the appropriate follow-up and to regularly assess progress/ management effectiveness and outcomes in these individuals  Describe the norm where the HCP, employee and workplace communicate effectively and work together to maximize health of employee | there is positive social support in providing appropriate follow-up | Demonstrate examples where follow-up was essential for improved outcomes | Demonstrate the value of follow-up to the complete care in CV disease | The climate exists where follow-up and re-assessment is expected for quality care |

**Matrix D**

| **cancer– Employee Individual Determinants** | | | | |
| --- | --- | --- | --- | --- |
| **Performance**  **Objectives** | **Knowledge, capability or skill** | **Attitudes, beliefs and values** | **Expectations** | **Self efficacy** |
| be aware of the signs and symptoms and when they do occur do something about it  learn self breast exam  - learn about prevention (colon/breast/skin/prostate/ lung)  Learn about sun protection  - learn/avoid known carcinogens  -learn more about leading a “healthy lifestyle” | Describe or explain what help is available  Explain that there are resources available that will tell you how to seek help (who, where to go)  Learn about prevention, know where to seek information about prevention-  -learn about stress management/relaxation meditation | Explain that by seeking help it will be dealt with in a confidential way  Develop belief that knowing and learning about prevention may save lives | Demonstrate that people that seek help can be helped  Demonstrate that employees what to learn about prevention and what they can do to seek help for themselves | Provide the resources to foster the self-confidence for people to know when and where to seek help when they need it  Demonstrate confidence to seek and implement preventative strategies  Build confidence to seek help when they need it and know where to go to seek help |

**Cancer – Employee**

| use available resources | Describe where they can find available resources when they are suffering from cancer or when they have a family member with cancer  Demonstrate knowledge in prevention and where to seek this knowledge  If individuals are affected, point them to information about what is covered with their benefits, if applicable  Provide information about STD/LTD guidelines | Explain that by using available resources it will be dealt with in a confidential way | Demonstrate that people that seek help can be helped  Demonstrate that employees what to learn about prevention and what they can do to seek help for themselves or their family members | Improve confidence in seeking out available resources  Demonstrate what resources are available – who should people call? |
| --- | --- | --- | --- | --- |
| - take recommended medications and seek appropriate care compliance/appropriate use of medication | Describe the benefits of seeking support, medication/treatment compliance (how to, consequences of not following) e.g. chemotherapy, radiation etc.  Minimum amount of information available on benefits site; information available on wellness centre through health library  -use of evidence based information  -receive guidance from appropriate resources  -research different options and make choices based on evidence | Explain that taking as recommended treatment may impact success  Build attitude for wanting to learn about available resources and advice regarding treatment for cancer | Describe that it is okay to seek support (also applies to attitudes)  Demonstrate that people that seek help can be helped  Demonstrate that employees what to learn about prevention and what they can do to seek help for themselves or their family members | Build confidence to seek support  Build confidence to manage your own compliance  -work is healthy attitude  -build confidence to ask for help from family and friend supports |

| **Cancer– Employee Individual Determinants** | | | | |
| --- | --- | --- | --- | --- |
| **Performance**  **Objectives** | **Knowledge, capability or skill** | **Attitudes, beliefs and values** | **Expectations** | **Self efficacy** |
| do regular exercise (at least 20 minutes of walking 5 out 7 days per week) | Explain how exercise is beneficial for prevention– links on Source to articles for example  Demonstrate how you can find 30 minutes per day to exercise  Explain how exercise can improve your work performance, increase efficiency  Wellness program available at certain times throughout the year – e.g. clinics; health challenges; education sessions/  webinars; online learning programs  learn what exercise is best for your cancer | Explain that it is possible to find 30 min per day to exercise – provide tips and examples  -include family  -make it fun and part of your daily routine/day  Demonstrate the link between regular exercise and decreased risk of cancer  Demonstrate the link between improving symptoms of cancer/providing some relief with light exercise | Build positive expectations around the ability to exercise and the benefits that can be realized.  -find resources to help you  -research and obtain support networks | Build confidence to find 30 minutes during the day to exercise and that it will not impact work performance  -schedule it as part of your day and make it routine |
| Get adequate sleep | Explain how one can achieve adequate sleep (7 hours) and where to find the resources to assist them in getting better sleep  Explain the difference between quantity and quality of sleep and how needs might differ between people | Explain that it is possible to obtain adequate sleep  -build a schedule  -read evidence based materials and information  Demonstrate the link between adequate sleep and decreased risk of cancer  Demonstrate the link between improving symptoms of cancer/providing some relief with adequate sleep  - make a journal of energy levels and mood with amount and quality of sleep | Build positive expectations around the ability to get good sleep  -build into a family routine so that there are supports | Build confidence in ability to utilize strategies to get better sleep |
| eat properly - reduce fat (quantify that we are talking about “bad” fat and not “good” fats intake, increase fibre, increase greens reduce refined sugars/alcohol,  monitor diet, reduced processed foods and eating out  weight management  reduce fat intake, increase fibre, increase greens, | Explain how one can achieve adequate nutrition (low fat, high fibre, low sugar, adequate fruit and vegetable intake, balanced diet, calories) and where to find the resources to assist them in getting better nutrition  Same programs as per above  Wellness Centre available 24/7 to get more information on lifestyle; medication; conditions; etc.  -use of evidence based research and information | Explain that it is possible to eat properly (provide tips and links)  Explain how proper diet/nutrition can positively -impact health  -use of support networks  -build into your routine and schedule  Demonstrate the link between proper nutrition and decreased risk of cancer  Demonstrate the link between improving symptoms of cancer/providing some relief with proper nutrition | Build positive expectations around the ability to have nutritious meal  -build into family routine for support | Build confidence to seek/make nutritious meals  Provide tips on how to eat  Healthy  -get supports to assist you – have confidence to ask for support and help |

| **Cancer– Employee Individual Determinants** | | | | |
| --- | --- | --- | --- | --- |
| **Performance**  **Objectives** | **Knowledge, capability or skill** | **Attitudes, beliefs and values** | **Expectations** | **Self efficacy** |
| Avoid tobacco  Minimize alcohol | Describe how tobacco can increase risk for cancer  Describe how excessive alcohol can cause some cancers(cancers of the esophagus, pharynx, and mouth)  Describe resources to be able to stop smoking, where you need to go seek help  Where can seek help for alcohol addiction  (smoking cessation aids available as part of benefit plan??) | Demonstrate that it is okay to seek help | Persuade that it is possible stop smoking or reduce alcohol | Build confidence that you can make changes to these issues |
| Engage in stress/time management  work/life balance | Learn how to better manage time and stress  Describe the available resources to help work/life balance, ie. EAAP  Wellness Centre information. Some of the wellness programs target stress management  Provide information about Employee and Advisor Assistance Program  Provide information about other wellness programs that help with work/life balance (e.g. Kids & Company daycare) | Show that it is possible to have work/life balance  Demonstrate that it is OK to seek help for stress management  Obtain supports  -de-stigmatize mental health | Persuade that it is possible to reduce stress  Demonstrate that it is okay to create work/life balance in a way that works for you  -learn strategies that work for you a and take time for you | Build confidence to seek help when work/life stress is totally out of balance |
| seeing family doctor for regular check-up/ screening – breast, colon, prostate | Learn importance-provide education on need to see doctor to check risk factors and perform screening tests  Provide information on Physician Search on the Wellness Centre via Canadian HealthCARE Navigation for employees that do not have a family doctor or if they need to find a specialist | Establish attitudes beliefs to be proactive and seek preventive measures  -use evidence based research/information and make choices based on this. | Establish expectations that regular check up and screening is necessary | Build self confidence to ask necessary questions about screenings |
| know about family history and risk | Learn how family history is a key factor in risk for cancer  Annual Wellness Assessment includes family history questions and provides employee with a report that includes information around how this can affect their risk levels |  |  | Build self confidence to ask necessary questions about family history |
| discuss with manager/ supervisor any limitations  -seek support from manager/co-workers/home/community | Build awareness for employees that if their cancer is impacting their abilities that they should seek help/advice from managers | Build attitude and belief that it is OK to seek help | Establish the expectation that it is OK to seek help from managers | Build self confidence to seek help |

| **Cancer - Employee External Determinants** | | | | | | |
| --- | --- | --- | --- | --- | --- | --- |
| Performance  Objectives | | **Norms**  **and policies** | **Social support** | **Reinforcement** | **Resources** | **Organizational climate** |
| be aware of the signs and symptoms and when they do occur do something about it | | Promote/reinforce the Norm that company will support employee that seeks help. | Emphasize that if employees seek help they will be supported by their team/manager | Incorporate cancer training into new manager training  Include applicable information in new employee manual/training | Enhance visibility of resources available to employees (e.g. EAP plus the Wellness Centre information includes support group information) | Promote an organizational culture where it is okay to seek help and where company will provide necessary support |
| use available resources  learn about prevention (colon/breast/skin/prostate/ lung)  Learn about sun protection  - learn/avoid known carcinogens | Demonstrate the norm where team leaders provide information/promote resources to team members (e.g. in team meetings)  It is the norm to provide resource on prevention  Wellness Centre provides resources, like smoking cessation aids, support groups, other | | Promote social support which encourages to seek knowledge and seek help about cancer prevention and when cancer impact work ability | Demonstrate using data or graphs the utilization of the resources by employees | Demonstrate that resources are readily accessible; enhance communication and visibility around the resources  Enhance visibility through communicating in common areas (e.g. cafeterias) or by building into current wellness messaging  Provide sufficient resources to increase knowledge in prevention and where to seek help | Describe a work culture that encourage and promotes the use of the available resources when needed  Promote a culture that prides itself in maximizing the health of it employees |

| **Cancer- Employee External Determinants** | | | | | |
| --- | --- | --- | --- | --- | --- |
| **Performance**  **Objectives** | **Norms**  **and policies** | **Social support** | **Reinforcement** | **Resources** | **Organizational climate** |
| - take recommended medications and seek appropriate care compliance/appropriate use of medication and/or cancer treatments | describe that the norm to have resources available on compliance/appropriate use of medication or cancer treatments (chemo/radiation) | provide through benefits program to info on cancer treatments /medication.  Provide social support during treatment sessions | educate employees on the importance of seeking and using resources on treatment/compliance; reinforce that the organization is here to support employees  Provide feedback using HRA | Use flex benefits  and microsite to educate  Encourage use of EAP communications  Include info on Source  Provide information on cancer treatments. | Promote a culture that prides itself in maximizing the health of it employees |
| do regular exercise (at least 30 minutes of walking 5 out 7 days per week)  for prevention and when suffering from cancer | Demonstrate via mentorship by management; role models for exercise and get away from their desk on lunch. Make 20 min of exercise the Norm.  Describe the norm where you can seek help to find out what exercises are beneficial and how to modify based on individual needs. | Provide a forum for individuals looking for social support to promote physical activity (i.e. discussions for people to look for group fitness activities) | Use verbal reinforcement and reward positive behaviour  sharing of best practices (e.g. collaboration tool or forum to share what employees/managers are doing)  demonstrate how benefits structure rewards those who exercise | Use videos  (e.g 23 1/2 hours)  Wellness programs such as health challenges  Reinforce programs such as Weight Watchers at Work, fitness reimbursements, Wellness Account, on-site gyms  Describe available resources that explains how to get started on exercise program, where to go who to see, or how to o it on their own | senior leaders demonstrate participating in national wellness day and other wellness programs offered  promotes a culture where exercise and fitness is the norm  -work is healthy culture and attitude |

| **Cancer – Employee External Determinants** | | | | | |
| --- | --- | --- | --- | --- | --- |
| Performance  Objectives | **Norms**  **and policies** | **Social support** | **Reinforcement** | **Resources** | **Organizational climate** |
| Adequate sleep | Provide guidelines on avoid sending emails late at night too often, respectful of getting sleep  Describe the recommended sleep requirement (guidelines) |  | Provide feedback using HRA assessment | education around the impact of sleep deprivation and how to ensure adequate sleep  EAP program; sleep webinars as part of wellness program; HRA sleep data for analysis to see if it's going in the right direction over time | emphasize CEO messaging around being a high performance culture and the type of climate we're striving for and how we're going to get there; ensure employees know that a positive culture is important |
| eat properly - reduce fat intake, increase fibre, increase greens reduce refined sugars/alcohol,  monitor diet, reduced processed foods and eating out  weight management | show role modeling; walking the walk; healthy snacks and meals for meetings; where cafeterias exist, ensuring healthy options  discuss about the community norms shifting toward healthy food/drink choices  Provide more option in drinks like juices (?) define juices as this can be as bad as pop I,e. sugar load rather than coffee or pop | Demonstrate peer to peer support around positive eating habits;  collaboration spaces or forums to share success stories; profile leaders favorite recipes | Demonstrate positive reinforcement by managers on healthy choices by employees | healthy cafeteria food; information on the wellness -part of the Source website e.g. healthy recipes | Senior management demonstrates the importance of proper nutrition and healthy lifestyle and how this is important for a high performance culture  -support healthy eating options, lead by example through senior leaders  -have an organizational climate that only supports healthy eating in buildings i.e. don’t have vending machines with pop, chocolate, chips, bad choices and have cafeterias provide healthy options |
| Avoid tobacco  Minimize alcohol | Describe that the norm (trends) is to avoid excessive Alcohol and avoid tobacco  Norms around alcohol during socials/limiting use/  Describe policy around allowable expenses for alcohol | Role modeling among peers and provide to help/support for those wanting to quit smoking | Reinforcing smoking policies  Enforce policy smoking around building (second hand smoke) | Demonstrate how EAP can be used to assist employees  Leverage using the Source with other community programs/initiate  Smoking cessation aids potentially covered under benefit plan (?)  Use of Benefits site for health information and drug information supports | An organizational climate that values a positive healthy lifestyle and demonstrates this through the actions of senior management |

| **Cancer – Employee External Determinants** | | | | | |
| --- | --- | --- | --- | --- | --- |
| Performance  Objectives | **Norms**  **and policies** | **Social support** | **Reinforcement** | **Resources** | **Organizational climate** |
| Engage in stress/time management  work/life balance | Describe flex time schedule to enable employees to accommodate home situation and have time flexibility  Encourage telecommuting and use of flex hours  Encourage discussions between management and employees to regularly check in on stress/time management and work/life balance | Describe how peers encourage and support others who attempt to achieve work-life balance | Directors/ leaders demonstrate work life balance  On The Source have stories/testimonials that may have an effect to model other to achieve the same | Health and wellness programs that focus on stress mgmt, work life balance, time mgmt  Provide education and opportunities to learn relaxation and stress management eg, lunch and learn  Promote the EAP as resource to get individualized help  Continue to promote wellness programs that target stress mgmt.  Share specialized training information to other departments in the organization (e.g. Market Development conducted time mgmt. training) | Describe a climate that promotes work-life balance  Describes a climate where work-life balance is valued  -Healthy workplace strategies including vision, mission, objectives and goals |
| seeing family doctor for regular check-up/ screening – breast, colon, prostate | Describe that is the norm to get check ups to assess risk factors, for screening, monitoring and management  -provide or encourage use of evidence based practice | Establish peer support to encourage regular check ups/screening | Demonstrate through education (website, EAP, posters, other) that regular check ups/ screening can prevent mortality and reduce morbidity from cancer  Provide regular messaging about cancer screening to employees | Demonstrate how available resources can provide education on screening/prevention  Provide information on  recommended screening tests (for breast cancer, colon, prostate, cervical etc. | Describes a climate that encourages cancer prevention and need to assess risk factors. |
| know about family history and risk | Describe as the norm the understanding that there is a strong link between family history and many cancers |  | Describe where screening clinics inquire and act upon high risk family history  Provide regular messaging about cancer screening to employees | Explain resources available to obtain information on family history and cancer | Describes a climate that encourages cancer prevention and need to assess risk factors. |
| discuss with manager/ supervisor any limitations  -seek support from manager/co-workers/home/community | Describe the norm to seek help from managers when cancer impact work ability  Explain that it is policy that information about personal health conditions will be kept strictly confidential. | Demonstrate through social support among peers that it is important to seek and communicate limitations with managers | Describe through various communications (narratives/stories) about how seeking help/communication with managers was a positive experience | Describe how at various points of interaction employees (interviews, employee handbook, The Source where employee receive messaging about communicating limitations with managers | Describe a culture that promotes open and confidential communication with managers when there are limitations in work ability. |

| **Cancer – Manager/Supervisor Individual Determinants** | | | | |
| --- | --- | --- | --- | --- |
| **Performance**  **Objectives** | **Knowledge, capability**  **or skill** | **Attitudes, beliefs and values** | **Expectations** | **Self efficacy** |
| accountability -  - education/training how to modify work to suit limitations  Focus on function – how is the condition impacting ability to perform the job  provide support/empathy/compassion and concern  recognize early signs…weight loss, change in behaviours, malaise, excessive time off work etc. | Acquire the knowledge and skills to identify employees who may be in distress and what resources are available for employee to seek help.  Learn to show compassion/concern/empathy  Know how to effectively modify work to suit limitations  Ensure accountability to point out when there are gaps in ability to function or change in performance  Know when to encourage to seek medical care | Eliminate negative attitudes toward individuals who are limited in their work ability due to cancer  Develop a positive attitude about interacting and role with employees with cancer | Expectations that managers will acquire knowledge to deal appropriately with employees with cancer | Build confidence for managers to identify and take appropriate steps when presented with employee with cancer who is functionally limited. |
| role model (eating properly, exercising, going out for a walk at lunch time, work-life balance, adequate weight) | Understand that actions of superiors impact behaviours of employees. |  | The expectations that managers will act as role models on healthy living |  |
| -follow processes  -know when to seek help  - minimize ambiguity in employee roles  awareness of resources available to employees (e.g. wellness programs, fitness programs)  -mentorship for prevention | Be aware of available resources and what procedures to follow  Provide training on processes, role and resources available | Encourage attitude that manager and supervisors have an important role in cancer and its prevention helping to adapt the workplace to the health needs. | Reinforce the expectation that managers should know what processes to follow and be aware of available resources | Demonstrate the confidence to follow processes, when to ask for help and knowledge of available resources |

| **Cancer – Manager/Supervisor Individual Determinants** | | | | |
| --- | --- | --- | --- | --- |
| **Performance**  **Objectives** | **Knowledge, capability or skill** | **Attitudes, beliefs and values** | **Expectations** | **Self efficacy** |
| -encourage employees to participate in wellness | Understand that positive (and negative) actions of superiors impact behaviours of employees. | Express belief that participation is important in prevention | Encourage participation in the wellness programs  Describe an expectation that managers participate in wellness initiative  Wellness as an agenda meeting item in some of the leaders meetings held by department heads in order to discuss expectations and answer questions | Develop confidence in ability to encourage employees to participate in wellness initiatives.  -agenda in workplace meetings  -lead by example |
| - help enable employee to get better | Acquire skills and knowledge to enable employees to seek help they need.  -participate in wellness  -use key stakeholders | =Understand the importance of a manager/supervisor’s positive attitudes influence  -develop EQ | Demonstrate willingness  To help  -provide resources  -lead by example | Demonstrate confidence to provide resources to employees to get better (knowing what to provide, when to provide it, etc.) |
| develop positive relationships with employees/subordinates - can lead to open communication, reduced stress, show concern | Communicate ways to recognize employees  Obtain training on how to praise and recognise  Profile on intranet recognition  Provide positive reinforcement. | Understand the importance of recognition  -regular communication with employee in difference format and venues i.e. one on one meetings, follow up with email discussions, etc, positive relationships | Provide positive recognition – use methods available through the source for positive recognition (i.e. sending a VIP) | Demonstrates confidence to provide praise and recognition |

| **Cancer – Manager/Supervisor External Determinants** | | | | | |
| --- | --- | --- | --- | --- | --- |
| Performance  Objectives | **Norms**  **and policies** | **Social support** | **Reinforcement** | **Resources** | **Organizational climate** |
| accountability -  education/training how to modify work to suit limitations  Focus on function – how is the condition impacting ability to perform the job  provide support/empathy/compassion and concern  recognize early signs…weight loss, change in behaviours, malaise, excessive time off work etc | Inform that it is the norm (policy) that all managers/supervisors obtain training on best practices on how to deal with functional limitations  Comparison with other organizations (feedback)  Understanding policy around procedures on how to act when function is impacted due to cancer  Describe as the norm that managers identify ill health at the workplace that impacts functional abilities and how to effectively address the limitations in a compassionate caring way | Other manager provide support on how to manage functionally impacting health issues  Encourage support among managers  -use key stakeholders | Senior management recognizes those who have helped others with cancer. The Source highlights role  Opportunity to share experiences with other managers on how to act appropriately.  Senior management express the importance of effectively addressing health limiting issues in a compassionate and caring way | Management provides sufficient resources for education and training  Have refresher training sessions | Demonstrated climate of understanding and open dialogue among managers/supervisors on how to manage effectively when cancer impact work ability.  -Healthy Workplace vision and mission statements, goals and objectives |
| - better understanding and being active about it - providing modifications, | It is the norm that managers are aware of the resources/processes that will help employees | Shows high support among managers for importance of awareness of resource/processes | Senior management/ company publications/ wellness website/training manuals, The Source all  Provide information on available resources for cancer and its prevention for managers | Enhance visibility of resources available to managers/supervisors  Have a go to person for advise/guidance | Healthy Workplace vision and mission statements, goals and objectives |
| -accommodation /flexibility on the part of managers and supervisors | Inform that it is the norm (policy) that all managers/supervisors receive education in cancer and work and understand role and actions that should be taken  -people leader training and practices | Shows high support among managers for importance of open communication/ compassion and flexibility | Develop policies to recognize managers who excel in role  Senior management provides positive feedback on their performance |  | Demonstrated climate of understanding and compassion and open dialogue among managers/supervisors and employees  Healthy Workplace vision and mission statements, goals and objectives  Demonstrate a climate of flexibility and accommodation for special circumstances |

| **Cancer – Manager/Supervisor External Determinants** | | | | | |
| --- | --- | --- | --- | --- | --- |
| Performance  Objectives | **Norms**  **and policies** | **Social support** | **Reinforcement** | **Resources** | **Organizational climate** |
| role model (eating properly, exercising, going out for a walk at lunch time, work-life balance, adequate weight)  -encourage employees to participate in wellness | It is the norm that managers act as role models in health promotion and participate in wellness initiatives and encourage employees to participate | Manager encourage other managers to be role models and participate in wellness initiatives | Senior management recognized managers who participate in wellness programs and act as role models in health promotion/prevention  Leaders talk about with managers in department leadership meetings to bring to the forefront | Managers know where to access resources to help be a role model and participate in wellness initiatives (The Sources, wellness portal, etc.) | Construct a climate where everyone wants to participate in wellness initiatives  Healthy Workplace vision and mission statements, goals and objectives |
| -follow processes  -know when to seek help  - minimize ambiguity in employee roles  awareness of resources available to employees (e.g. wellness programs, fitness programs)  -mentorship for prevention | Define the norm/policy that managers be trained how to enable employees to help themselves | Managers assist each other on how to enable employees |  | Managers receive the necessary training on how to enable employees | Construct a climate where managers want to help enable employees |
| Provide positive recognition of employees  develop positive relationships with employees/subordinates - can lead to open communication, reduced stress, show concern | Describe it is the norm to provide positive recognition to employees | Managers develop positive relationships with other managers and share knowledge on how to be positive with employees as well | Reinforcement by senior leadership of managers/supervisors who provide positive recognition of employees | Provide necessary training on how to provide positive recognition of employees | Senior management demonstrate positive recognition of managers |

**Cancer – Co-worker**

| **Cancer** – Co-worker Individual Determinants | | | | |
| --- | --- | --- | --- | --- |
| Performance  Objectives | **Knowledge, capability**  **or skill** | **Attitudes, beliefs and values** | **Expectations** | **Self efficacy** |
| be supportive - Work together common goals and objectives-show compassion and concern | Inform co-workers on importance of being supportive and showing compassion | Develop a supportive and caring attitude for workers with cancer | All co-workers work together and show concern and compassion | Demonstrates confidence in showing compassion and concern |
| help create a positive environment  positive role model (engage in healthy behaviours and wellness programs) | Develop skills to develop and maintain a positive environment | Show how to act into a new way of thinking. Demonstrate that a positive environment is a fun place to work | It is expected that all co-workers contribute towards a positive environment and are positive role models for each other | Demonstrate that employees have the ability to provide suggestions in creating a positive environment |
| awareness of early signs, identify amongst co-workers (fostering knowledge) and know when they should contact managers  acquire and share knowledge about prevention | Provide accessible information about cancer and work and prevention  Acquire the knowledge when to seek help | Demonstrate the belief that knowing about cancer and how it can impact work ability is important  Describe the belief that knowledge and action in cancer prevention is important | It is expected that all co-worker learn about reducing risk of cancer and support those who have cancer at work | Demonstrate confidence in learning about cancer and work and prevention |

| **Cancer-** Co-worker Individual Determinants | | | | |
| --- | --- | --- | --- | --- |
| Performance  Objectives | **Knowledge, capability or skill** | **Attitudes, beliefs and values** | **Expectations** | **Self efficacy** |
| reach out to manager when needed | Understand the importance to reach out to managers when co-workers is in distress  Know when to reach out. | Describe the attitude that we all have a role in helping/supporting workers with cancer and knowing about it prevention | It is the norm to reach out to managers when suspect CV or diabetic health issues in co-worker | Demonstrate the confidence to reach out to manager when co-worker is in distress |
| respect confidentiality | Obtain the knowledge around importance of maintaining confidentiality | Demonstrate high level of respect for confidentiality of co-workers with cancer | It is expected not to discuss the health issues of co-workers with other persons |  |

| **Cancer** – Co-worker  **External Determinants** | | | | | |
| --- | --- | --- | --- | --- | --- |
| Performance  Objectives | **Norms**  **and policies** | **Social support** | **Reinforcement** | **Resources** | **Organizational climate** |
| be supportive - Work together common goals and objectives-show compassion and concern | Make it the norm that co-worker are supportive and concern for other co-workers with cancer | Describe an environment where co-workers support each other | Co-workers are praised for supporting and showing compassion | The Source provides examples and re-enforces a supportive environment. Wellness programs encourage a supportive workplace | Organizational climate is such that a supportive and compassionate workplace is the norm |
| help create a positive environment  positive role model (engage in healthy behaviours and wellness programs) | Demonstrate how teams who have positive relationships impact the work environment (e.g. socializing with teams; creating opportunities to know each other better) | Demonstrate within the teams that positive relationships are encouraged and well accepted while negative talk/gossip is discouraged within the teams | Recognize/praise positive relationships that are developed by co-workers |  | Demonstrate how senior leaders and their relationships impact the organization positively |
| awareness of early signs, identify amongst co-workers (fostering knowledge) and know when they should contact managers  acquire and share knowledge about prevention | It is the norm to try to understand/ learn about cancer and impact on work | Co-workers learn /share knowledge about cancer and work and share knowledge about prevention | The Source and Wellness initiatives provide opportunity to reinforce key messages about cancer prevention and management | Awareness of the resources available to learn more about cancer | Help create a culture where it is expected that all employees learn about cancer and its prevention |

| **Cancer** – Co-worker  **External Determinants** | | | | | |
| --- | --- | --- | --- | --- | --- |
| Performance  Objectives | **Norms**  **and policies** | **Social support** | **Reinforcement** | **Resources** | **Organizational climate** |
| reach out to manager when needed | Make reaching out to managers the norm for co-worker who suspect their co-worker is suffering from CV disease | Co-worker encourage others to reach out to managers | The Source and wellness initiative emphasize take action with CV disease and diabetes | High awareness of where to find information about co-worker role in CV disease and diabetes | Create an organizational climate where employees are comfortable with reaching out to managers – open door policies |
| respect confidentiality | Understand policies around confidentiality of health information  It is the norm to respect confidentiality |  | Senior management demonstrates very high degree of importance to maintain confidentiality |  | Organizational climate is such that respect for confidentiality of heath information is a high priority |

| **Cancer – Senior management Individual Determinants** | | | | |
| --- | --- | --- | --- | --- |
| **Performance**  **Objectives** | **Knowledge, capability or skill** | **Attitudes, beliefs and values** | **Expectations** | **Self efficacy** |
| -invest time and budget  allow for budgeting for programs related to education on cancer and related health issues (providing time and resources to educate)  how manager need to effective manage as related to work ability | Increase knowledge on impact of cancer on work  Provide opportunities to education the importance of these health issues at work place. | Opportunity within other areas of the business to enhance attitudes, beliefs and values toward cancer and work and for these attitudes to cascade down to employees | Expectation that there will be a movement within the company to provide necessary investment in management/prevention of cancer at workplace |  |
| - role models, walk the talk (diet, exercise, lifestyle, work-life balance) lead by example | Understand important role and learn skills to lead by example  Demonstrate knowledge and action towards healthy living | Acquire the attitude that it is your responsibility to lead by example in health promotion | Those who have experienced cancer could share their personal story, in order to show their understanding, the impact on them, add a personal touch  (requires creation of opportunity to share health story, similar to the current opportunities to share other stories – e.g. fitness, physical activity | Build self confidence to lead by example |
| **Performance**  **Objectives** | **Knowledge, capability or skill** | **Attitudes, beliefs and values** | **Expectations** | **Self efficacy** |
| - training to develop positive relationships with employees  - foster open communication  - encourage flexibility among mangers/supervisors | Continue to move toward mandatory education for all managers to enhance knowledge, capability, skill in the area of work and  Health.  Acquire knowledge on how to interact in a positive way with managers/employees | Develop attitude that positive relationship are important and it they have a role to foster/encourage positive relationship with employees | Provide opportunities to facilitate dialogue in this area. E.g. sharing of personal stories  Share stories after training that demonstrates how managers were able to help employees / felt better equipped to support the team due to the training | Share stories after training that demonstrates how managers were able to help employees / felt better equipped to support the team due to the training |
| - foster open communication  - Provide positive recognition of employees  demonstrating accommodation, flexibility | Mandatory training will help enhance knowledge and skills around work and health of employees |  | Through training, EAP training and support services, and other means of education there will be the expectation that senior management will foster open communication and provide positive recognition | Develop self confidence to be able to foster compassion, open communication and recognition of employees |

**Cancer – Organization**

| **Cancer** -**organization External Determinants** | | | | | |
| --- | --- | --- | --- | --- | --- |
| Performance  Objectives | **Norms**  **and policies** | **Social support** | **Reinforcement** | **Resources** | **Organizational climate** |
| benchmarking - how well is Company doing in comparison with other companies with regards to cancer?  Integrated data analysis | Continue with benchmarking of cancer and related health indicators |  | Use benchmarking as a means to provide feedback on how we are doing relative to other companies and relative to past company measures  Use the data to help facilitate learning and change throughout the business (managers) |  | Leverage the data most effectively |
| mission statement, main message around health in general  culture - how important is the well-being of our employees  instill/facilitate a health/safety culture  -invest in social capital | Establish that employee health is a top priority  Recognition among senior managers that health is a key component of achieving the goal of being the best performing life insurance company in Canada | Develop a social support throughout the company – from top down around employee health goals and objectives | Establish employee health as a priority and establish metrics to measure success in this area (e.g. health index) | Maintain adequate resources to conduct analysis around performance in employee health | Establish a culture where as part of a definition of high performance culture imbedded in this is the importance of a healthy organization |
| Performance  Objectives | **Norms**  **and policies** | **Social support** | **Reinforcement** | **Resources** | **Organizational climate** |
| communication around expectations re: health/ wellness-make health/wellness the norm | Make wellness part of managers objectives/performance mgmt. plan |  | Delivering of messages from senior mgmt across to employees through avenues such as videos  Anchor it to the high performance culture, connected to organization n view on organization health/our desire to ensure we have a healthy workforce message  Anchor it to other key overarching messages | Provide messages and resources to senior management to clarify expectations and procedures  Ensure that messages are clear for senior management to trickle down to employees | Delivering of messages from senior mgmt across to employees through avenues such as videos  Anchor it to the high performance culture, connected to organization view on org health/our desire to ensure we have a healthy workforce message  Anchor it to other key overarching messages |
| - align policies and procedures to philosophy  policies and procedures related to wellness and prevention | Employee health is a consideration throughout decision making processes within the business  Put decisions through the employee health lens to consider the impact to employee health  Norm around communication between managers and employees |  | There is an understanding that all policies and procedures will be also measured and assessed on how they impact employee health |  | Establish a culture where employee health is paramount in all decisions around policies and procedures |

| **Cancer** -**organization External Determinants** | | | | | |
| --- | --- | --- | --- | --- | --- |
| Performance  Objectives | **Norms**  **and policies** | **Social support** | **Reinforcement** | **Resources** | **Organizational climate** |
| - provide resources for training managers/ supervisors/ employees  -invest in human capital and prevention | Investment in social capital is the norm | There is support throughout organization for training and investment is social capital | Establishes procedures to provide feedback on success of training and ensure all managers/supervisors and senior management receives training | Provide necessary resources for training and establishing high social capital | There is a climate that highly values training in health and wellness and investment in social capital |

| **Cancer– family/partner/community** Individual Determinants | | | | |
| --- | --- | --- | --- | --- |
| Performance  Objectives | **Knowledge, capability**  **or skill** | **Attitudes, beliefs and values** | **Expectations** | **Self efficacy** |
| - supportive environment  being aware of early signs of Cancer (unexplained weight loss, malaise, fatigue)  support/assistance in treatment…chemo/radiation/medication treatment and be aware of side-effects  - awareness of the role and potential consequences of narcotic use for pain | Establish understanding of role of family/partner /community plays in reducing negative impact of cancer.  Describe a mechanism where information on resources options are made available to family/partner community including information on treatment side effects and sign and symptoms.  Describe an mechanism where company can help family find resources to help worker with cancer and information on cancer prevention | Demonstrate a caring and supportive attitude  Promoting a belief that family/partner/community play important role in cancer by providing support being aware of treatment side effects encouraging treatment compliance | There is an expectation that family/partner/community can make a difference in outcomes | Develop the self confidence to acquire and act on the knowledge about role of community/partner/friend in cancer.  Demonstrating the knowledge where to go for information and assistance (e.g. wellness centre where support group information is held) |
| - recommendation to seek help, not be afraid to seek help  - encourage family members to go for cancer screening | Acquire the knowledge how to identify potential signs and symptoms of cancer and when to recommend to seek help. | Establish attitude and belief that we all have responsibility to identify danger signals and recommend they seek help and to encourage preventative measures | Know where they should seek help….what resources are available | Develop the self confidence to recommend to seek help |
| - aware of Canadian Cancer Society resources  have resources available for people who need help when they need it (community)  be aware of community /work resources/support groups to assist in management | Acquire the knowledge of available resources in community | Establish the attitude and belief that community resources can be helpful | Provide the necessary advice is expected  All communities should have resources available and there is a high awareness of this information in community | Develop the self confidence to research community resources / support groups |
| - positive healthy role models (eating, exercise, weight, attitudes, relationships, work-life balance)  make healthy lifestyle the norm  provide emotional support and encouragement  - understand the impact cancer can have on the individual | Provide the necessary knowledge to act as role model in the prevention of cancer  Provide the necessary support to encourage regular testing/screening. | Establish attitude that we all play a role in encouraging and reinforcing healthy living to prevent and adequately treat cancer | Expectation that we all should be role models for healthy living among friends and family | Develop the self confidence to acquire the knowledge and to act to prevent cancer and assist in supporting cancer management at home |

| **Cancer - family/partner/community** **External Determinants** | | | | | |
| --- | --- | --- | --- | --- | --- |
| Performance  Objectives | **Norms**  **and policies** | **Social support** | **Reinforcement** | **Resources** | **Organizational climate** |
| - supportive environment  being aware of early signs of Cancer (unexplained weight loss, malaise, fatigue)  support/assistance in treatment…chemo/radiation/medication treatment and be aware of side-effects  - awareness of the role and potential consequences of narcotic use for pain | It is the norm that the community is educated on cancer prevention and how to provide support, knowing the importance and encouraging screening.  Describe the norm where family members know where to seek resources to assist and provide support and knowing about potential side effects of treatment | There is support among all members of the community of the importance of cancer prevention and the need to provide support. | The community shares positive stories around cancer | Community has the necessary resources to educate members | Establish a climate in the community that preventing and supports those with cancer is important |
| have resources available for people who need help when they need it (community)  - recommendation to seek help, not be afraid to seek help  - encourage family members to go for cancer screening | Establish the norm that everyone should know about available support and resources, importance of cancer screening, and who is at higher risk |  | There is a means to establish awareness through sharing of stories and highlighted in community events | Community provides necessary resources to educate and increase awareness | Community establishes cancer as a priority and provides necessary resources |
| - positive healthy role models (eating, exercise, weight, attitudes, relationships, work-life balance)  make healthy lifestyle the norm  provide emotional support and encouragement  - understand the impact cancer can have on the individual | The norm is that all members of community can be impacted and that everyone has a role to help  It is the Norm to act as a role model for healthy living | Describe a community where there is support and encouragement for healthy living | Highlight where Community shares positive stories on how the community helps others with cancer | Resources are available to educate all segments of the community-ethic groups all ages, most vulnerable |  |

| **Cancer –health care providers** Individual Determinants | | | | |
| --- | --- | --- | --- | --- |
| Performance  Objectives | **Knowledge, capability**  **or skill** | **Attitudes, beliefs and values** | **Expectations** | **Self efficacy** |
| provide screening for high risk individuals  - provide appropriate interventions  Show compassion and concern | Demonstrate/acquire the necessary skills and knowledge  Demonstrate knowledge in evidence-based management of cancer treatment and prevention | Acquire the belief that early identification and treatment is key to prevention | Demonstrate skills and knowledge and to act on this knowledge | Demonstrate high confidence to identify and provide necessary treatment/advice |
| stay up-to-date and current with regards to management and prevention of cancer  act as positive role model | Demonstrate the ability to keep current | Attitude that it is their responsibility to keep up to date  Demonstrate the belief that they should act as role model for healthy living | Responsibility to stay current |  |
| providing education on prevention  in terms of exercise and diet, provide appropriate resources/side effects/treatment compliance | Acquire the knowledge and skills to inform employees on self-management strategies | Acquire the belief that dialogue is important to the health care provider/employee relationship | The expectation is that they will discuss, educate and inform employees | Demonstrate confidence to provide education on prevention and appropriate resources, side effects, and advice on treatment compliance |
| follow-up - is the treatment plan working? what adjustments are needed? ongoing case management at the doctor-patient level | Acquire the knowledge around the need to follow-up and monitor progress | Establish the attitude that follow-up and monitoring is important | There is the expectation that there will be follow-up and monitoring and modifying treatment as necessary |  |
| communication with workplace on work modification | Acquire the knowledge how to effectively communicate work limitations with workplace  Understand necessity to get worker consent to communicate with workplace. | Describe an attitude where communication with workplace about limitations is part of appropriate management | Describe the expectation that specific work limitation/modification be communicated with the workplace | Develop confidence to communicate with workplace. |

| **Cancer- health care providers** (HCP) **External Determinants** | | | | | |
| --- | --- | --- | --- | --- | --- |
| Performance  Objectives | **Norms**  **and policies** | **Social support** | **Reinforcement** | **Resources** | **Organizational climate** |
| - ability to screen for individuals at high risk and provide appropriate interventions (e.g. referral, medication,  provide screening for high risk individuals  show compassion and concern | It is the norm to provide appropriate care including screening and treatment  It is the norm to treat with compassion and concern | Other HCP provide positive support to provide appropriate care | Describe how others are providing excellent care and using appropriate guidelines  Highlight in community what is high quality care | Ensure sufficient resources are available to provide the appropriate care | There is a climate in the community and among other HCP that appropriate screening and treatment is provided |
| - stay up-to-date and current  Act as positive role model | It is the norm to stay up to date on cancer care and provide information and teaching on cancer management and prevention | Demonstrate positive social pressure in the community to stay up to date | Provide feedback on how others keep up to date | Have available educational material that HCP can use to educate | Describe a climate where it is expected the HCP stay up to date on cancer…screening and management |
| providing teaching and information  in terms of exercise and diet, provide appropriate resources in terms of cancer risk prevention | Describe the norm to provide appropriate education and treatment on management and prevention |  | Demonstrate examples where positive lifestyle habits can impact cancer risk prevention | Provide resources for HCP to easily pass on to employees |  |
| follow-up - is the treatment plan working? what adjustments are needed? ongoing case management at the doctor-patient level  regular follow-ups to see if risk factors are under control | It is the norm to provide the appropriate follow-up and to regularly assess progress/ management effectiveness and outcomes in these individuals | there is positive social support in providing appropriate follow-up | Demonstrate examples where follow-up was essential for improved outcomes | Demonstrate the value of follow-up to the complete care in cancer | The climate exists where follow-up and re-assessment is expected for quality care |
| communication with workplace on work modification | Describe the norm where the HCP, employee and workplace communicate effectively and work together to maximize health of employee |  |  | There are sufficient resource to encourage HCP-workplace communication |  |

**Matrix E**

| **Flu– Employee Individual Determinants** | | | | |
| --- | --- | --- | --- | --- |
| **Performance**  **Objectives** | **Knowledge, capability or skill** | **Attitudes, beliefs and values** | **Expectations** | **Self efficacy** |
| -get the flu shot; understand the risks and benefits of the flu shot - decide whether the flu shot is right for them  use available resources to learn about the flu shot | Explain that there are resources available that will provide you with information about the flu and the flu shot  Learn about prevention, know where to seek information about prevention- | Develop the attitude that getting the flu shot is a individual decisions and should be aware of the risks and benefits | Describe the expectation that employees make informed decisions about the flu shot | Provide the resources to foster the self-confidence for people to make the right decision for them  Demonstrate confidence to seek and implement preventative strategies |

**Flu– Employee**

| do regular exercise (at least 30 minutes of walking 5 out 7 days per week) | Explain how exercise is beneficial for prevention– links on Source to articles for example  Demonstrate how you can find 30 minutes per day to exercise  Describe how regular exercise, proper diet, sleep can minimize risk of flu. | | Explain that it is possible to find 30 min per day to exercise – provide tips and examples | Build positive expectations around the ability to exercise and the benefits that can be realized. | | Build confidence to find 30 minutes during the day to exercise and that it will not impact work performance |
| --- | --- | --- | --- | --- | --- | --- |
| Get adequate sleep | Explain how one can achieve adequate sleep (7 hours) and where to find the resources to assist them in getting better sleep  Explain the difference between quantity and quality of sleep and how needs might differ between people | | Explain that it is possible to obtain adequate sleep | Build positive expectations around the ability to get good sleep | | Build confidence in ability to utilize strategies to get better sleep |
| eat properly - reduce fat intake, increase fibre, increase greens reduce refined sugars/alcohol,  monitor diet, reduced processed foods | Explain how one can achieve adequate nutrition (low fat, high fibre, low sugar, calories) and where to find the resources to assist them in getting better nutrition  Wellness Centre available 24/7 to get more information on lifestyle; medication; conditions; etc. | | Explain that it is possible to eat properly (provide tips and links)  Explain how proper diet/nutrition can positively impact health | Build positive expectations around the ability to have nutritious meals | | Build confidence to seek/make nutritious meals  Provide tips on how to eat healthy |
| - wash hands  -know how to adequately wash hands work/home  - good sanitary practices at work and at home  - cough/sneeze into elbow not hands  -use hand sanitizers regularly | Acquire knowledge on hand washing. Know where to find necessary information | Instill the belief that hand washing /use of sanitizers and cough into elbow reduces spread of the flu | | | Develop expectation that everyone practices preventive measures |  |
| limit exposure to others who are sick  - if sick, limit exposure (stay home if they are sick) |  |  | | |  |  |

| **Flu- Employee External Determinants** | | | | | |
| --- | --- | --- | --- | --- | --- |
| Performance  Objectives | **Norms**  **and policies** | **Social support** | **Reinforcement** | **Resources** | **Organizational climate** |
| - understand the risks and benefits of the flu shot - decide whether the flu shot is right for them  use available resources to learn about the flu shot  Continue to offer flu shot clinics pn-site | Describe the norm where people weigh risk and benefit of flu shot and know where to get this information | Describe peer support for informed decision making | Describe various forms of communication about flu shot and importance in controlling disease | Enhance visibility of resources available to employees | Promote an organizational culture where awareness in flu prevention is important |
| do regular exercise (at least 20 minutes of walking 5 out 7 days per week) | Demonstrate via mentorship by management; role models for exercise and get away from their desk on lunch. Make 20 min of exercise the Norm.  Describe the norm where you can seek help to find out what exercises are beneficial and how to modify based on individual needs. | Provide a forum for individuals looking for social support to promote physical activity (i.e. discussions for people to look for group fitness activities) | Use verbal reinforcement and reward positive behaviour  sharing of best practices (e.g. collaboration tool or forum to share what employees/managers are doing)  demonstrate how benefits structure rewards those who exercise | Use videos  (e.g 23 1/2 hours)  Wellness programs such as health challenges  Reinforce programs such as Weight Watchers at Work, fitness reimbursements, Wellness Account, on-site gyms  Describe available resources that explains how to get started on exercise program, where to go who to see, or how to o it on their own | senior leaders demonstrate participating in national wellness day and other wellness programs offered  promotes a culture where exercise and fitness is the norm |

| **Flu– Employee External Determinants** | | | | | |
| --- | --- | --- | --- | --- | --- |
| Performance  Objectives | **Norms**  **and policies** | **Social support** | **Reinforcement** | **Resources** | **Organizational climate** |
| Adequate sleep | Provide guidelines on avoid sending emails late at night too often, respectful of getting sleep  Describe the recommended sleep requirement (guidelines) |  | Provide feedback using HRA assessment | education around the impact of sleep deprivation and how to ensure adequate sleep  EAP program; sleep webinars as part of wellness program; HRA sleep data for analysis to see if it's going in the right direction over time | emphasize CEO messaging around being a high performance culture and the type of climate we're striving for and how we're going to get there; ensure employees know that a positive culture is important |
| eat properly - reduce fat intake, increase fibre, increase greens reduce refined sugars/alcohol,  monitor diet, reduced processed foods | show role modeling; walking the walk; healthy snacks and meals for meetings; where cafeterias exist, ensuring healthy options  discuss about the community norms shifting toward healthy food/drink choices  Provide more option in drinks like juices rather than coffee or pop | Demonstrate peer to peer support around positive eating habits;  collaboration spaces or forums to share success stories; profile leaders favorite recipes | Demonstrate positive reinforcement by managers on healthy choices by employees | healthy cafeteria food; information on the wellness -part of the Source website e.g. healthy recipes | Senior management demonstrates the importance of proper nutrition and healthy lifestyle and how this is important for a high performance culture |

| - wash hands  -know how to adequately wash hands work/home  - good sanitary practices at work and at home  - cough/sneeze into elbow not hands  -use hand sanitizers regularly | Describe appropriate hand washing and sanitary practices as the norm |  | Various publication/communications provide information on sanitary and preventive practices |  |  |
| --- | --- | --- | --- | --- | --- |
| limit exposure to others who are sick  - if sick, limit exposure (stay home if they are sick) | It is the norm to stay home if have the flu |  |  | There are resources available to work from home when have flu |  |

| **Flu– Manager/Supervisor Individual Determinants** | | | | |
| --- | --- | --- | --- | --- |
| **Performance**  **Objectives** | **Knowledge, capability**  **or skill** | **Attitudes, beliefs and values** | **Expectations** | **Self efficacy** |
| .support staff if they need to stay home if they are sick  manage work, clients  - understand the HR absence policies and processes and share with the team - make sure everyone is aware of the steps they need to take if they are sick (who to call, how often to check in) | Understand the consequences of people coming to work with the flu  Acquire adequate training and education in policy and best practices |  | Describe the expectations that if have flu should stay home |  |
| role model (don’t come into work when sick; eating properly, exercising, going out for a walk at lunch time, work-life balance, adequate weight)  mentorship for prevention | Understand that actions of superiors impact behaviours of employees. |  | The expectations that managers will act a s role models on healthy living |  |
| awareness of resources available to employees (e.g. wellness programs, fitness programs) for information on prevention and to make informed decision about getting the flu shot | Be aware of available resources and what procedures to follow | Encourage attitude that manager and supervisors have an important role in flu prevention | Provide training on processes, role and resources available | Demonstrate the confidence to follow processes, when to ask for help and knowledge of available resources |

| -encourage employees to participate in wellness | Understand that positive (and negative) actions of superiors impact behaviours of employees. | Express belief that participation is important in prevention | Encourage participation in the wellness programs  Describe an expectation that managers participate in wellness initiative; talk about in leaders meetings | Develop confidence in ability to encourage employees to participate in wellness initiatives. |
| --- | --- | --- | --- | --- |
| develop positive relationships with employees/subordinates - can lead to open communication, reduced stress, show concern | Communicate ways to recognize employees  Obtain training on how to praise and recognise  Profile on intranet recognition  Provide positive reinforcement. | Understand the importance of recognition | Provide positive recognition | Demonstrates confidence to provide praise and recognition |

| **Flu– Manager/Supervisor External Determinants** | | | | | | | | |
| --- | --- | --- | --- | --- | --- | --- | --- | --- |
| Performance  Objectives | | **Norms**  **and policies** | **Social support** | | **Reinforcement** | **Resources** | | **Organizational climate** |
| -support staff if they need to stay home if they are sick  manage work, clients  - understand the HR absence policies and processes and share with the team - make sure everyone is aware of the steps they need to take if they are sick (who to call, how often to check in) | | Inform that it is the norm (policy) that all managers/supervisors obtain training on best practices on how to deal flu  Comparison with other organizations (feedback) | Other manager provide support on how to manage issues around the flu  Encourage support among managers | | Opportunity to share experiences with other managers on how to act appropriately.  Senior management express the importance of effectively addressing flu prevention | Management provides sufficient resources for education and training  Have refresher training sessions | | Demonstrated climate of understanding and open dialogue among managers/supervisors on how to manage the flu |
| role model (eating properly, exercising, going out for a walk at lunch time, work-life balance, adequate weight)  mentorship for prevention | | It is the norm that managers act as role models in health promotion and participate in wellness initiatives and encourage employees to participate | Manager encourage other managers to be role models and participate in wellness initiatives | | Senior management recognized managers who participate in wellness programs and act as role models in health promotion/prevention |  | | Construct a climate where everyone wants to participate in wellness initiatives |
| awareness of resources available to employees (e.g. wellness programs, fitness programs) for information on prevention and to make informed decision about getting the flu shot | | Describe the norm that mangers are aware of resources to make appropriate decisions  And how to maintain health lifestyle |  | |  |  | |  |
| Provide positive recognition of employees  develop positive relationships with employees/subordinates - can lead to open communication, reduced stress, show concern | Describe it is the norm to provide positive recognition to employees | | |  | Reinforcement by senior leadership of managers/supervisors who provide positive recognition of employees | Provide necessary training on how to provide positive recognition of employees | Senior management demonstrate positive recognition of managers | |

**Flu– Co-worker**

| Flu– Co-worker Individual Determinants | | | | | | | |  |
| --- | --- | --- | --- | --- | --- | --- | --- | --- |
| Performance  Objectives | **Knowledge, capability**  **or skill** | | **Attitudes, beliefs and values** | | **Expectations** | **Self efficacy** | |  |
| - understand the risks and benefits of the flu shot - decide whether the flu shot is right for them | Understand and share knowledge about available that will provide information about the flu and the flu shot  Learn and share about prevention, know where to seek information about prevention- | | Develop and share the attitude that getting the flu shot is a individual decisions and should be aware of the risks and benefits | | Describe the expectation that employees make informed decisions about the flu shot | Provide the resources to foster the self-confidence for people to make the right decision for them  Demonstrate confidence to seek and implement preventative strategies | |  |
| positive role model (engage in healthy behaviours and wellness programs) and sanitary practices; stay home when sicko | Develop skills to develop and maintain a healthy environment | |  | |  |  | |  |
| do regular exercise (at least 20 minutes of walking 5 out 7 days per week)-act as role model | Explain how exercise is beneficial for prevention– links on Source to articles for example  Demonstrate how you can find 30 minutes per day to exercise  Describe how regular exercise, proper diet, sleep can minimize risk of flu. | Explain that it is possible to find 30 min per day to exercise – provide tips and examples | | Build positive expectations around the ability to exercise and the benefits that can be realized. | | Build confidence to find 30 minutes during the day to exercise and that it will not impact work performance | | |
| Get adequate sleep- role model | Explain how one can achieve adequate sleep (7 hours) and where to find the resources to assist them in getting better sleep  Explain the difference between quantity and quality of sleep and how needs might differ between people | Explain that it is possible to obtain adequate sleep | | Build positive expectations around the ability to get good sleep | | Build confidence in ability to utilize strategies to get better sleep | | |
| eat properly - reduce fat intake, increase fibre, increase greens reduce refined sugars/alcohol,  monitor diet, reduced processed foods –act as role model | Explain how one can achieve adequate nutrition (low fat, high fibre, low sugar, calories) and where to find the resources to assist them in getting better nutrition  Wellness Centre available 24/7 to get more information on lifestyle; medication; conditions; etc. | Explain that it is possible to eat properly (provide tips and links)  Explain how proper diet/nutrition can positively impact health | | Build positive expectations around the ability to have nutritious meals | | Build confidence to seek/make nutritious meals  Provide tips on how to eat healthy | | |
| - wash hands  -know how to adequately wash hands work/home  - good sanitary practices at work and at home  - cough/sneeze into elbow not hands  -use hand sanitizers regularly | Acquire and share knowledge on hand washing. Know where to find necessary information | Instill and share the belief that hand washing /use of sanitizers and cough into elbow reduces spread of the flu | | Develop expectation that everyone practices preventive measures | | |  | |
| limit exposure to others who are sick  - if sick, limit exposure (stay home if they are sick) |  |  | |  | | |  | |

| Flu– Co-worker  **External Determinants** | | | | | | | |
| --- | --- | --- | --- | --- | --- | --- | --- |
| Performance  Objectives | **Norms**  **and policies** | **Social support** | **Reinforcement** | | **Resources** | **Organizational climate** | |
| -; understand the risks and benefits of the flu shot - decide whether the flu shot is right for them | Describe the norm where people weigh risk and benefit of flu shot and know where to get this information | Describe peer support for informed decision making | Describe various forms of communication about flu shot and importance in controlling disease | | Enhance visibility of resources available to employees | Promote an organizational culture where awareness in flu prevention is important | |
| positive role model (engage in healthy behaviours, exercise, sleep, diet and wellness programs) and sanitary practices | show role modeling; walking the walk; healthy snacks and meals for meetings; where cafeterias exist, ensuring healthy options  discuss about the community norms shifting toward healthy food/drink choices | Demonstrate peer to peer support around positive eating habits;  collaboration spaces or forums to share success stories; profile leaders favorite recipes | Demonstrate positive reinforcement by managers on healthy choices by employees | | healthy cafeteria food; information on the wellness -part of the Source website e.g. healthy recipes | Senior management demonstrates the importance of proper nutrition and healthy lifestyle and how this is important for a high performance culture | |
| limit exposure to others who are sick  - if sick, limit exposure (stay home if they are sick) |  |  |  |  | | |  |

**Flu– Organization**

| **Flu- organization External Determinants** | | | | | |
| --- | --- | --- | --- | --- | --- |
| Performance  Objectives | **Norms**  **and policies** | **Social support** | **Reinforcement** | **Resources** | **Organizational climate** |
| benchmarking - how well is Company doing in comparison with other companies with regards to flu  Integrated data analysis | Continue with benchmarking of flu and related health indicators |  | Use benchmarking as a means to provide feedback on how we are doing relative to other companies and relative to past company measures  Use the data to help facilitate learning and change throughout the business (managers) |  | Leverage the data most effectively |
| mission statement, main message around health in general  culture - how important is the well-being of our employees  instill/facilitate a health/safety culture  -invest in social capital | Establish that employee health is a top priority  Recognition among senior managers that health is a key component of achieving the goal of being the best performing life insurance company in Canada | Develop a social support throughout the company – from top down around employee health goals and objectives | Establish employee health as a priority and establish metrics to measure success in this area (e.g. health index) | Maintain adequate resources to conduct analysis around performance in employee health | Establish a culture where as part of a definition of high performance culture imbedded in this is the importance of a healthy organization |
| having the right practices and policies in place to support short-term absences due to illness (e.g. offering personal illness days to employees, ensuring this benefit aligns with employees' needs)  - resources |  |  |  |  |  |
| communication around expectations re: health/ wellness-make health/wellness the norm | Make wellness part of managers objectives/performance mgmt. plan |  | Delivering of messages from senior mgmt across to employees through avenues such as videos  Anchor it to the high performance culture, connected to organization view on org health/our desire to ensure we have a healthy workforce message  Anchor it to other key overarching messages | Provide messages and resources to senior management to clarify expectations and procedures  Ensure that messages are clear for senior management to trickle down to employees | Delivering of messages from senior mgmt across to employees through avenues such as videos  Anchor it to the high performance culture, connected to organization view on org health/our desire to ensure we have a healthy workforce message  Anchor it to other key overarching messages |
| having the right practices and policies in place to support short-term absences due to illness (e.g. offering personal illness days to employees, ensuring this benefit aligns with employees' needs)  - resources |  |  |  |  |  |

| **Performance**  **Objectives** | **Knowledge, capability or skill** | **Attitudes, beliefs and values** | **Expectations** | **Self efficacy** |
| --- | --- | --- | --- | --- |
| -invest time and budget  allow for budgeting for programs related to  education and awareness about prevention - providing information  - e.g. biometric screening clinics, flu prevention | Increase knowledge on impact of flu on work  Provide opportunities to education the importance of these health issues at work place. |  |  |  |
| - role models, walk the talk (diet, exercise, lifestyle, work-life balance) lead by example, sanitary practices | Understand important role and learn skills to lead by example  Demonstrate knowledge and action towards healthy living | Acquire the attitude that it is your responsibility to lead by example in health promotion | Those who have experienced cancer could share their personal story, in order to show their understanding, the impact on them, add a personal touch  (requires creation of opportunity to share health story, similar to the current opportunities to share other stories – e.g. fitness, physical activity | Build self confidence to lead by example |
| make sure everyone knows the plan- business continuity (e.g. flu pandemic) - having a plan in place for what the organization should do  - awareness of daycare programs |  |  |  |  |

| Flu-**organization External Determinants** | | | | | |
| --- | --- | --- | --- | --- | --- |
| Performance  Objectives | **Norms**  **and policies** | **Social support** | **Reinforcement** | **Resources** | **Organizational climate** |
| provide the flu shot clinic for those who are interested in getting the shot |  |  |  |  |  |
| business continuity (e.g. flu pandemic) - having a plan in place for what the organization should do |  |  |  |  |  |

| **Flu-family/partner/community** Individual Determinants | | | | |
| --- | --- | --- | --- | --- |
| Performance  Objectives | **Knowledge, capability**  **or skill** | **Attitudes, beliefs and values** | **Expectations** | **Self efficacy** |
| same as above (employee and co-worker) |  |  |  |  |
| - providing support to people that want to take the shot |  |  |  |  |
| - provide information, education  - provide support for the people that need to stay home  - hygiene education in terms of prevention |  |  |  |  |
| - positive healthy role models (eating, exercise, weight, attitudes, relationships, work-life balance)  make healthy lifestyle the norm | Provide the necessary knowledge to act as role model in the prevention of flu  Provide the necessary support to encourage regular testing/screening. | Establish attitude that we all play a role in encouraging and reinforcing healthy living to prevent the flu | Expectation that we all should be role models for healthy living among friends and family | Develop the self confidence to acquire the knowledge and to act to prevent flu |

| **Flu- family/partner/community** **External Determinants** | | | | | |
| --- | --- | --- | --- | --- | --- |
| Performance  Objectives | **Norms**  **and policies** | **Social support** | **Reinforcement** | **Resources** | **Organizational climate** |
| same as above (employee and co-worker) |  |  |  |  |  |
| - providing support to people that want to take the shot | It is the norm that the company offers flu shot clinics on company time each year |  |  |  |  |
| - provide information, education  - provide support for the people that need to stay home  - hygiene education in terms of prevention | Norm to provide education or links to education sites on the flu and flu shot when promoting flu shot clinics |  |  |  |  |
| - positive healthy role models (eating, exercise, weight, attitudes, relationships, work-life balance)  make healthy lifestyle the norm |  |  |  |  |  |

| **Flu–health care providers** Individual Determinants | | | | |
| --- | --- | --- | --- | --- |
| Performance  Objectives | **Knowledge, capability**  **or skill** | **Attitudes, beliefs and values** | **Expectations** | **Self efficacy** |
| - education, especially for high-risk people  - providing the flu shot for those that want it  - education in the area of prevention |  |  |  |  |
| dispensing medication that is needed |  |  |  |  |
| be clear to the employee on restrictions and limitations (e.g. when they can return to the office)  communication via employee to the workplace - between health care provider's recommendations to the workplace, via the employee |  |  |  |  |
| provide people with information regarding alternatives to medicine |  |  |  |  |
| EAP - provide education, daycare issues, family care (consequences, what to do) |  |  |  |  |

| **Flu- health care providers** (HCP) **External Determinants** | | | | | | |
| --- | --- | --- | --- | --- | --- | --- |
| Performance  Objectives | | **Norms**  **and policies** | **Social support** | **Reinforcement** | **Resources** | **Organizational climate** |
| - providing support to people that want to take the shot | |  |  |  |  |  |
| - stay up-to-date and current  Act as positive role model  positive healthy role models (eating, exercise, weight, attitudes, relationships, work-life balance)  make healthy lifestyle the norm | | It is the norm to stay up to date on flu care and provide information and teaching on flu management and prevention | Demonstrate positive social pressure in the community to stay up to date | Provide feedback on how others keep up to date | Have available educational material that HCP can use to educate | Describe a climate where it is expected the HCP stay up to date on flu management and prevention |
| providing teaching and information  in terms of exercise and diet, provide appropriate resources in terms of flu prevention | | Describe the norm to provide appropriate education and treatment on management and prevention |  |  | Provide resources for HCP to easily pass on to employees |  |
| be clear to the employee on restrictions and limitations (e.g. when they can return to the office)  communication via employee to the workplace - between health care provider's recommendations to the workplace, via the employee |  |  |  |  |  |  |
| communication with workplace on work modification | Describe the norm where the HCP, employee and workplace communicate effectively and work together to maximize health of employee |  |  | There are sufficient resource to encourage HCP-workplace communication |  |  |

EAP= Employee Assistance Program, CEO= Chief Executive Officer, Mgmt= Management, MH= Mental Health, MSK= Musculoskeletal, CV= Cardiovascular,

HCP= Health Care Professional, EAAP= Employee Assistance and Advice Program
