# Supplementary material for: Healthy and productive workers: using intervention mapping to design a workplace health promotion and wellness program to improve presenteeism
Source: BMC Public Health. 2016 Nov 25;16:1190. doi: 10.1186/s12889-016-3843-x (PMC5123329; doi:10.1186/s12889-016-3843-x)
Supplement: Additional file 2: — Appendix C.Step 3. Translate learn and change objectives into to practical strategies. Mental Health and General. (DOCX 78 kb) [file 12889_2016_3843_MOESM2_ESM.docx]

Additional file 2: Appendix C. Step 3. Translate learn and change objectives into to practical strategies. Mental Health and General

| **Objectives –Column one**  *What needs to be done / what needs to be changed?* | **Methods- Column two**  *How can these objectives be accomplished?* | |  |
| --- | --- | --- | --- |
|  | **Best practices and initial ideas** | **Big ideas** There are no wrong answers – think creatively!  *What would be the ideal activities to implement if there were no limitations (e.g. structural, organizational, financial, etc.)?* | **How can we implement this in our workplace and when could we implement?**  **If it’s already being done today, how can we improve it?**  **(Practical applications)** |
| **Employee** | | |  |
| Inform employees at all levels about mental health issues  Explaining/de-mystify stigma around mental health  To build confidence to be able to discuss mental health problems  Demonstrate that leadership will be supportive of mental health issues  Know when to seek help and find resources | - Use Structured multi-pronged educational interventions - Use The Source website - Include applicable information in new employee manual/ training - Use Lunch and learns- use guest speakers -psychologist/ behavioural therapist etc. - Add de-stigmatization training for employees - Have Leadership communicate with employees regularly (scheduled and anticipated) using multi-media and personal stories-clear simple caring messages - Use Modeling-identify and train opinion leaders (respected peers) to act as health ambassadors-role models - Use Health Coaching/nurse or lay health worker, have onsite health centre - Use The Source website to provide interactive cognitively based self help programs, chat room and access to consult with professional/personal coach available for tailoring program | Hold focus groups for employee groups that talk about mental health and receive input from employees around de-stigmatizing mental health (could be run by outside group rather  Have on-demand mental health resources available such as an online depression centre geared towards low to moderate risk employees. | Part of program: extend manager training to employees  Focus groups – opportunities to incorporate as advanced/progressive element of training program   - Could include managers and employees - Could pilot as an add-on to get more learnings - Health consultants could facilitate using their Mental Health First Aid training however; may eliminate some of the sensitivities by heaving outside body facilitate   Source website – use to tell people’s stories; use to change behaviours/attitudes; leverage the polls to include questions around mental health to increase awareness  Leverage the mental health website; social networking may be part of this portal as a later component  Leverage Idea Share to start the dialogue  Design a mental health communication strategy  Identify leader with a personal story (could be internal leader, or could be an external leader/athlete/person in the public eye)  Promote education sessions (lunch n learns) that focus on mental health through EAP program  Identify opinion leaders to become team ambassadors/role model (stretch goal) –ask who the individuals are that they respect; conduct sessions with them and send them back into the community |
| **Objectives –Column one**  *What needs to be done / what needs to be changed?* | **Methods- Column two**  *How can these objectives be accomplished?* | |  |
|  | **Best practices and initial ideas** | **Big ideas** There are no wrong answers – think creatively!  *What would be the ideal activities to implement if there were no limitations (e.g. structural, organizational, financial, etc.)?* |  |
| Enhance visibility of resources available to employees | - Use The Source - Use General emails - Use Posters/ employee manual | Internal video outlining commitment and reiterating resources available | Include in communication plan – leader video could be one of the tactics in the plan (provides ‘permission’ and encouragement to employees) |
| Explain the role of positive relationships in (mental) health  Change attitudes around the impact of gossip at the workplace | - Have Employee training - Have Employee training - Have regular “grunt” sessions with employees where they can express challenges/feedback and come up with own solutions-empowerment –team building-goal setting-participatory problem solving | - Employee focus groups - Team meeting agenda items | Integrate health and wellness messaging into new employee on experience  Integrate health and wellness/absence mgmt./mental health resources into new manager  Incorporate a health and wellness ambassador into new employee (e.g. touch base over first 6 weeks); could be calendar driven – highlight wellness programs currently happening or coming up  Find the people who are already coaches from a business perspective  Managers to provide opportunities for ‘grunt’ sessions among their employees – solutions come within the group  New people leader training – Leading through influence; leading for success |
| **Objectives –Column one**  *What needs to be done / what needs to be changed?* | **Methods- Column two**  *How can these objectives be accomplished?* | |  |
|  | **Best practices and initial ideas** | **Big ideas** There are no wrong answers – think creatively!  *What would be the ideal activities to implement if there were no limitations (e.g. structural, organizational, financial, etc.)?* | **How can we implement this in our workplace and when could we implement?**  **If it’s already being done today, how can we improve it?**  **(Practical applications)** |
| Improve confidence in participating in social networks  Improve confidence and social support for participating in wellness initiatives | - Use Employee communication; personal and interactive, blogs, testimonials, stories, social networking- socialize importance of health - Leverage technology IT - Use reward program, team health team competition/challenges, online health games, - Provide reward statement online - Use time off reward |  | - The Source website as the ideal spot for posting communications, potential to use it more - Options to include comments under articles - Collaboration site – ask employees to share successes, stories and tips; included ability for other employees to comment back and forth) - have a wellness-landing page on website, how do we create additional communication to share their wellness stories?  1. How do we get people to build confidence to participate in these activities? 2. Once they’re in the site, how do we get them to sign up and participate in initiatives and social media?  - Incentives – currently linking participation and engagement in health management programs and wellness programs with benefits plan - Consideration of timelines and peak participation of wellness initiatives – share real time experiences of initiatives, adds engagement   How can we get people to participate throughout the year? (Conversations about health issues)   - For example, distribute monthly themed articles, allow for conversations to begin (e.g., flu, holidays, Healthy Workplace Month) - E.g., every Tuesday is Recipe Day, people look forward to the website update, try, comment, engage in conversation |
| **Objectives –Column one**  *What needs to be done / what needs to be changed?* | **Methods- Column two**  *How can these objectives be accomplished?* | |  |
|  | **Best practices and initial ideas** | **Big ideas** There are no wrong answers – think creatively!  *What would be the ideal activities to implement if there were no limitations (e.g. structural, organizational, financial, etc.)?* | **How can we implement this in our workplace and when could we implement?**  **If it’s already being done today, how can we improve it?**  **(Practical applications)** |
| **Managers** | | |  |
| De-stigmatization training for managers  Expectations that managers will participate in training and deal appropriate with employees with mental health issues and have confidence to do so and have Awareness of processes, role and resources  Demonstrate and praise individuals who discuss their mental health issues  Demonstrate caring and compassion and openness and enable employees | - Incorporate mental health training into new manager training - Have a go to person for advice/guidance - Share stories after training that demonstrates how managers were able to help employees / felt better equipped to support the team due to the training | - Mental health training as part of performance mgmt. process - Include more role-playing in manager training in order to practice discussions with employees/teams - Reiteration of commitment and of manager responsibilities through emails/videos from organization leaders - Highlight where managers can go for help/advice on having conversations on the topic | - Mental health training incorporated into new manager training. - Managers would go to the Consultant for advice/consultant - Opportunity to include a forum for managers to share their thoughts after training, currently not done today. Could become an online forum or portal. - Looked into idea of monthly webinar for training participants to share their ideas, coming together, challenges and successes, create a network where people feel comfortable talking - Communication between senior management and managers – mental health as priority |
| Enhance visibility of resources available to employees | - The Source - General emails - Posters | See previous pages | Manager training, need to find a way to make training mandatory   - Video created around EAAP services Another creative way to share information about programs and services available by prompted new calls to EAAP provider and conversations, targeted employees and to managers (orientation program to EAAP) - Welcome phone call to new employees, welcome call materials to include wording around EAAP program - Employee reviews of their manager during performance review time - Where employees express concerns around their manager (i.e. how they are treated, manager lack of concern for work life balance, negative talk, etc.) be able to take action and have policies around this |
| **Objectives –Column one**  *What needs to be done / what needs to be changed?* | **Methods- Column two**  *How can these objectives be accomplished?* | |  |
|  | **Best practices and initial ideas** | **Big ideas** There are no wrong answers – think creatively!  *What would be the ideal activities to implement if there were no limitations (e.g. structural, organizational, financial, etc.)?* | **How can we implement this in our workplace and when could we implement?**  **If it’s already being done today, how can we improve it?**  **(Practical applications)** |
| Encourage participation in the wellness programs  Managers encourage other managers to be role models and participate in wellness initiatives | - Profile on intranet, recognition | - Part of manager performance management process - One goal on performance evaluations could be related to employee wellness programs | - Build out strategy with multiple touchpoints - By including wellness participation become the ‘stick’ versus the ‘carrot’ approach? – may be an opportunity to do both - E.g., asking “how many wellness programs have you participated in this year?” puts it on the radar, by asking it must be important, as opposed to being punitive. Start the conversation going, build up awareness |
| Inform that it is the norm (policy) that all managers/supervisors obtain training on best practices and on of available resources | - Publications/ wellness website/training manuals, The Source all provide information on available resources for MH for managers | Incorporate into performance management process | May not be able to make it a policy, but we can begin conversations |
| Senior management recognizes those who have helped others with MH (reinforcement)  Provide necessary training on how to provide positive recognition of employees | - The Source highlights role - Identify and train manager opinion leaders as health ambassadors –role models | Share stories at leader meetings | Brainstorm ‘how do you get people to share their stories?’ (send an email request, for example)   - Can we create a panel of individuals (leaders) to profile their experiences? Thought leadership team - How can we create another platform for everyone to share and collaborate on their stories?   Ask the question |
| Shows high social support among managers for importance of awareness of resource/processes for MH and provide positive recognition |  | Create manager focus groups as opportunities to ask questions, work through given scenarios in order to role play how to handle | Related to manager training, forum and follow up  Role play was included initially but was stripped out due to length of session |

Mental Health and General

| **Objectives –Column one**  *What needs to be done / what needs to be changed?* | **Methods- Column two**  *How can these objectives be accomplished?* | |  |
| --- | --- | --- | --- |
|  | **Best practices and initial ideas** | **Big ideas** There are no wrong answers – think creatively!  *What would be the ideal activities to implement if there were no limitations (e.g. structural, organizational, financial, etc.)?* | **How can we implement this in our workplace and when could we implement?**  **If it’s already being done today, how can we improve it?**  **(Practical applications)** |
| **Co-workers** | | |  |
| Inform co-workers on importance of being supportive and showing compassion  Provide accessible information about MH to all employees  Describe the attitude that we all have a role in MH identification and prevention  Make it the norm that co-worker are supportive and show compassion and concern for other co-workers with MH  Recognize/praise positive relationships that are developed by co-workers  Awareness of the resources available to learn more about MH | - The Source provides examples and reinforces a supportive environment. Wellness programs encourage a supportive workplace - Provide a link to social network within The Source with chat room - Develop buddy system for social support | Guest speakers who have been there  Mental health ambassadors…from within the organization (those who are suffering, have suffered, or have dealt with mental health issues among loved ones)  Stories and testimonials – multi-media  Mental health ‘blitzes’ to share practical tips and scripts. Posters, desk drops, email.  Stories and testimonials – multi-media  Marketing/Communications campaign. Survey. Quiz or contest to drive awareness with decent prizes. | See above |
| **Objectives –Column one**  *What needs to be done / what needs to be changed?* | **Methods- Column two**  *How can these objectives be accomplished?* | |  |
|  | **Best practices and initial ideas** | **Big ideas** There are no wrong answers – think creatively!  *What would be the ideal activities to implement if there were no limitations (e.g. structural, organizational, financial, etc.)?* | **How can we implement this in our workplace and when could we implement?**  **If it’s already being done today, how can we improve it?**  **(Practical applications)** |
| **Senior management** | | |  |
| Increase sensitivity around mental health within Group Benefits and throughout other business units  Continue to move toward mandatory training for all managers to enhance knowledge, capability, skill in the area of mental health | - Make mental health training mandatory (currently in place for Group Benefits) for all business units and as an on-demand resource - Provide management training on how to promote health and productivity (mandatory) - Have regular “grunt” sessions with managers where they can express challenges and come up with own solutions - Have face-to-face meeting with managers - Provide clear job description for managers and other employees –realistic job expectations - Brand Wellness Program-give it status and recognition - Develop and use social media tools to consistently promote wellness - Initiate team building exercises-trust games | Make it real by finding an executive who is willing to share their story.  Leadership ‘forum’ on how to deal with mental health challenges within teams…how to walk the talk. Recognize there is some rich experience within our walls and the opportunity to learn from each other!  Make stronger connections between as a provider of innovative benefits & wellness solutions and our own internal programs as an opportunity to showcase best practices. | How do we get an ambassador at the executive level? (a key element for success). Internally we need to find ways to influence people at the top.  “Grunt” sessions not happening formally currently   - Within Development there are ‘coffee chats’ with VP for people to talk about what is on their minds, implement solutions that target work-life balance - Branding currently happening, top of the field in wellness - Currently have team building exercises for leaders - Work underway to lead to a stronger value proposition to address connections between company the provider and the client |
| Acquire the attitude that it is your responsibility to lead by example in MH- role model  Develop attitude that positive relationship are important and it they have a role to foster/encourage positive relationship open communication with employees | - Those who have experienced mental health issues share their personal story, in order to show their understanding, the impact on them, add a personal touch (requires creation of opportunity to share mental health story, similar to the current opportunities to share other stories – e.g. fitness, physical activity - Use multi-media to communicate on a regular structured format | Senior leadership to model the desired behaviours. Recognize all of the above is needed to ensure they have the knowledge, skills, tools and support. Where do they turn when unsure?  Incorporate health objectives into performance plans.  Provide leaders with turn-key videos, presentations, scripts and guest-speaker opportunities to be leveraged in team meetings and planning sessions. Remove lack of time and lack of understanding as barriers. | Face-to-face contact recommended  Tools are already in place, a story is needed for distribution |
| **Objectives –Column one**  *What needs to be done / what needs to be changed?* | **Methods- Column two**  *How can these objectives be accomplished?* | |  |
|  | **Best practices and initial ideas** | **Big ideas** There are no wrong answers – think creatively!  *What would be the ideal activities to implement if there were no limitations (e.g. structural, organizational, financial, etc.)?* | **How can we implement this in our workplace and when could we implement?**  **If it’s already being done today, how can we improve it?**  **(Practical applications)** |
| **Organization** | | |  |
| benchmarking of mental health indicators  Leverage the data most effectively | - Audit work environment and health promotion programs to align with culture-use reward, recognition | Develop a health scorecard – determine audiences and frequency for distribution as well as process for identifying themes/opportunities  Ensure data is integrated with other health metrics and that nothing is presented ‘in a vacuum’  Use data to target business-specific opportunities | See notes for senior management  have a draft scorecard, (overall health scorecard on an organizational level with snapshots of areas that are problematic with absence issues and costs), will continue to evolve  Sharing the draft will help determine the audience |
| Establish a culture where as part of a definition of high performance culture imbedded in this is the importance of a healthy organization  Develop a social support throughout the company – from top down around MH goals and objectives  Make MH and wellness part of managers objectives/ performance mgmt plan  There is an understanding /culture that all policies and procedures will be also measured and assessed on how they impact employee health | - Have employee health and productivity goals and value statements as part of the mission statement of the organization-connect health with wealth - Allow for flextime in order for employees to participate in wellness initiative. - Wellness initiatives should take place during company time - Select/train senior management to be health champions –role model - Develop/support a reward program for participation and health living - Clearly communicate vision, values and mission and make employee health integral part - Provide opportunities for employee development - Develop a healthy organizational road Map - Develop a national campaign around MH awareness with us as leader and sponsor- Best company in Canada in MH awareness and prevention- Model for other companies- use Mass media campaign | Include visionary ‘healthy workplace’ statements as part of the overall business strategy  Work with 3^rd^ party providers and internal stakeholders to brainstorm the form that social support networks could take…leveraging and integrating technology and tools wherever possible.  Mental health ‘app’? Relaxation app/program? Video? | Work is underway to align health and wealth, work will begin with aligning health objectives into employee value proposition. Longer process to link business objectives to health objectives. |
| **Objectives –Column one**  *What needs to be done / what needs to be changed?* | **Methods- Column two**  *How can these objectives be accomplished?* | |  |
|  | **Best practices and initial ideas** | **Big ideas** There are no wrong answers – think creatively!  *What would be the ideal activities to implement if there were no limitations (e.g. structural, organizational, financial, etc.)?* | **How can we implement this in our workplace and when could we implement?**  **If it’s already being done today, how can we improve it?**  **(Practical applications)** |
| Establish a climate that policies adheres and goes beyond regulations recommended by the commission  Demonstrate to employees that the organization cares and will be compassionate towards the MH issues and managers will be understanding  There is a climate that highly values training in wellness and MH and investment in social capital. Provides recognition for those that promote and encourage health living. |  | As above…start with incorporating healthy workplace and mental health objectives into business strategies and performance goals.  Provide dollar-value incentives for healthy lifestyle choices and/or demonstrated behaviour change. Personal Spending Account dollars?  Is there a connection to be made to benefits premium/discounting for good health metrics? (US companies already doing this) |  |
| Provide messages and resources to senior management to clarify expectations and procedures | - Delivering of messages from senior mgmt across to employees through avenues such as videos |  |  |

Mental Health and General

| **Objectives –Column one**  *What needs to be done / what needs to be changed?* | **Methods- Column two**  *How can these objectives be accomplished?* | |  |
| --- | --- | --- | --- |
|  | **Best practices and initial ideas** | **Big ideas** There are no wrong answers – think creatively!  *What would be the ideal activities to implement if there were no limitations (e.g. structural, organizational, financial, etc.)?* | **How can we implement this in our workplace and when could we implement?**  **If it’s already being done today, how can we improve it?**  **(Practical applications)** |
| **Family/partner/community** | | |  |
| Establish understanding of MH and role family/partner/community plays in reducing negative impact  Establish the belief that no one is immune to MH and that everyone should have access to the necessary information on how to help  Community establishes MH a high priority and provides necessary resources | - Make education/information on MH easily accessible and available - Media campaign –we are top company for MH and healthy workplace- The Standard by which all other companies are measured - Media Campaign sponsored by us to educate public - we includes family incentives to participate as well as employees | Establish a mobile app. increase the accessibility of information and tools for staff and all Canadians living with or supporting a loved one with mental illness  Have a team assess and build on the work from Mental Health Commission of Canada  Establish the “mental well being” competition and award. Invite grass roots establishments with great ideas targeting/addressing Mental Health.  We should explore the impact of using a third party vendor who specializes in mental health and send all their mental health claims to be managed. Potential impact to duration, Long Term Disability incidence and recidivism. |  |
| **Objectives –Column one**  *What needs to be done / what needs to be changed?* | **Methods- Column two**  *How can these objectives be accomplished?* | |  |
|  | **Best practices and initial ideas** | **Big ideas** There are no wrong answers – think creatively!  *What would be the ideal activities to implement if there were no limitations (e.g. structural, organizational, financial, etc.)?* | **How can we implement this in our workplace and when could we implement?**  **If it’s already being done today, how can we improve it?**  **(Practical applications)** |
| **Health care providers** | | |  |
| Highlight in community what is high quality care  Have available educational material that HCP can use to educate  Demonstrate positive social pressure in the community to stay up to date  The climate exists where follow-up and re-assessment is expected for quality care | - Educate employee as a means to inform HCP including holistic approach involve employees in decision process around treatment options- The Source, Benefit package etc. - With employee consent develop a communication package for HCP informing them we are willing to accommodate/assist in employee health needs - Employees educated on importance on follow-up care | Create HCP Wellness Councillors – who will help develop a strategy to educate, execute and establish centre for excellence. Create “best doctor” approach. |  |

Mental Health and General

| **Objectives –Column one**  *What needs to be done / what needs to be changed?* | **Methods- Column two**  *How can these objectives be accomplished?* | |  |
| --- | --- | --- | --- |
|  | **Best practices and initial ideas** | **Big ideas** There are no wrong answers – think creatively!  *What would be the ideal activities to implement if there were no limitations (e.g. structural, organizational, financial, etc.)?* | **How can we implement this in our workplace and when could we implement?**  **If it’s already being done today, how can we improve it?**  **(Practical applications)** |
| **Family/partner/community** | | |  |
| Establish understanding of MH and role family/partner/community plays in reducing negative impact  Establish the belief that no one is immune to MH and that everyone should have access to the necessary information on how to help  Community establishes MH a high priority and provides necessary resources | - Make education/information on MH easily accessible and available - We are top company for MH and healthy workplace- The Standard by which all other companies are measured - Media Campaign sponsored by us to educate public - We include family incentives to participate as well as employees | Create an event – trade show – sponsored by us where clients, families, employees, the public can attend, (trade fair) where we have organizations supporting MH in the community present, have booth, etc. to promote understanding/education of MH – give prizes and incentives, showcase our insight- have doctors. etc. speak but also people who are currently coping with their MH issue and share their journey  We to build/sponsor a MH facility that provides a wide range of services supporting employees, their families and the public in general  Sponsor a speaker series with doctors and their patients who can share their stories…i.e. to speak about what they experienced before diagnoses, during treatment, and now management of their condition. |  |
| **Objectives –Column one**  *What needs to be done / what needs to be changed?* | **Methods- Column two**  *How can these objectives be accomplished?* | |  |
|  | **Best practices and initial ideas** | **Big ideas** There are no wrong answers – think creatively!  *What would be the ideal activities to implement if there were no limitations (e.g. structural, organizational, financial, etc.)?* | **How can we implement this in our workplace and when could we implement?**  **If it’s already being done today, how can we improve it?**  **(Practical applications)** |
| **Health care providers** | | |  |
| Highlight in community what is high quality care  Have available educational material that HCP can use to educate  Demonstrate positive social pressure in the community to stay up to date  The climate exists where follow-up and re-assessment is expected for quality care | - Educate employee as a means to inform HCP including holistic approach involve employees in decision process around treatment options- The Source, Benefit package etc. - With employee consent develop a communication package for HCP informing them we are willing to accommodate/assist in employee health needs - Employees educated on importance on follow-up care | Arm employees with current treatment plans for their particular diagnoses as options to be discussed with their HCP to give employees and their families more control over choosing the next steps.  Continue to develop our Wellness Institute with MH material as a resource for the doctors, employees and their families |  |

**Cardiovascular/ diabetes/Musculo-skeletal/cancer/flu**

| **Objectives –Column one**  *What needs to be done / what needs to be changed?* | **Methods- Column two**  *How can these objectives be accomplished?* | |  |
| --- | --- | --- | --- |
|  | **Best practices and initial ideas** | **Big ideas** There are no wrong answers – think creatively!  *What would be the ideal activities to implement if there were no limitations (e.g. structural, organizational, financial, etc.)?* | **How can we implement this in our workplace and when could we implement?**  **If it’s already being done today, how can we improve it?**  **(Practical applications)** |
| **Employees** | | |  |
| Have information available and awareness of where to find it and understand that help/support is available (build knowledge, attitude &belief, expectations/self confidence.)  Proper medication use, make information available and know where to find it –awareness of drugs and interactions (build knowledge, attitude &belief, expectations/self confidence.)  Provide and awareness of resources on when to seek medical (HCP) attention, importance of follow-up and when family history is important (build knowledge, attitude & belief, expectations/self confidence) | - Make more information available on benefits site; information available on wellness centre through health library - Courses available for CPR training - The Source and wellness initiative emphasize take action with CV disease and diabetes - Use flex benefits - and microsite to educate - Encourage use of EAP communications - Include info on Source - Provide information on cancer treatments. | Annual/quarterly employee meetings that talk about benefit plan – a trending analysis or overview of plan (employees understand the cost of their benefit). Talk about top 5-10 drugs in the organization – build awareness and compassionate.  Quarterly meetings entirely devoted to health. Cross section of departments or divisions together with guest speakers – health fair – needs some form of point or reward system for attendance. Maybe personal spending account credits (would need to spend more time talking about personal spending accounts) or free tickets to health/fitness/sport related events (not a draw for one winner but everyone a winner)  When drug claim submitted online automatic email pop up box to member to advises of details to build knowledge  Annual Comprehensive Health Assessment/ Executive Health Plan – for ALL employees (annual or every couple years). Here is a Medcan example - multiple diagnostic tests, within a single 5-hour visit. (understand your medical history, conduct a thorough medical exam and synthesize the results of the diagnostic on-site tests). Objectives are to not only pick up early signs of disease, but also to give employee a strategy to improve health. At the end of Health Assessment, after discussing test results, employee and health team will work together to determine the best way to maintain or improve your health, and the changes you can make to help get there. |  |
| **Objectives –Column one**  *What needs to be done / what needs to be changed?* | **Methods- Column two**  *How can these objectives be accomplished?* | |  |
|  | **Best practices and initial ideas** | **Big ideas** There are no wrong answers – think creatively!  *What would be the ideal activities to implement if there were no limitations (e.g. structural, organizational, financial, etc.)?* | **How can we implement this in our workplace and when could we implement?**  **If it’s already being done today, how can we improve it?**  **(Practical applications)** |
| Understand the importance of repetitive strain and worksite ergonomics. Describe or explain what help is available to prevent repetitive strain.  build knowledge, attitude &belief, expectations/self confidence.)  Understand the importance of adapting the worksite to accommodate any limitations and/or risk. Acquire knowledge about regular rest breaks (build knowledge, attitude &belief, expectations/self confidence.)  Know how/when to get a ergonomic assessment  learn about prevention (colon/breast/skin/prostate/ lung cancers) |  | Make a one-time ergo assessment standard for all employees - some won’t ask – take away the ask and protects the employee privacy or need to disclose an issue  Computer pop-up to remind staff to stretch  Create stretch break walking routes within the building  Offer free fitness facility during core business hours and have staff there to lead rotating 5 minute stretch breaks during specific hours. Example 10 AM – 10:30 AM, 2 PM - 2:30 PM – 6 repeating - 5 minute stretch breaks  Provide reminded on Source – like a change your smoke detector with time change – get ergonomic assessment at a repetitive annual time  Many of the chronic medical conditions tend to have awareness months – bring in speakers, decorate work environment with information, have employee events, work with food service provider to off menu selection that promote prevention and have those items “free” during the awareness campaign |  |
| **Objectives –Column one**  *What needs to be done / what needs to be changed?* | **Methods- Column two**  *How can these objectives be accomplished?* | |  |
|  | **Best practices and initial ideas** | **Big ideas** There are no wrong answers – think creatively!  *What would be the ideal activities to implement if there were no limitations (e.g. structural, organizational, financial, etc.)?* | **How can we implement this in our workplace and when could we implement?**  **If it’s already being done today, how can we improve it?**  **(Practical applications)** |
| Explain that there are resources available that will provide you with information about the flu and the flu shot to make decision that is right for them. Learn about prevention, know where to seek information about prevention  Provide knowledge and build positive attitude and beliefs, expectations and self confidence for decision making  Acquire knowledge on hand washing and other sanitary practices.  Know where to find necessary information Provide knowledge and build positive attitude and beliefs, expectations and self confidence |  | Host department breakfast events with a focus on food choices that prevent flu and cold. Before flu season or when flu shots first being talked about  Have material on hand so there is a connection between the food event and the health event  Create e-learning opportunity – information shared, knowledge tested and prize awarded  Have cleaning supplies readily available on each desk – send reminders to clean surfaces.  Have cleaning staff clean surfaces frequently |  |
| **Objectives –Column one**  *What needs to be done / what needs to be changed?* | **Methods- Column two**  *How can these objectives be accomplished?* | |  |
|  | **Best practices and initial ideas** | **Big ideas** There are no wrong answers – think creatively!  *What would be the ideal activities to implement if there were no limitations (e.g. structural, organizational, financial, etc.)?* | **How can we implement this in our workplace and when could we implement?**  **If it’s already being done today, how can we improve it?**  **(Practical applications)** |
| **Managers** | | |  |
| Incorporate manager training (cardio/diabetes/MSK/cancer) accountability in procedures-how to accommodate/how to seek help/where to seek help and develop positive relationships (norm/policy)  Senior management demonstrate positive recognition of managers (norm)  Managers need to know where they can find help and procedures how to deal with health problems impacting work (build knowledge, attitude & belief, expectations/ self confidence)  Provide messaging that it is acceptable to seek help  Inform that it is the norm (policy) that all managers/supervisors obtain training on best practices on how to deal flu | - Wellness as an agenda meeting item in some of the leaders meetings held by department heads in order to discuss expectations and answer questions | Have full-time health professional on staff to speak to leaders at meetings or 1:1 and also to attend team meetings if needed.  Assign a wellness champion on each team that is a monetarily compensated (salary or bonus) and has work load adjusted to devote time to team health initiatives  Quarterly meetings entirely devoted to health. Cross section of departments or divisions together with guest speakers |  |

**Cardiovascular/ diabetes/Musculoskeletal/cancer/flu**

| **Objectives –Column one**  *What needs to be done / what needs to be changed?* | **Methods- Column two**  *How can these objectives be accomplished?* | |  |
| --- | --- | --- | --- |
|  | **Best practices and initial ideas** | **Big ideas** There are no wrong answers – think creatively!  *What would be the ideal activities to implement if there were no limitations (e.g. structural, organizational, financial, etc.)?* | **How can we implement this in our workplace and when could we implement?**  **If it’s already being done today, how can we improve it?**  **(Practical applications)** |
| **Employees** | | |  |
| Have information available and awareness of where to find it and understand that help/support is available (build knowledge, attitude &belief, expectations/self confidence.)  Proper medication use, make information available and know where to find it –awareness of drugs and interactions (build knowledge, attitude &belief, expectations/self confidence.)  Provide and awareness of resources on when to seek medical (HCP) attention, importance of follow-up and when family history is important (build knowledge, attitude & belief, expectations/self confidence) | - Make more information available on benefits site; information available on wellness centre through health library - Courses available for CPR training - The Source and wellness initiative emphasize take action with CV disease and diabetes - Use flex benefits - and microsite to educate - Encourage use of EAP communications - Include info on Source - Provide information on cancer treatments. | Create a wellness website to house more information specifically related to CVD, diabetes, MSK, cancer, flu – make information more easily accessible via an external URL so that employees can access at any time (not restricted to benefits site)  Have a health advisor at that is a direct resource for information around EAP, disease, treatment, HCPs, etc. (like having an on-site nurse but accessible online for all employees across Canada) |  |
| **Objectives –Column one**  *What needs to be done / what needs to be changed?* | **Methods- Column two**  *How can these objectives be accomplished?* | |  |
|  | **Best practices and initial ideas** | **Big ideas** There are no wrong answers – think creatively!  *What would be the ideal activities to implement if there were no limitations (e.g. structural, organizational, financial, etc.)?* | **How can we implement this in our workplace and when could we implement?**  **If it’s already being done today, how can we improve it?**  **(Practical applications)** |
| Understand the importance of repetitive strain and worksite ergonomics. Describe or explain what help is available to prevent repetitive strain. build knowledge, attitude &belief, expectations/self confidence.)  Understand the importance of adapting the worksite to accommodate any limitations and/or risk. Acquire knowledge about regular rest breaks (build knowledge, attitude &belief, expectations/self confidence.)  Know how/when to get a ergonomic assessment  learn about prevention (colon/breast/skin/prostate/ lung cancers) |  | Provide ergonomic assessments to all employees  Provide more information about cancer screenings (what is recommended, what should be required at certain ages, etc.) |  |
| **Objectives –Column one**  *What needs to be done / what needs to be changed?* | **Methods- Column two**  *How can these objectives be accomplished?* | |  |
|  | **Best practices and initial ideas** | **Big ideas** There are no wrong answers – think creatively!  *What would be the ideal activities to implement if there were no limitations (e.g. structural, organizational, financial, etc.)?* | **How can we implement this in our workplace and when could we implement?**  **If it’s already being done today, how can we improve it?**  **(Practical applications)** |
| Explain that there are resources available that will provide you with information about the flu and the flu shot to make decision that is right for them. Learn about prevention, know where to seek information about prevention  Provide knowledge and build positive attitude and beliefs, expectations and self confidence for decision making  Acquire knowledge on hand washing and other sanitary practices.  Know where to find necessary information Provide knowledge and build positive attitude and beliefs, expectations and self confidence |  | Send a survey to ask employees what information they would like to see or have focus groups if there have been too many surveys sent out  Ask open-ended questions – how can we help you develop your knowledge and self-confidence regarding health issues?  Create a ‘health & wellness’ centre at all offices (not just head office locations) with community resources and information that is updated on a regular basis to increase knowledge and self-confidence (this can also be established by creation of a wellness website) |  |
| **Objectives –Column one**  *What needs to be done / what needs to be changed?* | **Methods- Column two**  *How can these objectives be accomplished?* | |  |
|  | **Best practices and initial ideas** | **Big ideas** There are no wrong answers – think creatively!  *What would be the ideal activities to implement if there were no limitations (e.g. structural, organizational, financial, etc.)?* | **How can we implement this in our workplace and when could we implement?**  **If it’s already being done today, how can we improve it?**  **(Practical applications)** |
| **Managers** | | |  |
| Incorporate manager training (cardio/diabetes/MSK/cancer) accountability in procedures-how to accommodate/how to seek help/where to seek help and develop positive relationships (norm/policy)  Senior management demonstrate positive recognition of managers (norm)  Managers need to know where they can find help and procedures how to deal with health problems impacting work (build knowledge, attitude & belief, expectations/ self confidence)  Provide messaging that it is OK to seek help  Inform that it is the norm (policy) that all managers/supervisors obtain training on best practices on how to deal flu | - Wellness as an agenda meeting item in some of the leaders meetings held by department heads in order to discuss expectations and answer questions | Make manager training regarding CVD/diabetes/MSK/cancer mandatory – how to support employees with prevention, how to identify potential issues, how to provide support for someone going off/on leave, etc.  Create a space on The Source where stories are shared about seeking help  Create a forum for managers to discuss successes and questions around providing help to team members  Make expectations and positive recognition from senior management explicit by publishing guidelines and stories on The Source |  |

| **Objectives –Column one**  *What needs to be done / what needs to be changed?* | **Methods- Column two**  *How can these objectives be accomplished?* | |  |
| --- | --- | --- | --- |
|  | **Best practices and initial ideas** | **Big ideas** There are no wrong answers – think creatively!  *What would be the ideal activities to implement if there were no limitations (e.g. structural, organizational, financial, etc.)?* | **How can we implement this in our workplace and when could we implement?**  **If it’s already being done today, how can we improve it?**  **(Practical applications)** |
| **Senior Management** | | |  |
| Expectation that there will be a movement within the company to provide necessary investment in management/prevention of CV disease/diabetes at workplace  Understand important role and learn skills to lead by example  Provide messaging that it is OK to seek help and we will will support you  Continue to move toward mandatory education for all managers to enhance knowledge, capability, skill in the area of CV health.(policy) | - Share stories after training that demonstrates how managers were able to help employees / felt better equipped to support the team due to the training - Delivering of messages from senior mgmt across to employees through avenues such as videos - Integrate all health and wellness programs and initiatives - Assure confidentiality to encourage participation in wellness initiatives and do not discriminate due to health status - Use gift certificate rewards - Benefits incentives-cash accounts as incentives - Describe through various communications (narratives/stories) about how seeking help/communication with managers was a positive experience | 1. track organizational health metrics and report on those metrics in senior leadership forums (Executive Team, Leadership team, Business Unit Leadership Teams, etc. 2. Take the above one step further and have us commit to publishing their health metrics in investor facing material (i.e. annual report, Wellness Institute) 3. Employees at all levels share their health journeys—successes, failures, learnings |  |
| **Objectives –Column one**  *What needs to be done / what needs to be changed?* | **Methods- Column two**  *How can these objectives be accomplished?* | |  |
|  | **Best practices and initial ideas** | **Big ideas** There are no wrong answers – think creatively!  *What would be the ideal activities to implement if there were no limitations (e.g. structural, organizational, financial, etc.)?* | **How can we implement this in our workplace and when could we implement?**  **If it’s already being done today, how can we improve it?**  **(Practical applications)** |
| Use benchmarking as a means to provide feedback on how we are doing relative to other companies and relative to past measures (reinforcement)  Use the data to help facilitate learning and change throughout the business (managers) (reinforcement)  There is an understanding that all policies and procedures will be also measured and assessed on how they impact employee health  Describe a work culture of that encourages cancer prevention and assessment f risk factors  business continuity (e.g. flu pandemic) - having a plan in place for what the organization should do | - Describe how at various points of interaction employees (interviews, employee handbook, The Source where employee receive messaging about communicating limitations with managers - use methods available through the source for positive recognition (i.e. sending a VIP) - provide the flu shot clinic for those who are interested in getting the shot |  |  |
| **Objectives –Column one**  *What needs to be done / what needs to be changed?* | **Methods- Column two**  *How can these objectives be accomplished?* | |  |
|  | **Best practices and initial ideas** | **Big ideas** There are no wrong answers – think creatively!  *What would be the ideal activities to implement if there were no limitations (e.g. structural, organizational, financial, etc.)?* | **How can we implement this in our workplace and when could we implement?**  **If it’s already being done today, how can we improve it?**  **(Practical applications)** |
| **Partner/family/community** | | |  |
| Establish understanding of CV/diabetes/MSK/Cancer/flu when to seek help and role family/partner/ community plays in reducing negative impact and where can get necessary resources (build knowledge, attitude &belief, expectations/self confidence.)  Make resources available on narcotic use for MSK pain | - There is a means to establish awareness through sharing of stories and highlighted in community events | 1. to sponsor events that support research (cancer, diabetes, etc.). Marathons, 5K runs, cycling events. Similar to the CIBC Run for the Cure and the Shoppers Drug Mart Weekend to End Women’s Cancers. These can be local or National in nature. 2. sponsor information booths at events such as described above. Information provided could focus on Wellness and prevention. May also include information on how to be prepared financially in the event of a health event |  |
| **Health care provider** | | |  |
| Describe the norm where the HCP, employee and workplace communicate effectively and work together to maximize health of employee  Describe the expectation that specific work limitation/modification be communicated with the workplace | Share best practices with employee and HCP using communication form employee takes to HCP | 1. partner with universities / medical schools in support of targeted research. 2. partner with specific hospitals (i.e. Princess Margaret) to develop outpatient / former patient programs with a focus on the impact of returning to work after a serious health event. |  |

**Mental health/ Cardiovascular/ diabetes/Musculoskeletal/cancer/flu**

| **Objectives –Column one**  *What needs to be done / what needs to be changed?* | | | **Methods- Column two**  *How can these objectives be accomplished?* | | |  |
| --- | --- | --- | --- | --- | --- | --- |
|  |  |  | **Best practices and initial ideas** | | **Big ideas** There are no wrong answers – think creatively!  *What would be the ideal activities to implement if there were no limitations (e.g. structural, organizational, financial, etc.)?* | **How can we implement this in our workplace and when could we implement?**  **If it’s already being done today, how can we improve it?**  **(Practical applications)** |
| **Regular Exercise**   - Explain how exercise is beneficial to health (knowledge) - Can find 30 min/day to exercise (attitude & belief - Expect to exercise (expectations) - Confidence that can find/do 30 min/day - mentorship by management; role models for exercise (norms), peers, family - social support for exercise/peers/ management/ family - reward positive behaviours (reinforcement) - management provides sufficient resources - Daily exercise is part of the organizational culture and is demonstrated through the actions of senior management | | | - Use videos (e.g. 23 1/2 hours) - Wellness programs such as health challenges - Reinforce programs such as Weight Watchers at Work, fitness reimbursements, Wellness Account, on-site gyms - Develop social networks - Access to free gym facilities-have volunteer on-site exercise facility –yoga/spin class - Use pedometer program-team challenges - Use verbal reinforcement and reward positive behaviour - sharing of best practices (e.g. collaboration tool or forum to share what employees/managers are doing) - senior leaders demonstrate participating in national wellness day and other wellness programs offered | | “Make regular Exercise part of your life” campaign   - mass communication strategy - put up posters in every building on every floor with “Did you knows” and facts on regular exercise. Include some scary facts (similar to tobacco packaging). Put up signs at the elevators telling people to take the healthy route (the stairs). - Allow flexibility in work schedules so employees can attend fitness classes at lunch, etc. - Posters for desks telling employees to take a 1 minute walk/stretch break every hour - Desk drop with work out bands and exercise instructions people can post at their desks. - Computer drop of daily calendars with different 15 minute workout ideas or challenges for employees - Add walking treadmill workstations (1 or 2 per floor). Employees can book out treadmill workstations in 30 minute time blocks to walk while they work.   Corporate Commitment to Fitness … Employer sponsored registration in one fitness event of your choice (from list of eligible events), and discounts for one other friend or family member to participate with them.   - have different choices for employees based on different ability levels (i.e. beginner, intermediate, advanced) - have different activities in each level to appeal to all types of exercise (walking, running, biking, triathalon, swimming, cross fit, tough mudders, dance, etc.) - employees get incentives to signing up for program (discounts on athletic gear, recognition by leaders if they complete the activities successfully) - each activity has a leader assigned to it, and have leaders send the training guide to people signed up in their activities - have a paid professional or volunteer organize free training sessions at the office (i.e. 30 minute daily walks or runs at 7am, 12pm, or 5pm) |  |
| **Objectives –Column one**  *What needs to be done / what needs to be changed?* | | | **Methods- Column two**  *How can these objectives be accomplished?* | | |  |
|  |  |  | **Best practices and initial ideas** | | **Big ideas** There are no wrong answers – think creatively!  *What would be the ideal activities to implement if there were no limitations (e.g. structural, organizational, financial, etc.)?* | **How can we implement this in our workplace and when could we implement?**  **If it’s already being done today, how can we improve it?**  **(Practical applications)** |
| **Adequate Sleep**   - Describe the recommended sleep requirement (guidelines) and quality vs. quantity of sleep (knowledge) - Build positive attitude, confidence and expectation around adequate sleep - Awareness of resources - Provide feedback using HRA assessment (reinforcement) - Part of the organizational culture that sleep matters as part of a healthy & high performance organization and is demonstrated through the actions of senior management - Mentorship and social support from management, peers, family | | | - EAP program; sleep webinars as part of wellness program; Health Assessment sleep data for analysis to see if it's going in the right direction over time | | Make sleep a priority in the workplace.   - Allow employees to use their breaks to have 15 minute naps. Have rooms they can book out for napping. Create onsite facilities for employees to use when they need to rest with beds, couches, soothing music, etc. - Consider purchasing one or two napping pods that employees can book - Set up discounts at spas or get a preferred provider for massages. - Have regular sleep webinars - Give employees journals or notepads they can keep in their bedrooms to write down anything that is keeping them up at night - Sleep poster campaign in areas where caffeine is sold with theme “Are you getting enough sleep?” (i.e. Tim Hortons, Vending Machines, etc.) - Have senior leaders do a spiel in their quarterly meetings about importance of adequate sleep, and include information about EAP program and sleep support services.   “What’s keeping you up at night” forum or conversation on IdeaShare.   - Employees, senior leaders, etc. encouraged to share stories about what’s keeping them up at night on IdeaShare. Other employees would be able to comment and try to provide advice and support to each other to solve each other’s problems. - We could do this in line with a “Sleep is a necessity” campaign and share facts on the Source, do webinars, encourage use of the EAP, etc. |  |
| **Objectives –Column one**  *What needs to be done / what needs to be changed?* | | | **Methods- Column two**  *How can these objectives be accomplished?* | | |  |
|  |  |  | **Best practices and initial ideas** | | **Big ideas** There are no wrong answers – think creatively!  *What would be the ideal activities to implement if there were no limitations (e.g. structural, organizational, financial, etc.)?* | **How can we implement this in our workplace and when could we implement?**  **If it’s already being done today, how can we improve it?**  **(Practical applications)** |
| **Work/life Balance**  **Stress management**   - Learn how to better manage time and stress where to find available resources (knowledge) - Build positive attitude, confidence and expectation around life work balance/stress management - Awareness of resources - Provide feedback using HRA (reinforcement) - Mentorship among managers/peers/family (norm) - There is social support among managers/peers /family - It is the organizational culture that life/work balance and stress management are important components of a healthy & high performance organization and is demonstrated through the actions of senior management | | | - Describe flex time schedule to enable employees to accommodate home situation and have time flexibility - Encourage telecommuting and use of flex hours - On The Source have stories/testimonials that may have an effect to model other to achieve the same - Lunch and learn - Promote the EAP as resource to get help - The source for information and awareness | | “Take a break” commitment & become a leader in work-life balance   - Share literature on importance of taking breaks to reduce stress - Campaign around doing at least one stress relieving activity in a day. Provide ideas to employees through the intranet and create IdeaShare where employees share and comment on each other’s ideas on ways to reduce stress. Incorporate a contest or challenge. - Have leaders send 5 minute Friday funnies to encourage employees to take a small break to make them more productive when they start working again. - Have leaders organize weekly lunches with their teams so they can take breaks together - Continue to allow flex time and continue accommodating employees - Promote employee use of the EAP through mass marketing campaign - Have senior leaders share blogs about what they do for work-life balance - Set up staff rooms with games where employees can go to refresh and take a break - Reward employees with work-from-home days |  |
| **Objectives –Column one**  *What needs to be done / what needs to be changed?* | | | **Methods- Column two**  *How can these objectives be accomplished?* | | |  |
|  |  |  | **Best practices and initial ideas** | **Big ideas** There are no wrong answers – think creatively!  *What would be the ideal activities to implement if there were no limitations (e.g. structural, organizational, financial, etc.)?* | | **How can we implement this in our workplace and when could we implement?**  **If it’s already being done today, how can we improve it?**  **(Practical applications)** |
| **Proper diet/nutrition**   - Explain how one can achieve adequate nutrition and impact of health and where to get information (Knowledge) - Develop positive attitude/belief/expectations and self confidence in ability to eat healthy - Role model s and social support for proper eating managers/peers/ family (norms) - discuss community norms shifting toward healthy food/drink choices and where to find resources - It is the organizational culture that proper eating matters is an important part of a healthy & high performance organization and is demonstrated through the actions of senior management - Social support for healthy living can provide positive reinforcement | | | - collaboration spaces or forums to share success stories; profile leaders favorite recipes - emphasize CEO messaging around being a high performance culture and the type of climate we're striving for and how we're going to get there; ensure employees know that a positive culture is important - Provide more option in drinks like juices rather than coffee or pop - healthy cafeteria food; information on the wellness -part of the Source website e.g. healthy recipes - develop social networks - regular biometric screening | Priority on Healthy Eating   - have a senior leader(s) join Weight Watchers and attend Weight Watchers at Work meetings - leader profiles on healthy eating on the Intranet - teams encouraged to have healthy potluck once a month where teammates bring in healthy food items - Favourite Food Fridays – every week on Friday a senior leader sends a healthy recipe. Samples could be available in the cafeteria. - Healthy Food Challenge/Contest: held in major office locations, people enter the healthy food contest and cook their healthy dishes to be judged by staff. Staff could pay for admission or for food tokens, and all the money raised could go to a healthy charity. - All corporate events, training sessions, etc. feature a healthy menu (only healthy snacks allowed) - Have nutrition labels at vending machines and have nutrition content information available for the cafeteria menu - Make unhealthy foods more expensive in the cafeteria so we can subsidize healthy options | |  |
| **Objectives –Column one**  *What needs to be done / what needs to be changed?* | **Methods- Column two**  *How can these objectives be accomplished?* | | | | |  |
|  | **Best practices and initial ideas** | | | **Big ideas** There are no wrong answers – think creatively!  *What would be the ideal activities to implement if there were no limitations (e.g. structural, organizational, financial, etc.)?* | | **How can we implement this in our workplace and when could we implement?**  **If it’s already being done today, how can we improve it?**  **(Practical applications)** |
| **Avoid tobacco**  **Avoid excessive alcohol**   - Describe how these can impact health and where to find help (knowledge) - Develop positive attitude/belief/ expectations and self confidence in ability to stop smoking and avoid excessive alcohol - Role models for healthy living managers /peers/family - It is the organizational culture that life/work balance and stress management matters and is an important part of a healthy & high performance organization and is demonstrated through the actions of senior management | | - Enforce policy smoking around building (second hand smoke) - Leverage using the Source with other community programs/initiate - Demonstrate how EAP can be used to assist employees | | Cold Turkey Campaign   - In line with timing for New Year’s Eve and New Year’s resolutions - Encourage all employees to give up something they are addicted to (i.e. smoking, wine, coffee, etc.) and reward them for sticking to their plan (every day they would get a star). If they go one month (or specified time) without “cheating” they would get an extra $20 added to their PSA account. - Have leaders or other employees post their experiences on the Source - Set up weekly online support groups/webinars that people can attend for 30 minutes and share their struggles and triumphs - Make no-smoking a priority and create anonymous forum on quitting smoking where employees can share their stories/challenges/experiences and people can give each other advice and support - Set up EAP webinars for education on quitting addictions - Educate employees about EAP in Source postings and targeted emails. | |  |

**Mental health/ Cardiovascular/ diabetes/Musculoskeletal/cancer/flu**

| **Objectives –Column one**  *What needs to be done / what needs to be changed?* | | | **Methods- Column two**  *How can these objectives be accomplished?* | |  |
| --- | --- | --- | --- | --- | --- |
|  |  |  | **Best practices and initial ideas** | **Big ideas** There are no wrong answers – think creatively!  *What would be the ideal activities to implement if there were no limitations (e.g. structural, organizational, financial, etc.)?* | **How can we implement this in our workplace and when could we implement?**  **If it’s already being done today, how can we improve it?**  **(Practical applications)** |
| **Regular Exercise**   - Explain how exercise is beneficial to health (knowledge) - Can find 30 min/day to exercise (attitude & belief - Expect to exercise (expectations) - Confidence that can find/do 30 min/day - mentorship by management; role models for exercise (norms), peers, family - social support for exercise/peers/ management/ family - reward positive behaviours (reinforcement) - management provides sufficient resources - Daily exercise is part of the organizational culture and is demonstrated through the actions of senior management | | | - Use videos (e.g. 23 1/2 hours) - Wellness programs such as health challenges - Reinforce programs such as Weight Watchers at Work, fitness reimbursements, Wellness Account, on-site gyms - Develop social networks - Access to free gym facilities-have volunteer on-site exercise facility –yoga/spin class - Use pedometer program-team challenges - Use verbal reinforcement and reward positive behaviour - sharing of best practices (e.g. collaboration tool or forum to share what employees/managers are doing) - senior leaders demonstrate participating in national wellness day and other wellness programs offered | Remodel the working environment. Cubicles should not be Cubicles (Follow the example of Google & Apple).  On site Gym – game room (fitness, On- line bowling, boxing).  Rest Stations |  |
| **Objectives –Column one**  *What needs to be done / what needs to be changed?* | | | **Methods- Column two**  *How can these objectives be accomplished?* | |  |
|  |  |  | **Best practices and initial ideas** | **Big ideas** There are no wrong answers – think creatively!  *What would be the ideal activities to implement if there were no limitations (e.g. structural, organizational, financial, etc.)?* | **How can we implement this in our workplace and when could we implement?**  **If it’s already being done today, how can we improve it?**  **(Practical applications)** |
| **Adequate Sleep**   - Describe the recommended sleep requirement (guidelines) and quality vs. quantity of sleep (knowledge) - Build positive attitude, confidence and expectation around adequate sleep - Awareness of resources - Provide feedback using HRA assessment (reinforcement) - Part of the organizational culture that sleep matters as part of a healthy & high performance organization and is demonstrated through the actions of senior management - Mentorship and social support from management, peers, family | | | - EAP program; sleep webinars as part of wellness program; HRA sleep data for analysis to see if it's going in the right direction over time | Work from home (facilitates productivity)  Flexible work week hours (must be voluntary)  Workshops EAP – Coaching sessions. |  |
| **Objectives –Column one**  *What needs to be done / what needs to be changed?* | | | **Methods- Column two**  *How can these objectives be accomplished?* | |  |
|  |  |  | **Best practices and initial ideas** | **Big ideas** There are no wrong answers – think creatively!  *What would be the ideal activities to implement if there were no limitations (e.g. structural, organizational, financial, etc.)?* | **How can we implement this in our workplace and when could we implement?**  **If it’s already being done today, how can we improve it?**  **(Practical applications)** |
| **Work/life Balance**  **Stress management**   - Learn how to better manage time and stress where to find available resources (knowledge) - Build positive attitude, confidence and expectation around life work balance/stress management - Awareness of resources - Provide feedback using HRA (reinforcement) - Mentorship among managers/peers/family (norm) - There is social support among managers/peers /family - It is the organizational culture that life/work balance and stress management are important components of a healthy & high performance organization and is demonstrated through the actions of senior management | | | - Describe flex time schedule to enable employees to accommodate home situation and have time flexibility - Encourage telecommuting and use of flex hours - On The Source have stories/testimonials that may have an effect to model other to achieve the same - Lunch and learn - Promote the EAP as resource to get help - The source for information and awareness | “Call between us” – sharing experiences work or home . What is discussed stays confidential.  Regular interaction with Management/Staff.  Annual work family day.  One Mental health day (not during peak periods) but that one-day will make a difference. |  |
| **Objectives –Column one**  *What needs to be done / what needs to be changed?* | | | **Methods- Column two**  *How can these objectives be accomplished?* | |  |
|  |  |  | **Best practices and initial ideas** | **Big ideas** There are no wrong answers – think creatively!  *What would be the ideal activities to implement if there were no limitations (e.g. structural, organizational, financial, etc.)?* | **How can we implement this in our workplace and when could we implement?**  **If it’s already being done today, how can we improve it?**  **(Practical applications)** |
| **Proper diet/nutrition**   - Explain how one can achieve adequate nutrition and impact of health and where to get information (Knowledge) - Develop positive attitude/belief/expectations and self confidence in ability to eat healthy - Role model s and social support for proper eating managers/peers/ family (norms) - discuss community norms shifting toward healthy food/drink choices and where to find resources - It is the organizational culture that proper eating matters is an important part of a healthy & high performance organization and is demonstrated through the actions of senior management - Social support for healthy living can provide positive reinforcement | | | - collaboration spaces or forums to share success stories; profile leaders favorite recipes - emphasize CEO messaging around being a high performance culture and the type of climate we're striving for and how we're going to get there; ensure employees know that a positive culture is important - Provide more option in drinks like juices rather than coffee or pop - healthy cafeteria food; information on the wellness -part of the Source website e.g. healthy recipes - develop social networks - regular biometric screening | Bring back on-site cafeteria – healthy preparation of food…(have an honor system, what you feel the meal will cost the employees pays and at the end of the year the money goes to a charity of choice).  Nutrition campaign.  Social Evenings (once a month) |  |
| **Objectives –Column one**  *What needs to be done / what needs to be changed?* | **Methods- Column two**  *How can these objectives be accomplished?* | | | |  |
|  | **Best practices and initial ideas** | | | **Big ideas** There are no wrong answers – think creatively!  *What would be the ideal activities to implement if there were no limitations (e.g. structural, organizational, financial, etc.)?* | **How can we implement this in our workplace and when could we implement?**  **If it’s already being done today, how can we improve it?**  **(Practical applications)** |
| **Avoid tobacco**  **Avoid excessive alcohol**   - Describe how these can impact health and where to find help (knowledge) - Develop positive attitude/belief/ expectations and self confidence in ability to stop smoking and avoid excessive alcohol - Role models for healthy living managers /peers/family - It is the organizational culture that life/work balance and stress management matters and is an important part of a healthy & high performance organization and is demonstrated through the actions of senior management | | - Enforce policy smoking around building (second hand smoke) - Leverage using the Source with other community programs/initiate - Demonstrate how EAP can be used to assist employees | | “No smoke week” – Give a gift to person who holds off smoking.  Company pays for a one-time quit smoking program. (the choice of when to quit). If the employee is successful a donation can be made to the Cancer Research. |  |

GB= Group Benefits, PSA= Personal Spending Account, MH= Mental Health, EAP= Employee Assistance Program, EAAP= Employee Assistance and Advice Program, PMP= Performance Management Program, HRA= Health Risk Assessment, CV= Cardiovascular, MSK= Musculoskeletal, HCP= Healthcare Provider, VP= Vice President, SVP= Senior Vice President, LTD= Long Term Disability, Mgmt= Management.
